# New data towards the development of a comprehensive taphonomic framework for the Late Jurassic Cleveland-Lloyd Dinosaur Quarry, Central Utah

**DOI:** 10.7717/peerj.3368

**Published:** 2017-06-06

**Authors:** Joseph E. Peterson, Jonathan P. Warnock, Shawn L. Eberhart, Steven R. Clawson, Christopher R. Noto

**Affiliations:** 1 Department of Geology, University of Wisconsin-Oshkosh, Oshkosh, WI, United States of America; 2 Department of Geoscience, Indiana University of Pennsylvania, Indiana, PA, United States of America; 3 Department of Entomology, University of Wisconsin-Madison, Madison, WI, United States of America; 4 Department of Biological Sciences, University of Wisconsin-Parkside, Kenosha, WI, United States of America

**Keywords:** Paleoecology, Geochemistry, Morrison, Bonebed

## Abstract

The Cleveland-Lloyd Dinosaur Quarry (CLDQ) is the densest deposit of Jurassic theropod dinosaurs discovered to date. Unlike typical Jurassic bone deposits, it is dominated by the presence of *Allosaurus fragilis*. Since excavation began in the 1920s, numerous hypotheses have been put forward to explain the taphonomy of CLDQ, including a predator trap, a drought assemblage, and a poison spring. In an effort to reconcile the various interpretations of the quarry and reach a consensus on the depositional history of CLDQ, new data is required to develop a robust taphonomic framework congruent with all available data. Here we present two new data sets that aid in the development of such a robust taphonomic framework for CLDQ. First, x-ray fluorescence of CLDQ sediments indicate elevated barite and sulfide minerals relative to other sediments from the Morrison Formation in the region, suggesting an ephemeral environment dominated by periods of hypereutrophic conditions during bone accumulation. Second, the degree of abrasion and hydraulic equivalency of small bone fragments dispersed throughout the matrix were analyzed from CLDQ. Results of these analyses suggest that bone fragments are autochthonous or parautochthonous and are derived from bones deposited in the assemblage rather than transported. The variability in abrasion exhibited by the fragments is most parsimoniously explained by local periodic re-working and re-deposition during seasonal fluctuations throughout the duration of the quarry assemblage. Collectively, these data support previous interpretations that the CLDQ represents an attritional assemblage in a poorly-drained overbank deposit where vertebrate remains were introduced post-mortem to an ephemeral pond during flood conditions. Furthermore, while the elevated heavy metals detected at the Cleveland-Lloyd Dinosaur Quarry are not likely the primary driver for the accumulation of carcasses, they are likely the result of multiple sources; some metals may be derived from post-depositional and diagenetic processes, and others are potentially produced from an abundance of decomposing vertebrate carcasses. These new data help to support the inferred depositional environment of the quarry as an ephemeral pond, and represent a significant step in understanding the taphonomy of the bonebed and Late Jurassic paleoecology in this region.

## Introduction

**Figure 1:**
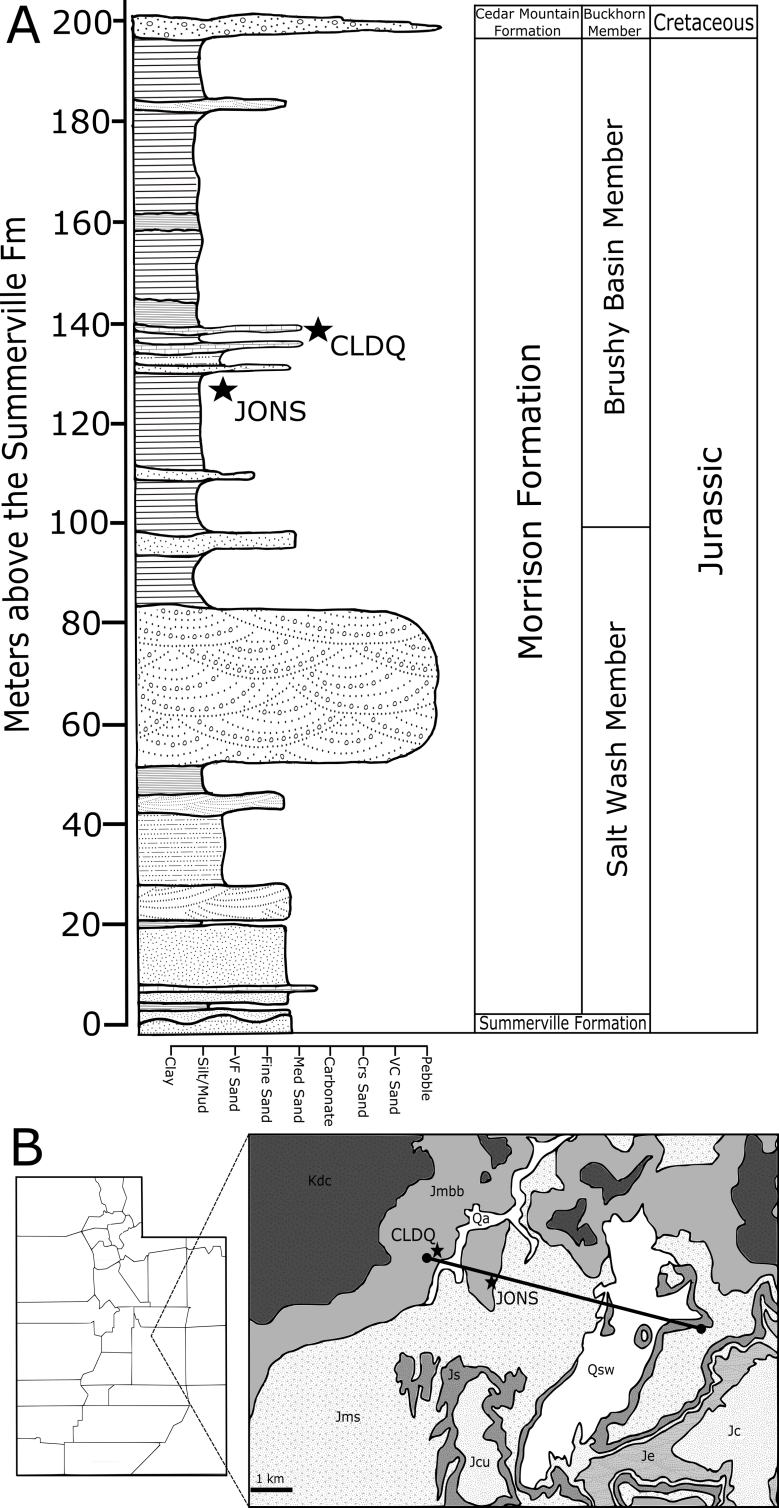
Regional stratigraphy of the CLDQ vicinity. (A) Stratigraphic column of the Morrison Formation in the area around the Cleveland-Lloyd Dinosaur Quarry (CLDQ) and the Johnsonville (JONS) sites, shown in meters above the basal contact of the Salt Wash Member of the Morrison Formation with the upper Summerville Formation. Standard USGS symbols of rock units are used in the diagram. (B) Map showing sites, stratigraphic section line, and regional stratigraphy in context of the San Rafael Swell.

**Figure 2:**
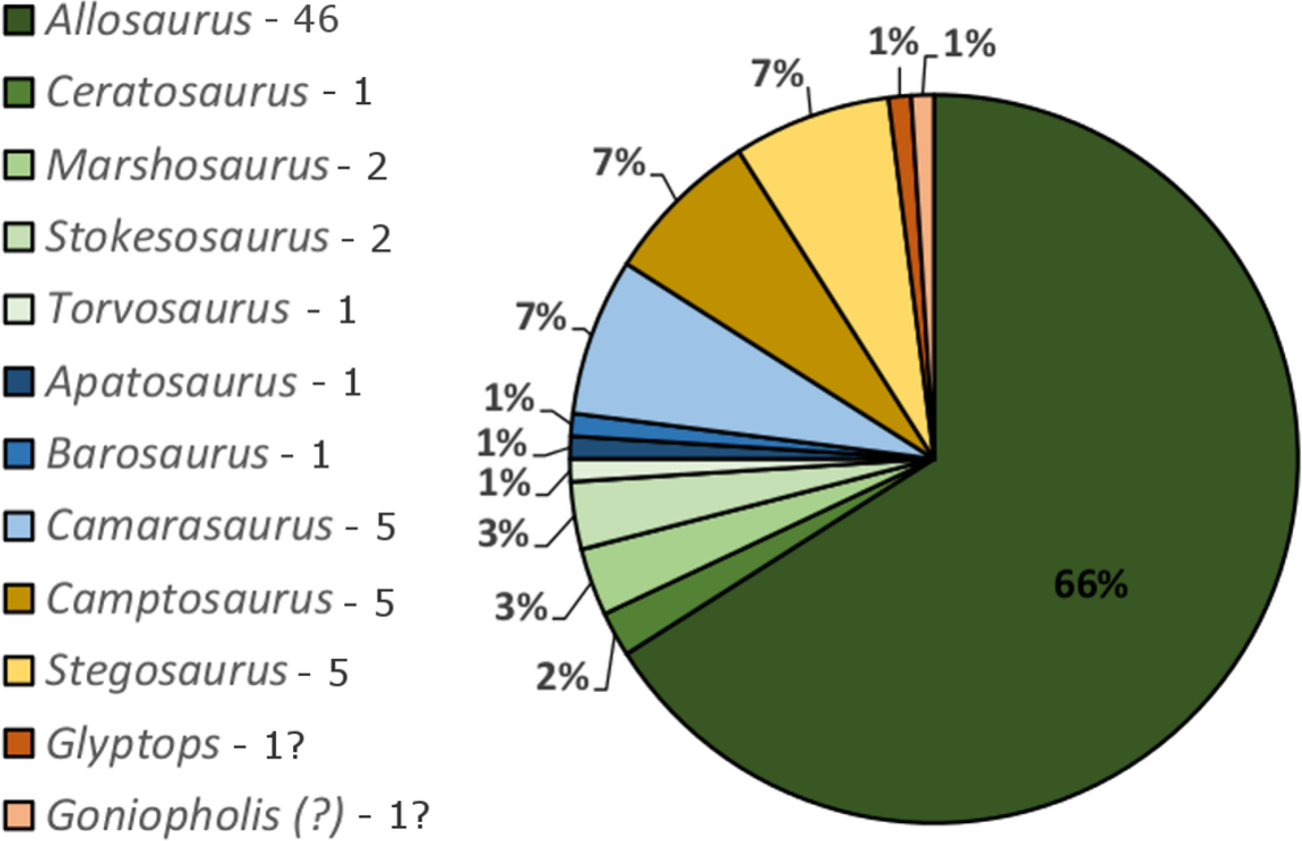
Vertebrate fauna of the Cleveland-Lloyd Dinosaur Quarry. Vertebrate fauna of the Cleveland-Lloyd Dinosaur Quarry, illustrating the 3:1 ratio of predators to prey and minimum number of individuals for each taxon, based on left femoral count. Modified from Gates, 2005.

The Cleveland-Lloyd Dinosaur Quarry (CLDQ) of central Utah is located in the Brushy Basin Member of the Upper Jurassic Morrison Formation at the northern end of the San Rafael Swell (Figs. 1A, 1B). The quarry is world-famous for its unusually high concentration of dinosaur bones, including at least 70 individuals representing a minimum of nine genera (Madsen Jr, 1976; Gates, 2005). Of these, over 60% (Minimum Number of Individials (MNI): 46, based on a count of left femora) are attributable to a single taxon—*Allosaurus fragilis*, yielding a predator–prey ratio of 3:1, which is unusual compared to other herbivore-dominated Morrison Formation bonebeds (Madsen Jr, 1976; Miller, Horrocks & Madsen, 1996; Gates, 2005) (Fig. 2). Furthermore, over 85% of the *Allosaurus* remains are attributable to juvenile/subadult individuals (Madsen Jr, 1976, based on femoral measurements). Since the initial discovery of the site in 1927, nearly 10,000 bones have been collected by at least seven institutions. The first formal excavations were carried out by the University of Utah, collecting nearly 1,000 bones from 1929 to 1931 (Miller, Horrocks & Madsen, 1996). Excavations resumed again in 1939 through 1941 by WL Stokes and Princeton University, which excavated and collected approximately 450 bones during the three-year period. During the early 1960s, the University of Utah resumed excavations and collected nearly 7,000 bones from 1960–1964 (Miller, Horrocks & Madsen, 1996). Excavations resumed again in the late 1970s by the Utah Division of State History, and continued intermittently through the 1980s by Brigham Young University, collecting nearly 1,100 bones (Miller, Horrocks & Madsen, 1996). The quarry was once again worked from 2001–2003 through the Natural History Museum of Utah, yielding nearly 400 bones (Gates, 2005). In 2012, a coordinated effort between the University of Wisconsin-Oshkosh and Indiana University of Pennsylvania began surveying the quarry and began excavations in the south Butler Building, collecting nearly 50 bones to date.

While nearly all prior research conducted at the CLDQ has focused on macrovertebrate taxonomy and taphonomy (e.g., Dodson et al., 1980; Stokes, 1985; Hunt, 1986; Richmond & Morris, 1996; Gates, 2005; Hunt et al., 2006), geochemical considerations have received considerably less attention (i.e., Bilbey, 1999). Furthermore, other data types (such as microfossils) are available to aid in interpreting the depositional environment, yet have received only passing attention. Charophytes and ostracods have been recovered from the quarry matrix and utilized for general depositional interpretations (Suarez, 2003; Gates, 2005; Hunt et al., 2006). Although specimens of turtle shell and shed crocodilian teeth have been reported in the quarry, their abundance is low (i.e., two shed crocodilian teeth and a few fragments of turtle shell). This contrasts with other sedimentologically similar deposits of the Morrison Formation, suggesting that the depositional environment at the CLDQ may have been ephemeral or seasonal rather than more permanently inundated (Madsen Jr, 1976; Gates, 2005).

However, the Cleveland-Lloyd Dinosaur Quarry does contain abundant small bone fragments (<10 mm) within the lithified mudstone matrix (Gates, 2005). While far too small for taxonomic diagnosis, these intramatrix bone fragments (IBFs) can be characterized as potentially transportable sedimentary particles (“bioclasts”); the fragments are widely dispersed throughout the lithified matrix of the quarry, suggesting syndepositional incorporation of the fragments with the larger remains in the quarry assemblage. The bones from which these fragments are derived would have been stripped of soft tissue prior to breakdown at the surface and subsequent incorporation into CLDQ sediments. As such, patterns such as hydraulic equivalence and abrasion can be utilized to further interpret depositional histories (Peterson, Scherer & Huffman, 2011).

In addition to the microfossils and microvertebrate remains at the quarry, additional taphonomic data are available for reconstructing the depositional history of the CLDQ. Feeding traces are nearly absent on bones recovered from the quarry (Gates, 2005; Hunt et al., 2006). Furthermore, concretions of calcite with trace amounts of barite form as nodules (calcite/barite nodules hereafter) around many of the bones from the CLDQ (Bilbey, 1999). Finally, the bones in the quarry are found isolated or associated, and only rarely articulated (Gates, 2005).

Although recent researchers agree that the deposit was formed in a small, likely ephemeral, pond (e.g., Richmond & Morris, 1996; Gates, 2005; Hunt et al., 2006), the above evidence has led to a suite of highly variable explanations of the specific taphonomy and depositional environment preserved at the CLDQ. Preliminary interpretations classify the quarry as a drought-induced death assemblage (Stokes, 1945; Gates, 2005). Another hypothesis suggests that the deposit represents a predator trap (i.e. herbivores mired in the mud attracted numerous carnivores who also became mired) to explain the high numbers of *Allosaurus* (Richmond & Morris, 1996). Bilbey (1999) posits that the deposit represents a lethal spring-fed pond or seep where dinosaurs died after drinking the water. Hunt et al. (2006) suggests that the dinosaur remains at the quarry represent a single population that died from an unknown cause and were subsequently transported into a shallow pond.

Each of the above taphonomic hypotheses is insightful; however each hypothesis conflicts with the existing taphonomic data to some degree. For example, bones in a predator trap should be heavily tooth-marked, as are over 50% of the bones recovered from the Rancho La Brea tar pits (Spencer, Valkenburgh & Harris, 2003). However, only 4% of bones recovered from the CLDQ show evidence of feeding traces (Gates, 2005). Furthermore, bones from a predator trap would show a higher frequency of taphonomic modification, such as bones crushed by larger animals, such as sauropods, attempting to escape the miring mud, and post-depositional pit wear from remains wearing against each other during early diagenesis (i.e., Friscia et al., 2008). While approximately 30% of the bones collected from the CLDQ show evidence of crushing (Gates, 2005), pit wear is absent on the remains at the CLDQ.

Bilbey’s (1999) lethal spring fed pond hypothesis is powerful in that it can explain the lack of microvertebrate remains found at the CLDQ; a toxic pond would not support fish, turtles, and crocodilians typical of pond deposits. The spatial distribution of bones in the quarry also fits well with Bilbey’s (1999) hypothesis, and the ‘dinoturbation’ hypothesized to contribute to the disarticulation of remains would result in *in situ* bone crushing. However, organisms would likely move away from the pond after drinking the water, rather than remaining until death to be buried in place. Additionally, Bilbey (1999) does not explain the potential source of the toxicity of the pond.

Given the complicated taphonomy of the CLDQ and the uncertainty of the proposed taphonomic hypotheses, new data is required to create a hybrid hypothesis for the CLDQ that can address the complications of the available data. Here we present new taphonomic and geochemical data in the forms of x-ray diffraction (XRD) and x-ray fluorescence (XRF) data from a stratigraphic column spanning the Salt Wash and Brushy Basin members of the Morrison Formation, which crops out in the Cow Flats Quadrangle, including XRF data from sediment and bone fragments from the CLDQ (Figs. 1A, 1B). These data are contrasted with geochemical data from a lithologically similar Brushy Basin bonebed, the Mygatt-Moore Quarry (MMQ). We further provide characteristics of the intramatrix bone fragments (IBFs) of the CLDQ and a new locality (UWO-12-001, “Johnsonville”) in an attempt to formulate depositional and taphonomic inferences among sites with different taphofacies. The following questions are addressed:

1. What are the geochemical signatures of the CLDQ, and how do they compare to the regional Morrison Formation outcrops and bonebeds?
2. What are the abrasion patterns of recovered IBFs from the CLDQ?
3. What do the hydraulic equivalences of IBFs suggest about depositional history of the CLDQ? The geochemical and micropaleontological data presented enhance existing data available to better interpret the CLDQ taphonomy.

## Geologic Setting

The CLDQ and Johnsonville localities are located on the northern end of the San Rafael swell, southwest of Price, Utah, and stratigraphically located in the Brushy Basin Member of the Upper Jurassic Morrison Formation (∼147 Ma) (Bilbey, 1998). The Brushy Basin Member is composed of floodplain-deposited mudstones with freshwater limestones and some channel sandstones, and is the youngest of three laterally extensive members of the Morrison Formation (Gates, 2005). In the immediate vicinity of the CLDQ and Johnsonville sites are the distal alluvial fan complex of the Salt Wash Member, which underlies the Brushy Basin Member, and the Middle Jurassic Summerville Formation, which underlies the Morrison Formation (Peterson & Turner-Peterson, 1987). Previous reconstructions of Late Jurassic climate patterns in Utah indicate strong seasonality, subject to variably arid to monsoonal conditions (e.g., Hallam, 1993; Dodson et al., 1980; Rees, Ziegler & Valdes, 2000; Parrish, Peterson & Turner, 2004; Sellwood & Valdes, 2008; Tanner, Galli & Lucas, 2014). This interpretation is supported by scarce plant material and coal deposits (Dodson et al., 1980) and the distribution of authigenic minerals such as barite, present throughout the Morrison Formation that are strongly associated with periodic aridity (Turner & Fishman, 1991).

### Cleveland-Lloyd Dinosaur Quarry (CLDQ)

**Figure 3:**
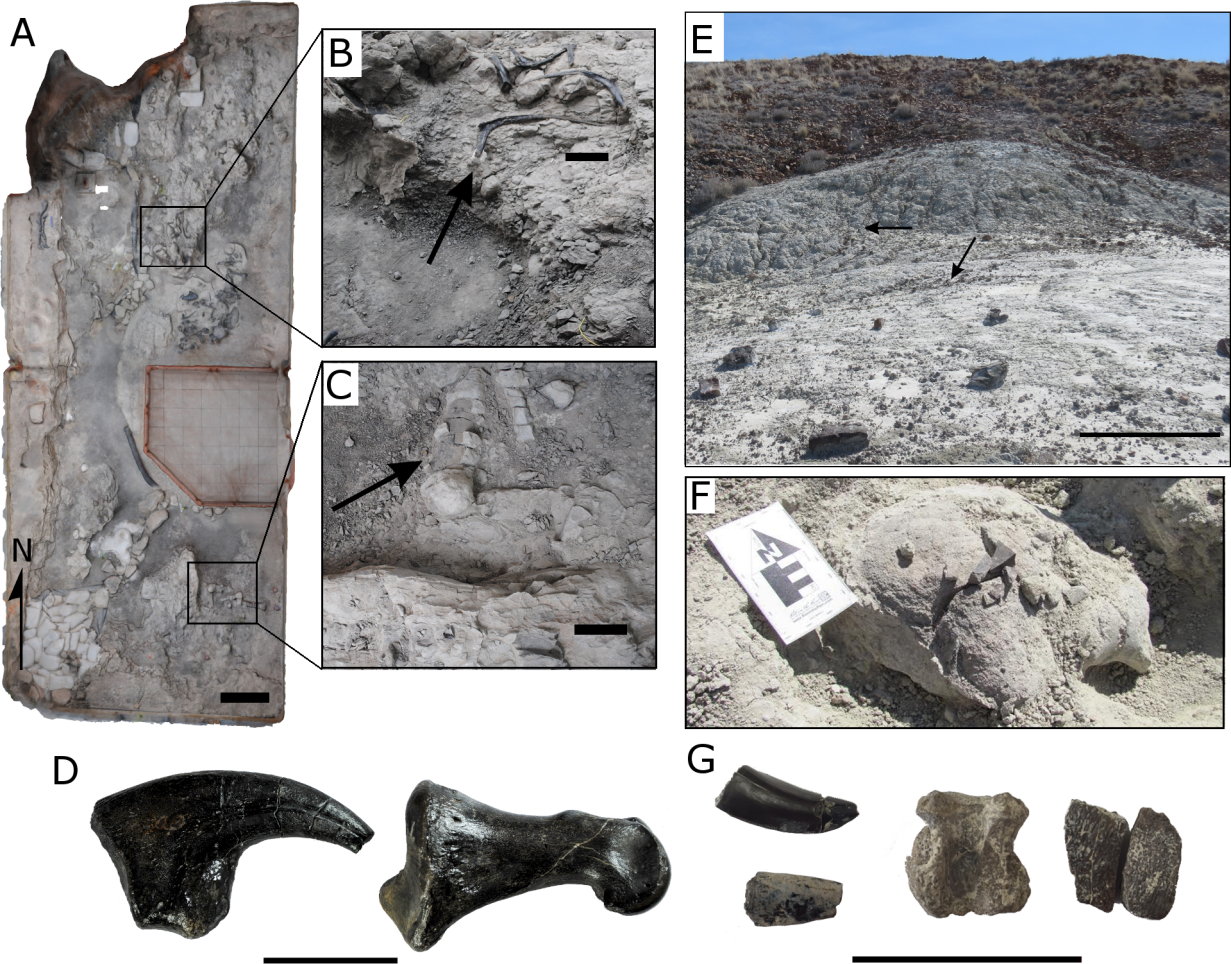
Fossils and characteristics of the Cleveland-Lloyd Dinosaur Quarry and the Johnsonville site. (A) A photogrammetric reconstruction of the North Butler building of the Cleveland-Lloyd Dinosaur Quarry (CLDQ), illustrating the locations from which sediment samples were taken for IBF and geochemical analyses. Scale bar equals 1 m; (B) Arrow annotating the location where approximately 30 kg of sediment was collected for analyses. Sediment was collected following the excavation of a series of theropod thoracic ribs. Scale bar equals 10 cm; (C) Arrow annotating the location where approximately 30 kg of sediment was collected for analyses. Sediment was collected following the excavation of a theropod femur and tibia. Scale bar equals 10 cm; (D) *Allosaurus* manual ungual (left) and *Allosaurus* pedal phalange (right) as examples of bone preservation from the CLDQ, Scale bar equals 5 cm; (E) Photograph of the Johnsonville (JONS) site, with arrows annotating the locations from which sediment samples were taken for IBF and geochemical analyses. Scale bar equals 1 m; (F) Sauropod caudal vertebra collected from JONS. Scale bar equals 10 cm; (B) Shed theropod teeth (left), crocodilian vertebra (center), and turtle shell (right) as examples of fossils commonly collected from JONS. Scale bar equals 5 cm.

The Cleveland-Lloyd Dinosaur Quarry (CLDQ) is located approximately 38 m above the basal contact of the Brushy Basin Member (Bilbey, 1992) (Figs. 1A–1B, 3A–3D). The bone-bearing unit is composed of a calcareous mudstone that varies in thickness from a few centimeters to one-meter, and also includes abundant clay clasts and diagenetic nodules. The calcareous mudstone underlies a bone-bearing micritic limestone unit that varies in thickness from 0.3–1.0 m, and overlies a massive silty mudstone approximately 20 m in thickness (Gates, 2005; Bilbey, 1992). Based on limited exposures, the silty mudstone unit is laterally continuous for 50–75 m before pinching out to the south (Gates, 2005). The calcareous bone-bearing mudstone contains calcite/barite nodules, typically as overgrowths on bone, interpreted as resulting from soft tissue decay (Bilbey, 1998; Gates, 2005). Via the removal of water and subsequent concentration of dissolved compounds towards saturation, the evaporative nature of the deposit would help to form these, however the nodules would have to form when the pond still contained water. While various depositional models have been proposed for the CLDQ, the lithologies, the abundant vertebrate macrofossils, and rare microvertebrate and invertebrate remains suggest an ephemeral pond or similar-overbank deposit with a fluctuating water table (calcareous mudstone facies) that became a more permanent basin in the form of a shallow lacustrine setting (limestone facies) (Bilbey, 1999; Gates, 2005). The presence of freshwater ostracods, gastropods and charophytes in the limestone cap over the bone-bearing mudstone suggests that the environment supported a freshwater ecosystem during the last stages of sediment filling the pond (Bilbey, 1992). The deposit has been dated to 147.2 ± 1 Ma to 146.8 ± 1 Ma via K/Ar dating of an ash bed approximately 1 m above the limestone cap (Bilbey, 1998).

### Johnsonville (UWO-12-001 “JONS”)

The Johnsonville quarry (University of Wisconsin Oshkosh locality UWO-12-001) is located 470 m southeast of the CLDQ locality and is stratigraphically positioned approximately 11 m below the CLDQ in the lower portion of the Brushy Basin Member (Figs. 1A, 1B). The site was discovered by the University of Wisconsin Oshkosh field crew in the summer of 2012. This site is composed of a 15-meter-thick yellowish/gray silty mudstone. The JONS site is dominantly a microsite; vertebrate microfossils, such as turtle shell fragments, shed crocodilian and theropod dinosaur teeth are common, but the site also includes several larger macrovertebrate remains such as a single sauropod caudal vertebra and other large weathered vertebrate bone fragments (Figs. 3E, 3G). Fossil material is present in the upper six meters of the silty mudstone. The Johnsonville unit is laterally extensive over ∼10 m and overlies a one-meter tan sandstone. Based on interpretations of similar lithologies in the Morrison Formation, the Johnsonville site is interpreted as an overbank deposit such as a wet floodplain or crevasse splay with a relatively high water table (Bilbey, 1999). While the JONS site is a microvertebrate locality, possessing fossils commonly associated with microsites (e.g., shed teeth, turtle shell fragments) and not a large bonebed like the CLDQ, it also contains small bone fragments (<10 mm) dispersed throughout the lithified matrix, and serves as a robust comparison to the CLDQ as a depositionally distinct site for taphofacies comparisons.

## Materials and Methods

Collection localities for this study are managed by the Bureau of Land Management (BLM) and all fossil material was collected under survey and excavation permits. Exact coordinates for these collection sites are on file with the BLM and the Natural History Museum of Utah, where all collected materials are maintained. Fieldwork for this study was conducted under BLM Permit #UT12-003E during the 2014 and 2015 field seasons.

### Stratigraphy and geochemistry

Utilizing a Jacob’s staff and Brunton compass-clinometer, a bed-by-bed stratigraphic column was generated for the Brushy Basin and Salt Wash Members of the Morrison Formation from its lower contact with the Middle Jurassic Summerville Formation to the uppermost horizon of the Morrison Formation preserved at the upper limit of the butte above and to the west of the CLDQ (Figs. 1A, 1B). Beds were identified in the field on the basis of color and lithological change. Some beds were divided into subunits based on changes in grain size and surface weathering. A hand sample of rock from beneath the upper weathered horizon was collected from the center of each bed or subunit. Hand samples were also collected from the center of the fossiliferous mudstone horizon of the North and South Butler Buildings at the CLDQ.

As a comparison to CLDQ, three samples of siltstone and one bone fragment from the Mygatt-Moore Quarry (MMQ) were included in this analysis. The MMQ is located approximately 2.5 km east of the Colorado-Utah state line in western Mesa County, Colorado and stratigraphically positioned in the middle to lower Brushy Basin member (Trujillo et al., 2014). MMQ is composed of a 1 meter-thick smectitic mudstone with interbedded silt-sized grains and clay balls. The mudstone is rich in carbonized plant and wood fragments (Trujillo et al., 2014). Since 1981, the quarry has yielded nearly 2,400 bones of at least six different taxa of Jurassic dinosaurs, with sauropods, such as *Apatosaurus*, *Camarasaurus*, and diplodocines (cf. *Diplodocus* or *Barosaurus*) constituting 50% of the total assemblage, and 30% represented by theropods such as *Allosaurus* and *Ceratosaurus* (Foster, 2007). Due to its similar lithology to the CLDQ and rich abundance of dinosaur remains, lithified matrix samples and bone fragments from the MMQ were included in this analysis for comparisons with the CLDQ.

All rock samples were hand ground via mortar and pestle for XRD and XRF analysis. Bone chips from the CLDQ and MMQ were also analyzed via XRF and petrographic thin section, however they were not ground prior to analysis. XRD was carried out at the University of Wisconsin Oshkosh Department of Geology utilizing a Rigaku D/Max-2000T X-ray diffractometer operating at 40 kV and 40 mA and utilizing a Cu Ka target to determine sample mineralogy. XRD data was subsequently analyzed using the Jade (v9.3; Materials Data, Inc., Livermore, CA, USA) software package. XRF for all stratigraphic samples and CLDQ samples was conducted at Beloit College in Beloit, Wisconsin with a Niton XL3t GOLDD+ handheld XRF analyzer in Test All Geo mode. XRF of material from MMQ was conducted at Indiana University of Pennsylvania using an Innov X Delta Professional handheld XRF analyzer in soil mode.

### Intramatrix bone fragments

To better understand the various taphonomic processes that influence the deposition, preservation, and recovery of IBFs at the CLDQ, the JONS site was chosen as a comparison sites for these analyses due to the different sedimentary facies among the two sites (e.g., Wilson, 2008; Peterson, Scherer & Huffman, 2011). The data collected at the CLDQ and the JONS site included local thickness of the sedimentary subunits and their lateral extent where possible.

In order to quantify intramatrix bone fragment abundance, approximately 60 kg of bulk lithified matrix was quarried from each of two localities; the CLDQ, and JONS (UWO-12-001,“Johnsonville”). Lithified matrix samples were quarried from 10 cm below the actively weathered surface (Fig. 3E) to avoid biases caused by ongoing erosion of fossil samples and were subsequently disaggregated under controlled laboratory conditions (following Peterson, Scherer & Huffman, 2011).

**Figure 4:**
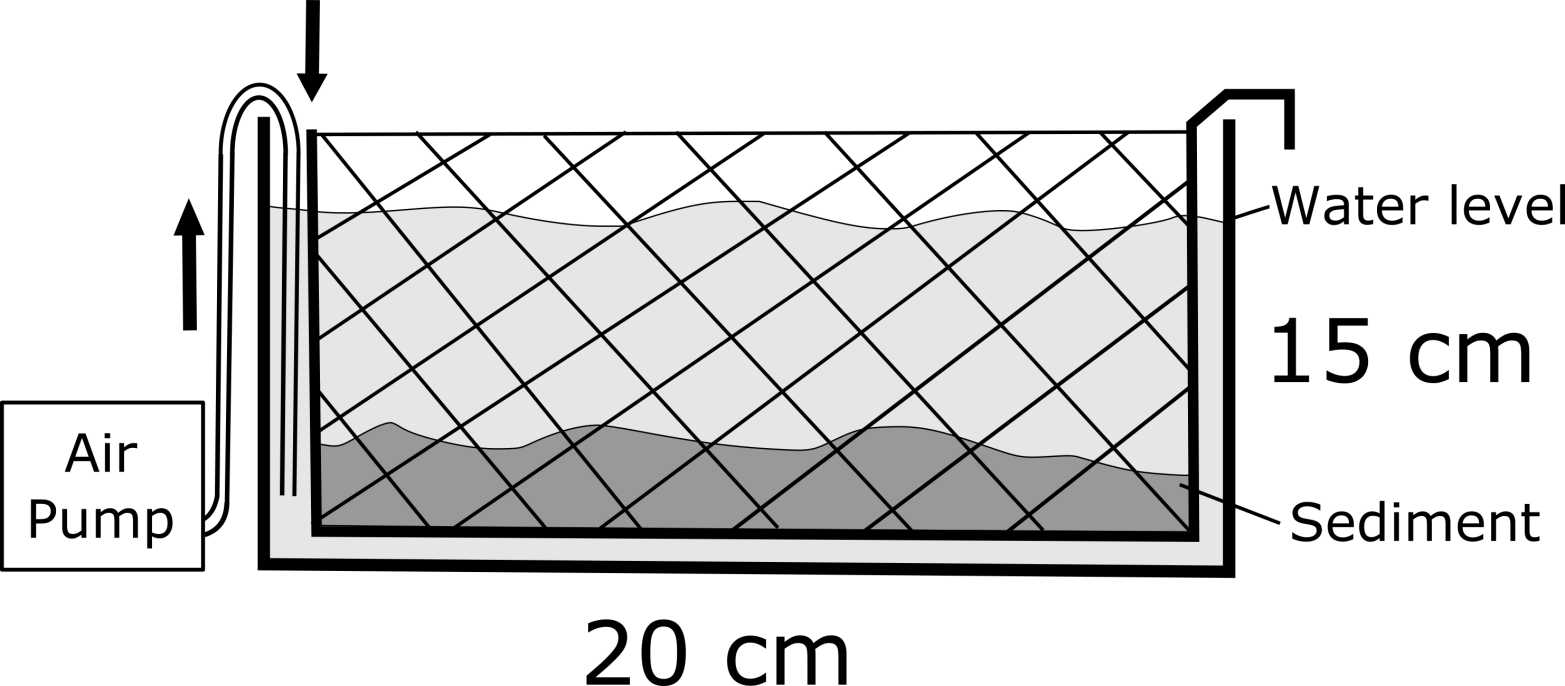
Schematic diagram of the sediment processing tanks. Sediment was placed into the meshed boxes and submerged with gentle air agitation for approximately 48–72 h.

All collected fossil fragments were obtained by a method of submerged screen washing with gentle air agitation similar to previously utilized methods (e.g., Mckenna, 1962; Ward, 1981; Peterson, Scherer & Huffman, 2011). Two mesh baskets 23 × 33 cm (23 cm deep) were constructed of 1 cm hardware cloth and internally lined with 1.0 mm plastic window screen. The mesh baskets were placed in 23 × 43 cm (28 cm deep) plastic basins. Below the mesh baskets, 1 meter of flexible perforated airline tubing was coiled at the bottom of the basins and connected to a double-output aquarium air pump (3.5 watt, 1,200cc air per minute output) placed outside of the basins. The resulting system produced gentle air-powered agitation in the basins to promote sediment disaggregation (Fig. 4). Unweathered lithified matrix samples were physically broken down to roughly 5 cm pieces, placed in the agitation basins, and submerged until disaggregation was complete, which took roughly two days. Following disaggregation, baskets were removed from the basins and left to air dry. A total of 1,155 fragments were collected from the 60 kg sample quarried from the CLDQ, and 616 fragments were collected from the 60 kg sample quarried from JONS.

Despite the inability to determine their precise taxonomic identity, all fossil bone fragments were collected and measured along three perpendicular axes to determine volume and hydraulic equivalence (Behrensmeyer, 1975). The equation for hydraulic equivalence is:

\begin{eqnarray*}\mathrm{dq}=(\mathrm{pb}-1)\times \mathrm{db}/1.65 \end{eqnarray*}

db = Nominal diameter of bone $=\sqrt[3]{}$ (1.91 × Volume)

pb = Bone density.

**Figure 5:**
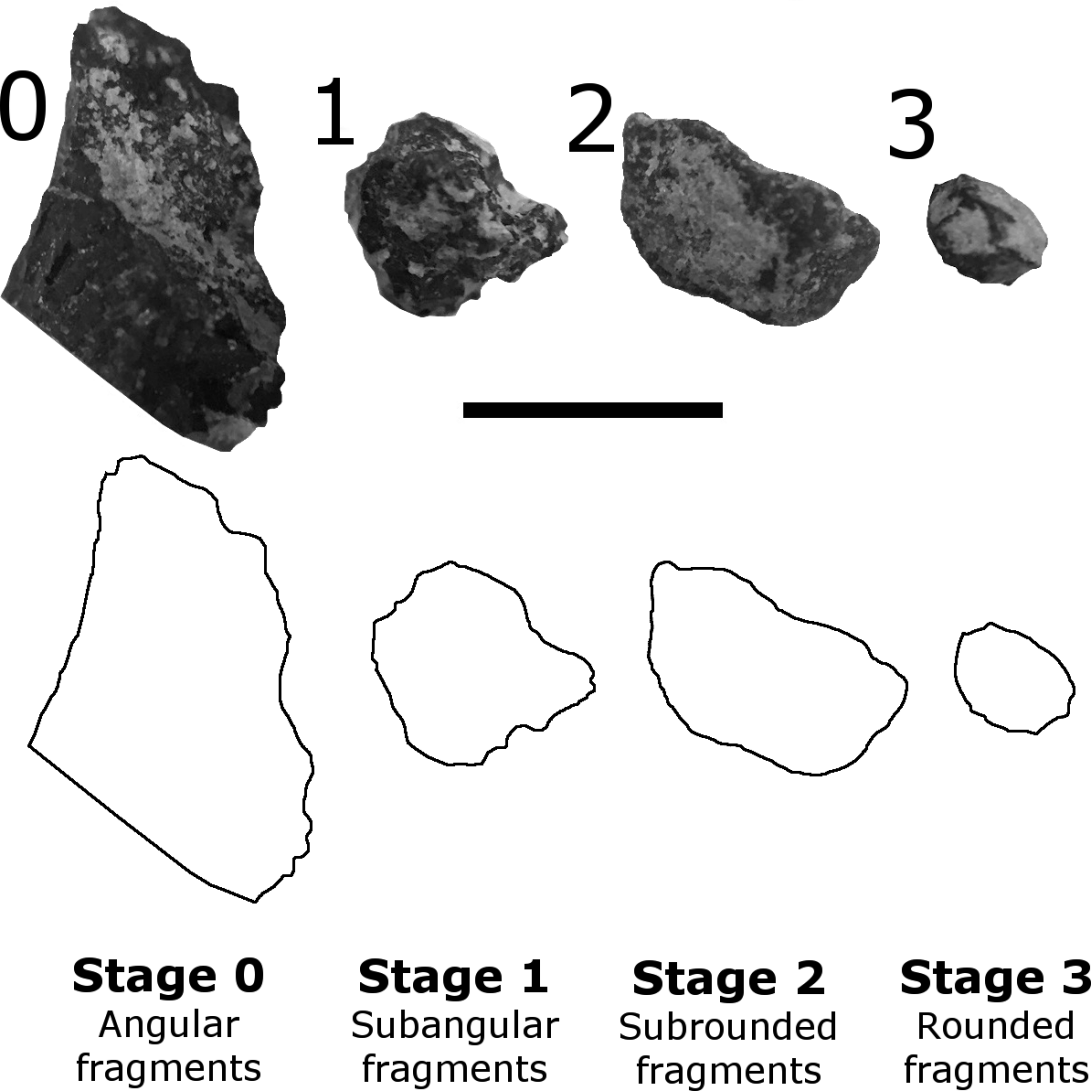
Examples of fragment abrasion stages, based on Peterson, Scherer & Huffman (2011). Stage 0—angular fragments; Stage 1—subangular fragments; Stage 2—subrounded fragments; Stage 3—rounded fragments. Scale bar equals 5 mm. All fragments imaged were collected from CLDQ.

Fragments were also classified by their relative degree of abrasion as a signal of relative exposure time prior to burial; the less-angular fragments representing longer periods of exposure and/or re-working than more-angular fragments. Based on the observed collection of fragments, abrasion was measured on a 0–3 scale (modified from Peterson, Scherer & Huffman, 2011), with 0 representing angular fragments with no apparent abrasion, and 3 representing relatively more rounded fragments with fewer sharp angular edges on the specimen (Fig. 5).

Chi-square tests were used to determine whether a statistically significant relationship existed between two categorical variables. A nominal level of significance (type I error rate) of *α* = 0.05 was used in all tests, i.e., an observed significance level (*p*-value) of <0.05 was required in all tests to reject the null hypothesis that the variables were not related. Specifically, chi-square tests were used to determine if the degree of abrasion of the intramatrix bone fragments significantly differed between the two sites.

## Results

### Geochemistry

XRD data are presented in Tables 1A and 2B. Excluding the CLDQ, the analyzed outcrops of the Morrison Formation are overwhelmingly composed of silicates and carbonates. Two analyzed strata, Units 1b and 25, also contained dolomite and ankerite (CaMg_0.27_Fe_0.63_(CO_3_)_2_) (Table 1A). The CLDQ itself is composed primarily of silicates and carbonates, but distinguished by the presence of barite, chalcopyrite, fluorapatite, covellite (CuS) and litharge (PbO). XRD analysis of sediments from the JONS site detected only silicates.

**Table 1:** XRD data. All minerals identified in every sample analyzed from the stratigraphic column are presented. (A) XRD data from stratigraphic column; (B) XRD data for CLDQ samples. CLDQ stands out given the presence of barite and sulfide minerals.

CLDQ regional strat samples	Identified peaks
A	
Unit 01 sub1	calcite
Unit 01 sub2	graphite; quartz; dolomite; ankerite
Unit 02	calcite
Unit 03	calcite
Unit 04	quartz
Unit 05	calcite
Unit 06	quartz
Unit 07	quartz
Unit 08	quartz
Unit 09	quartz
Unit 10	quartz
Unit 11	quartz
Unit 12	quartz
Unit 13	calcite; quartz
Unit 14	quartz; calcite
Unit 15	quartz; calcite
Unit 16	quartz
Unit 16 sub1	quartz; calcite
Unit 17a	quartz; calcite
Unit 17b	quartz
Unit 18a	quartz
Unit 18b	quartz; calcite
Unit 19	Quartz
Unit 20	calcite; quartz
Unit 21a	quartz
Unit 22 - Johnsonville	quartz
Unit 23	quartz
Unit 24	quartz
Unit 25	dolomite; ankerite; quartz; graphite
Unit 26	calcite; quartz
Unit 27	quartz
Unit 28	quartz
Unit 29	quartz
Unit 30	quartz
Unit 31	quartz
Unit 32	quartz
Unit 33	quartz
Unit 34	quartz
Unit 34 sub1a	quartz
Unit 34 sub1b	quartz
Unit 35	quartz
Unit 36	quartz; graphite
Unit 37	quartz
Unit 37 sub1	calcite; quartz

CLDQ quarry samples	Identified peaks
B	
Sample 1	quartz; qalcite
Sample 2	quartz
Sample 3	quartz; calcite
Sample 4	quartz; calcite
Sample 5	calcite; quartz
Sample 6	calcite; quartz; barite; chalcopyrite; fluorapatite; litharge
Sample 7	fluorapatite; calcite
CLN BF1	calcite; fluorapatite; chalcopyrite; covellite
SBF1	fluorapatite; calcite

**Table 2:** Taphonomic characteristics and comparisons of intramatrix bone fragments of the CLDQ and JONS sites. Characteristics include (A) dominant lithology, (B) depositional interpretation, (C) percentages of 60 kg matrix sample represented by fossil fragments, (D) number of IBFs assigned to each abrasion stages on Peterson, Scherer & Huffman (2011), and (E) hydraulic equivalence calculation results. Hydraulic equivalence values are measured in mm, and relative densities are calculated in grams/cm3.

		CLDQ (*n* = 1,155)	JONS (*n* = 616)
**A**	**Dominant lithology**	Calcareous mudstone	Silty mudstone
**B**	**Inferred depositional environment**	Ephemeral pond (Gates, 2005)	Wet floodplain/crevasse splay
**C**	**% Fossil Fragments per 60 kg Sample**	0.08%	0.04%
**D**	**Abrasion**		
	Stage 0	213	34
	Stage 1	347	178
	Stage 2	311	246
	Stage 3	284	158
**E**	**Hydraulic Equivalence**		
	Average relative densities (g/cm^3^)	0.124	0.133
	Average hydraulic equivalent (mm)	0.08	0.09
	Equivalent grain size	Fine sand	Fine sand

**Figure 6:**
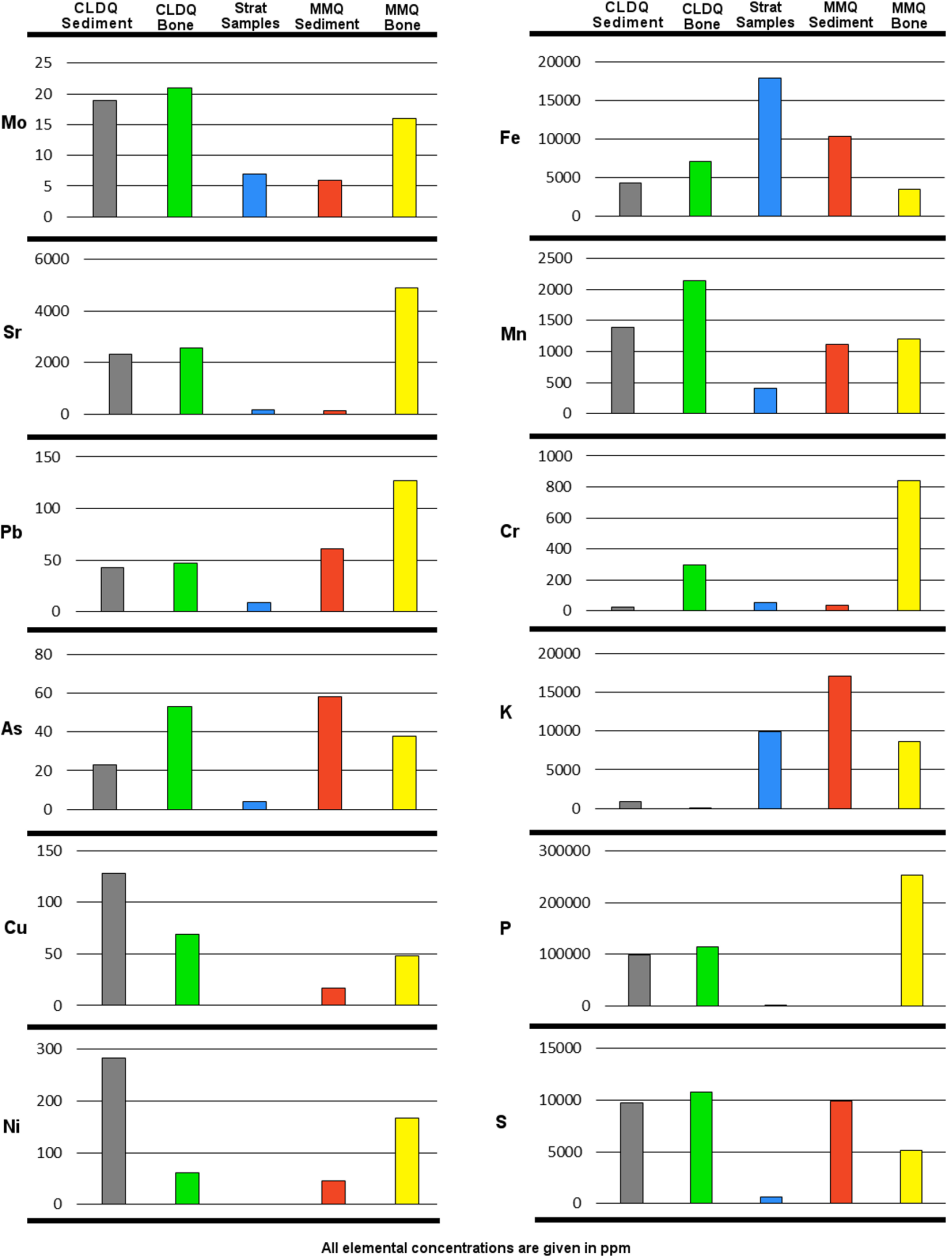
X-ray fluorescence results. The concentrations of selected metals detected in sediment (gray) and bone (green) collected from the CLDQ are compared with those of sediments from local Morrison Formation strata, labeled “Strat Samples” (blue) and sediment and bone from MMQ (red and yellow, respectively). CLDQ sediments and bone stand out in contrast to most other elements, yet are similar to those found at MMQ. All values are given in ppm.

The total XRF data are presented in Table S1. Comparisons of the concentrations of selected metals in CLDQ bone and sediment, MMQ bone and sediment, as well as regional Morrison Formation sediment is presented in Fig. 6. The XRF unit utilized to analyze MMQ samples does not detect Si. Si concentrations, available for all non-MMQ samples, ranged between 24,646.01 ± 581.59 ppm to 522,405.2 ± 4166.98 ppm throughout the sampled units of the Salt Wash and Brushy Basin Members in the vicinity of the CLDQ, with samples from the quarry having values of 24,646.01 ± 581.59 ppm and 30,643.28 ± 1,309.51 ppm. The ‘balance’ measured by the XRF ranged from 282,284.22 ± 4,650.26 ppm to 712,203.63 ± 2,258.34 ppm, including the summed concentration of all elements lighter than Mg (those not detectable by the unit), and occasional instrument error due to air space in the ground samples. The quarry samples yielded balance values of 412,387.59 ± 4,891.71 ppm and 436,363.78 ± 3,107.65 ppm. Sediment samples from the CLDQ bonebed had higher values than those of the rest of the stratigraphic column for Mo, Sr, As, U, Cu, Ni, Nb, P, and S. The CLDQ bonebed sediment had higher concentrations than most other samples from the stratigraphic column for Pb, Mn and Cr. The quarry sediment fell within the range the rest of the stratigraphic column for Zr, Rb, Th, Zn, W, Si, V, Ti, Ca, K, Al, Cl, Sc, Mg and Fe. Any elements not specifically listed above were either not present or undetectable via the XRF gun in all analyzed samples. Sediments from Mygatt-Moore Quarry contained more Cu, Ni and Bi than the stratigraphic column samples. MMQ sediment contained more Rb, Pb, As, Zn, Cl and V than most stratigraphic samples. Finally, MMQ samples were within the range of concentrations of Mo, Zr, Fe, Mn, Ti, Ca, K, Cl, Sr, W, Cr, and P seen within the stratigraphic samples. In contrast to the CLDQ and MMQ, the sediments of JONS resemble those of local Morrison Formation outcrops. None of the metals detected via XRF at JONS were found at elevated levels compared to all other samples analyzed, including those of the CLDQ and MMQ.

**Figure 7:**
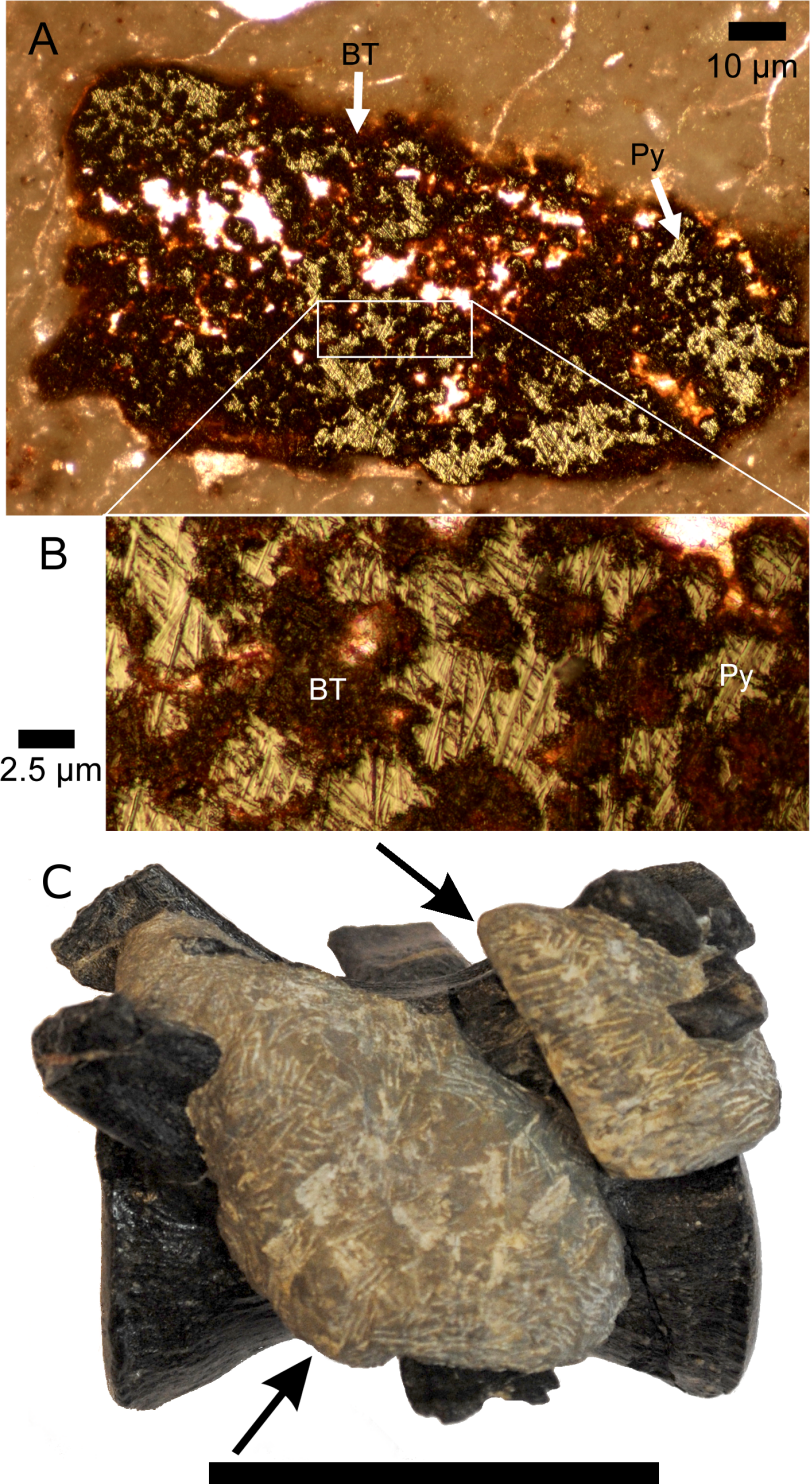
Diagenetic alteration to bones and bone fragments at CLDQ. (A) Intramatrix bone fragment from CLDQ in petrographic thin section. Arrows annotating the location of bone tissue (BT) and pyrite crystals (Py) in porous cavities within the fragment at 5× magnification (scale bar equals 10 µm), and (B) at 20× magnification (scale bar equals 2.5 µm). (C) Allosaurus caudal vertebra (UMNH.A.2012.26.020) collection from CLDQ possessing barite growth across articular processes. Arrows annotating the presence of diagenetic nodules adhered to the surface of the bone. Scale bar equals 5 cm.

CLDQ bone revealed a similar pattern of heavy metal enrichment as CLDQ sediments (Fig. 6). Exceptions include As and Cr, for which the bone samples have similar concentrations to Morrison Formation sediment as opposed to CLDQ sediment, i.e., CLDQ sediments contain more abundant As and Cr than both Morrison Formation sediment and CLDQ bone. CLDQ bone is more enriched in Ni, Cu and W with respect to Morrison sediment than CLDQ sediment. For all other detected elements, the bone samples and sediment samples from the CLDQ show similar elemental concentrations. Thin section analysis of bone fragments encased in quarry matrix from CLDQ indicate permineralization in the form of pyrite crystals filling void spaces within the bone fragments (Figs. 7A and 7B). Previous analyses of whole bone from CLDQ (e.g., Bilbey, 1999), revealed quartz infilling of pore spaces, in contrast with the pyrite found within IBFs here.

In regards to MMQ, the bone sample generally had similar heavy metal content to that of the sediment samples. Exceptions include Sr, W, Cr, V and P that were in higher concentrations in the bone than the sediments and K that was in higher concentration in the sediment than in the bone.

### Intramatrix bone fragments

**Figure 8:**
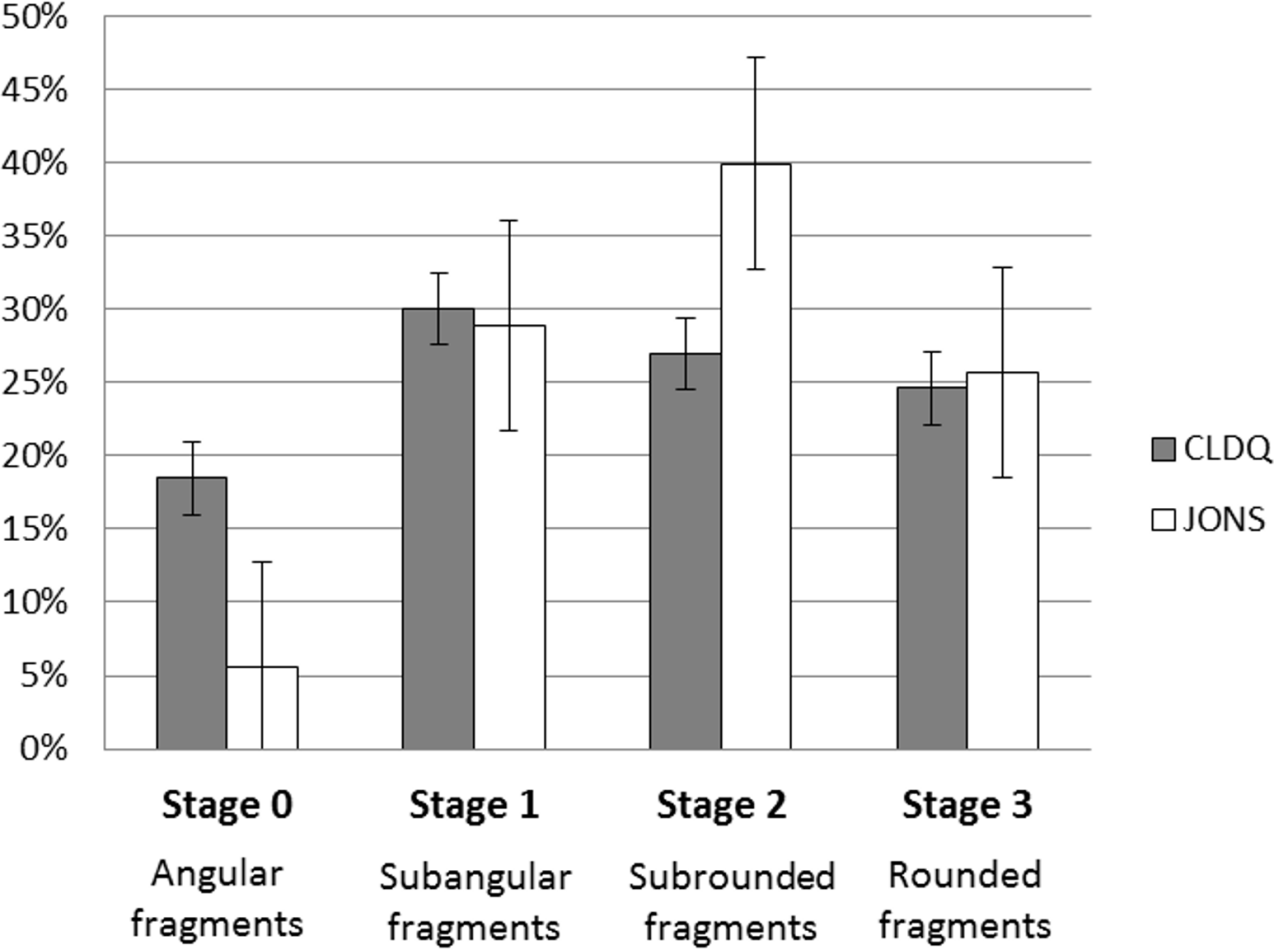
Distribution of abrasion stages of intramatrix bone fragments at the CLDQ (gray) and JONS (white) localities. Error bars represent standard error.

The IBFs collected from CLDQ and JONS were compared according to their physical characteristics and taphonomic differences to investigate whether these differences were statistically significant (raw data available in Tables S2 and S3). The analyses suggest different patterns of abrasion variability between the two localities, and these patterns were found to be significantly different (*p* < 0.001; Tables 2A–2D, Fig. 8).

The CLDQ IBFs display a wide range of degrees of abrasion; each abrasion category contained 15–30% of the recovered IBFs (Fig. 8). However, the JONS IBFs possess higher degrees of abrasion; JONS bone fragments show a strong trend towards more mature levels of abrasion, with only 5% of IBFs scoring as angular.

Following Behrensmeyer (1975), hydraulic equivalents (E_*HY*_) were calculated based on specimen volume, relative density, and size for each assemblage of bone fragments. Hydraulic equivalences and relative densities were compared between assemblages (Table 2E). Although shape is a significant factor for settling velocities, this feature has been omitted simply to gain a general view of hydraulic equivalences to a quartz sphere (Behrensmeyer, 1975; Peterson, Scherer & Huffman, 2011). Results of hydraulic equivalence analysis indicate that bone fragments obtained from both localities have similar hydraulic equivalences (fine sand) despite differences in site lithology.

## Discussion

### Geochemistry

X-ray diffraction data largely agree with those presented by Bilbey (1999), in that the Brushy Basin and Salt Wash Members of the Morrison Formation are primarily composed of quartz and calcite. The mineralogy of the CLDQ itself is distinguished from the surrounding Brushy Basin and nearby Salt Wash Members, as well as the mudstones at the same stratigraphic level as the CLDQ, by the presence of metal oxides (primarily litharge), sulfides (chalcopyrite and covellite) and barite. Furthermore, sulfide minerals (i.e., pyrite crystals) were found within IBFs from CLDQ (Figs. 7A, and 7B). This suite of minerals implies that the environment represented by the quarry was very likely reducing (Eby, 2004). High levels of decaying organic matter, i.e., an abundance of decaying dinosaurs, would utilize the available dissolved oxygen resulting in a reducing environment, especially given the ephemeral nature of the CLDQ pond. As discussed below, decaying dinosaurs may have been the source of the metals (Cu and Pb) needed to form metal oxides and sulfides.

The presence of calcite/barite nodules on the bones (Fig. 7C) further supports the hypothesis that the dinosaur remains were decaying in the CLDQ pond. Ligament and cartilage would be among the last of the organic matter to decay, and the highest concentration of dissolved organic matter would occur surrounding these tissues during late stage decay (Madsen Jr, 1976; Bilbey, 1999). The position of the calcite/barite nodules, most commonly found where ligaments and cartilage attached to bone (Madsen Jr, 1976), supports the hypothesis that they formed during decay. Furthermore, in subaqueous settings barite formation is associated with supersaturation of chemical microenvironments surrounding decaying organic matter (Paytan & Griffith, 2007).

Despite suggestions of an abundance of available organic matter at the CLDQ, evidence of scavenging is conspicuously rare, as previous authors have noted (e.g., Berner, 1968; Bilbey, 1998; Gates, 2005). However, hypereutrophic conditions due to the decomposition of dinosaur carcasses in the CLDQ deposit may also explain the rarity of scavenging traces on CLDQ bones. The presence of the calcite/barite nodules (Berner, 1968; Canfield & Raiswell, 1991) and sulfides (Wetzel, 2001) at the CLDQ suggests hypereutrophic freshwater conditions, which are commonly low diversity environments, especially with respect to vertebrates that would leave scavenging traces on bones (e.g., Heino, Virkkala & Toivonen, 2009). Hypereutrophic environments that have been recently disturbed, i.e., the ephemeral pond CLDQ likely represents, are particularly susceptible to low diversity (Moss et al., 2009).

X-ray fluorescence and x-ray diffraction data for the CLDQ show the quarry as enriched in heavy metals, e.g., Mo, As, U, Pb, relative to the rest of the local outcropping of the Morrison Formation. Some of these metals, Sr, Zn, Na and Mg, are known to easily replace Ca in biogenic apatite during diagenetic/post-depositional processes, such as interactions with groundwater (Trueman & Tuross, 2002; Goodwin et al., 2007). Furthermore, Morrison Formation bones are known to be enriched in U as a result of post-depositional and diagenetic processes, therefore elevated levels of U detected in CLDQ sediment and bones are not anomalous (Hubert et al., 1996). However, Gillette (1994) notes that U enrichment in dinosaur bone is most common in bones buried within the local water table. High U concentrations seen here help to support the hypothesis that the bones of CLDQ were deposited in an environment with a high water table, such as the shallow pond that CLDQ is suggested to represent (Gates, 2005).

Unfortunately, whereas studies of the geochemical compositions of fossil remains from bonebeds are common (e.g., Trueman & Benton, 1997; Trueman & Tuross, 2002; Rogers, Eberth & Fiorillo, 2010), similar studies focusing on the sediments from bonebeds are lacking in the literature. One possible origin for the heavy metals at CLDQ is accumulation through diagenetic processes. The high abundance of buried bone undergoing diagenetic dissolution may be responsible for the elevated levels of As. Elevated levels of As, Sr, Ce, Pb, and U have been noted in *Dilophosaurus* bones from the lower Jurassic Kayenta Formation, which is rich in iron oxides that can mobilize these metals, leading to the enrichment of bone material after burial (Goodwin et al., 2007). While this is a possible explanation for the origin of the metals detected at the CLDQ, it is worth noting, however, that even though Goodwin et al. (2007) found higher concentrations of As in bone than in the surrounding sediment (200–500 ppm in bone, 10–20 ppm in sediment), the opposite was seen at CLDQ (18–28 ppm in bone, 50 ppm in sediment). Two of the three sediment samples from MMQ contained more As than the bone from MMQ, following the pattern observed by Goodwin et al. (2007). Strong negative pair wise elemental correlations were observed for As and Fe in the CLDQ sediment samples, similar to what has been observed in material from the Kayenta Formation (Goodwin et al., 2007). This may reflect As desorption from iron oxides, given that Fe is low at the CLDQ and higher elsewhere in the Morrison Formation; the negative correlation between Fe and As at the CLDQ may be the result of desorption and absorption reactions during the dissolution and weathering of the large accumulation of bones. Bone dissolution would produce a high concentration of P, as observed via XRF, which can promote desorption of As (Goodwin et al., 2007). It is possible that similar processes resulted in the high concentrations of other metals in the sediments of CLDQ relative to concentrations seen in the rest of the Morrison Formation analyzed here, however these elements were not discussed by Goodwin et al. (2007). Elevated concentrations of metals seen in MMQ bone support the conclusion that diagenetic processes contributed to the heavy metal signature observed at CLDQ.

A second possibility for the origin of the elevated heavy metals at the CLDQ is bioaccumulation. Studies of modern grave soils suggest one potential source of heavy metals detected in bone and sediment of the CLDQ that are not necessarily explained by apatite diagenesis, i.e., Ni, Cu, Mo, As, Pb and W: the dinosaur carcasses themselves. Modern grave soils have long been seen as potential ecological hazards and sources of organic and inorganic pollutants (Aruomero & Afolabi, 2014). Whereas many studies of necrosols focus on burials with caskets which are not relevant to a Mesozoic bonebed (e.g., Ücisik & Rushbrook, 1998), some studies have focused on geochemistry of mass graves and primitive burials lacking caskets and burial goods (Kemerich et al., 2012; Amuno, 2013). Kemerich et al. (2012) utilized XRF to find elevated levels of Ba, Cu, Cr and Zn in the soil and groundwater associated with a mass grave in Brazil. Amuno (2013) found elevated levels of As, Cu, Cr, Pb and Zn in necrosols within and near to a mass grave site in Rwanda. Despite full soil development not being evident at the CLDQ, the deposit is an analogous accumulation of quickly buried vertebrate remains in fine grained sediment.

Even though it is highly unlikely that high concentrations of As, Cu, and Pb seen in CLDQ sediments indicate these metals occurred at toxic concentrations in the bodies of the dinosaurs which accumulated there (Goodwin et al., 2007), large numbers of carnivores decaying could lead to the accumulation of these metals in the CLDQ pond. Carnivores are especially likely to contribute heavy metals via trophic focusing of toxins as they are high-level consumers (e.g., Vijver et al., 2004; Gall, Boyd & Rajakaruna, 2015). Even though more extensive work is required to interpret the geochemical signal of the bones recovered from the CLDQ, the similarity of CLDQ sediment and preliminary bone geochemistry data, taken with the strong contrast between geochemistry of sediments from the CLDQ and from surrounding Morrison Formation sediments, implies a unique setting for the CLDQ assemblage.

The MMQ samples provide a significant comparison when considering bioaccumulation as a source of the metals observed at the CLDQ. Some elements found in CLDQ sediments and bone, specifically W, Cu, Ni and Cl, are found in higher concentrations in CLDQ materials than MMQ materials. However, Zr, Rb, V and K are found in higher concentrations at MMQ than at CLDQ.

Another possibility for the elevated presence of heavy metals found at the CLDQ is the dissolution of volcanic ash, which may have concentrated in the pond as it washed in during flood periods. Hubert et al. (1996) analyzed the chemical composition of “a large data base for silicic obsidians that proxies for the unknown composition of the altered silicic ashes in the Brushy Basin Member (page 537)”. The compositional data presented by Hubert et al. (1996) contrast significantly with the sediments of the CLDQ (Table 3). Given that the CLDQ sediment geochemistry does not match well with that of the proxy obsidians (Hubert et al., 1996), the metals present in the CLDQ are not likely sourced from local volcanic ashes emplaced during bone burial.

**Table 3:** Obsidian chemistry vs CLDQ chemistry. This table shows the contrasting geochemistry of metals detected in CLDQ sediments with that of obsidians which act as proxies for ashes emplaced locally during the Jurassic (Hubert et al., 1996). The contrasting elemental profiles suggest that volcanic ashes are not the source of metals detected at the CLDQ. Values are given in ppm.

Element	CLDQ	Obsidian
Cr	258–332	1–25
Ni	60–63	1–35
Pb	31–62	10–44
V	0	15–38
Rb	7–9	23–300

Finally, the heavy metals present at CLDQ could be a result of past mining activities. This idea is unlikely for several reasons, however. First, the active quarry at CLDQ is covered by the North and South Butler buildings. Any metal-rich dust carried from mining operations would be more likely to settle on surfaces outside of the quarry which are exposed to air. Furthermore, the dense limestone cap over the bone-bearing layer at CLDQ makes it unlikely that the metals would have been transported vertically onto the bone-bearing layer as dust or in solution. Given that the analyzed sediment was exposed after the Butler buildings were constructed, the metals are not likely sourced from mining activities prior to the Butler buildings’ construction. Finally, given that metals are seen in high abundance in both the CLDQ and MMQ bonebeds, but not in any of the other 44 samples of Morrison Formation sediment analyzed, it is most likely that the metals are related to the presence of fossil bone, not recent mining activities.

Both XRD and XRF analyses, taken together, support the hypothesis that the CLDQ represents an ephemeral pond that became hypereutrophic as dinosaur carcasses decayed. The source of the calcite/barite nodules on the bones and sulfide minerals present in the quarry, but not found in other local Morrison sediments, appears to derive at least in part from the decay and dissolution of the dinosaurs themselves. Dinosaur decay could potentially have contributed to the heavy metal signature of CLDQ sediments as well.

Hypereutrophy can explain the near total lack of microvertebrate remains, (turtle, fish and crocodilian fossils typically associated with pond deposits), and near total lack of scavenging marks on CLDQ dinosaur bones. The typical freshwater fauna that would create microvertebrate remains and also scavenge on the carcasses (fish, turtles and crocodilians), would not have been able to tolerate hypereutrophic water conditions. Furthermore, as the carcasses rotted, the formation of calcareous soaps may have deterred extensive scavenging before leading to the formation of calcite/barite nodules on bones. While both diagenetic processes and a hypereutrophic water column are possible sources of the heavy metals found at the CLDQ, given the evidence in support of hypereutrophy of the CLDQ pond, diagenetic processes are not likely the primary cause of elevated concentrations for all of the metals seen here. Furthermore, if the accumulation of heavy metals seen at CLDQ is a result of apatite diagenesis associated with the large number of bones at the site, the site-specific taphonomy of the CLDQ could have contributed to the heavy metal signature, as metals which had bioaccumulated in a large number of top consumers remained in the CLDQ pond during decay and lithification. Geochemical analysis of the JONS site strengthens the interpretation of the CLDQ as a unique bone-bearing site within the Morrison Formation. Typical indicators of eutrophy (elevated levels of metals, sulfide minerals and calcite/barite nodules) are not present at JONS at the levels observed at the CLDQ. Furthermore, a typical freshwater microvertebrate assemblage of turtle and crocodilian remains are found at JONS. These data support the hypothesis that diagenesis is not the sole contributor of heavy metals at the CLDQ. JONS also contains large vertebrate bones, however the heavy metal signatures seen at the CLDQ are absent there. If organic remains are the primary source for metals, concentrations would not be expected to be as high at JONS as at the CLDQ, given the disparity in number of fossils found at each site. However, if post-burial diagenetic processes were the dominant source of heavy metals in the sediments of the CLDQ, some elevation in these metals would be expected at JONS.

Finally, analysis of MMQ sediment and bone provides meaningful contrast to that found at CLDQ and again highlights the uniqueness of CLDQ. The bones of MMQ show extensive evidence of biostratinomic alteration, implying the presence of scavengers. Although aquatic vertebrate remains are rare at MMQ, as at CLDQ owing to the ephemeral nature of the pond (Trujillo et al., 2014), they are more numerous at MMQ. Furthermore, the sediments of MMQ contain abundant carbonized plant fragments whereas the mudstone layer of CLDQ contains none. This is potentially due to differences in local vegetation during the time of deposition, but could also be a taphonomic effect. Bones recovered from MMQ are not associated with calcite/barite nodules. Rates of organic matter decay at MMQ must not have been high enough to form the calcareous soaps necessary for calcite formation. A second possibility is that freshwater input to the MMQ pond was high enough to flush the system, inhibiting the formation of such soaps. Taken together, these data imply that the MMQ pond was not hypereutrophic, whereas the CLDQ pond was. The differences in preservation of bone and plant material, the respective presence and absence of calcite/barite nodules, as well as differences in biostratinomy and microvertebrate fossil abundance between the two sites are best explained by variations in water chemistry: periodic hypereutrophic conditions at CLDQ, and an oligotrophic pond at MMQ.

### Intramatrix bone fragments

Previous studies have discussed distinct taphonomic characteristics among microvertebrate fossils from localities with dissimilar facies (e.g., Behrensmeyer, 1975; Brinkman, 1990; Eberth, 1990; Blob & Fiorillo, 1996; Wilson, 2008; Peterson, Scherer & Huffman, 2011). Peterson, Scherer & Huffman (2011) reported on taphonomic variability of microvertebrate assemblages collected from crevasse-splay and flood basin deposits from the Late Cretaceous Hell Creek Formation of Carter County, Montana. Their results suggested a strong correlation between taphonomic processes such as transport, sorting, and weathering, abrasion/rounding, sedimentary facies, and physical characteristics of recovered fossils. A similar trend is observed in the collected intramatrix bone fragments from the CLDQ and the JONS site in the Upper Morrison Formation. Although both localities possess abundant bone fragments that share a suite of physical characteristics (i.e., sizes <0.5 mm and comparable relative densities), the variation in rounding/abrasion between the two sites is significant (*α* = 0.05).

The average hydraulic equivalence for both localities corresponded to fine sand (Table 2E). This similarity may be due to the specific measurement method utilized; minor differences in volume between angular and non-angular fragments may have been missed by standard angular measurements. However, given the small sizes and homogeneous densities of the fragments, the hydraulic equivalences of the assemblages are not expected to vary considerably with alternative measuring methods.

The matrices of both localities are dominated by sediments much finer than the respective hydraulic equivalence of bone fragments recovered from each site; the CLDQ is composed of a calcareous mudstone whereas the JONS site is dominated by a silty mudstone, while the bone fragments from both sites are hydraulically equivalent to fine sand. This disparity between the hydraulic equivalence of bone fragments and the dominant lithologies suggest that the intramatrix bone fragments at both localities are likely a mixture of autochthonous or parautochthonous fragments derived from locally-crushed or weathered larger bones and washed-in allochthonous fragments, all of which accumulated on the flood basin (CLDQ) or in a crevasse-splay (JONS) (Behrensmeyer, 1975; Wilson, 2008; Peterson, Scherer & Huffman, 2011).

The significant variability in the extent of abrasion between the sites is explained by the contrasting depositional conditions of the two localities. The JONS assemblage includes IBFs that are more rounded than the fragments found in the CLDQ assemblage. This suggests that the Johnsonville locality was subjected to relatively higher and more consistent energy. Although the sedimentological evidence does not indicate an in-channel depositional subsystem, the silty mudstone lithology and the presence of abundant freshwater microvertebrate fossils (i.e., crocodilian, turtle, and fish) support the interpretation of deposition occurring by a crevasse-splay in an environment with a generally higher water table, likely a wet floodplain (Bilbey, 1998).

**Figure 9:**
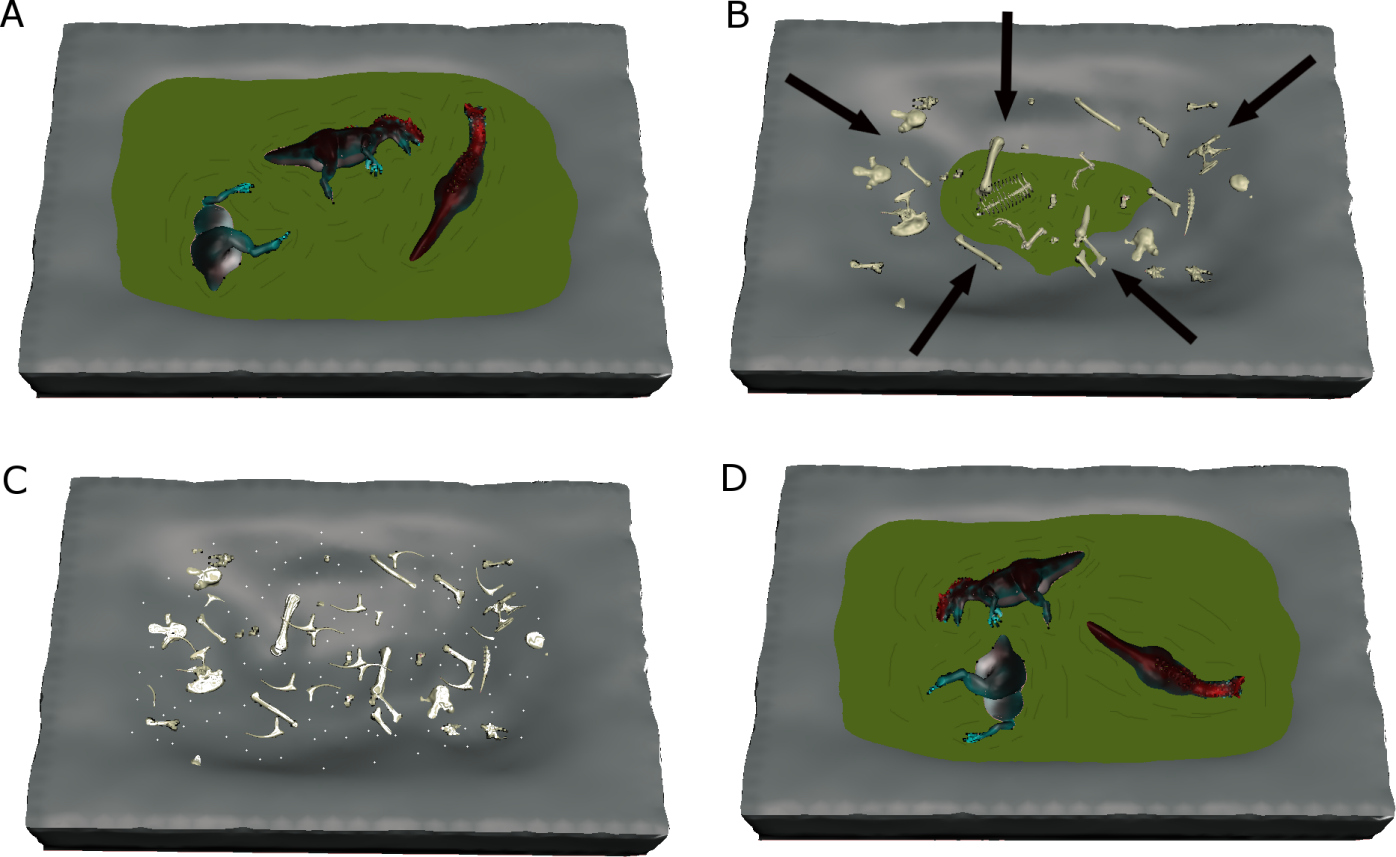
Conceptual model of the CLDQ depositional system. (A) Dinosaur carcasses are washed into the CLDQ deposit during a flood stage. High rates of organic matter decay leads to hypereutrophy, calcite and barite precipitation, and discourages biostratinomic influences (e.g., scavenging). (B) As water levels recede during drier conditions, bones that were not buried during the flood stage remain at the surface. (C) During arid conditions, bones present at the surface undergo weathering and abrasion from subaerial exposure, generating the intramatrix bone fragments present at the CLDQ. (D) Floodstage returns, incorporating new carcasses and re-worked bones and fragments to the deposit. The cycle repeats until the deposit maintains a higher water table, producing the limestone above the bone-bearing siltstone.

Conversely, the CLDQ assemblage exhibits considerably greater diversity in the degree of abrasion of IBFs with a mixture of angular and rounded fragments. The matrix at the CLDQ is a fine calcareous mudstone, representing a typically low-energy system that likely was ephemeral. However, the fragments possess a hydraulic equivalence of fine sand, suggesting that the fragments are derived from autochthonous or parautochthonous remains of locally weathered or crushed bones rather than an allochthonous/transported source (Fernandez-Jalvo & Andrews, 2003). As such, the abrasion/rounding observed on fragments is more likely to be the result of re-working and exposure than from fluvial transport (Fernandez-Jalvo & Andrews, 2003). The physical characteristics of the bone fragments of CLDQ suggest variable taphonomic histories among the fragments; angular fragments suggest weathering or pulverization followed immediately by burial while rounded fragments suggest prolonged exposure and re-working (Fernandez-Jalvo & Andrews, 2003) (Figs. 9A–9D).

The majority of macrovertebrate remains at CLDQ possess no apparent signs of weathering, suggesting an approximate subaerial exposure time of two years or less (*sensu* Gates, 2005), with rapid burial or submersion in water halting the progression of weathering and abrasion (Gifford, 1985). However, the variable angularity of the small bone fragments indicates that some bones were exposed longer than others (Fernandez-Jalvo & Andrews, 2003). Behrensmeyer’s (1978) temporal scale for bone weathering in arid environments indicates that remains can be broken down to fragments on a temporal scale of 6–15 years (Behrensmeyer’s Stage 5, 1978). Behrensmeyer (1978) also observed that rates of bone weathering can be increased with the crystallization of salts (Na_2_CO_3_, NaCl) on bone surfaces, producing cracking, splitting, and fragmentation. Similarly, Hare (1974) demonstrated that the organic breakdown of bones can be enhanced by fluctuating temperature and humidity. The broad distribution and variable angularity of bone fragments throughout the CLDQ matrix suggests that bones exposed at the surface may have been similarly weathered and degraded during fluctuations in moisture, and suggest that the CLDQ deposit may broadly represent a temporal range of at least 10–20 years for accumulation. However, the number of moisture cycles and depositional events are not fully understood at this time.

Thin section analyses of IBFs within the CLDQ matrix show the presence of pyrite crystals within the void spaces of bone fragments (Fig. 7). The presence of pyrite is indicative of microbially-precipitated sulfides infilling of bone pore space from the dissolution and re-precipitation of organic material in bone (e.g., collagen) (Carpenter, 2007; Wings, 2004; Bodzioch, 2015), implying that the bone fragments were pedogenically reworked following initial permineralization (Jennings & Hasiotis, 2006). This suggests that the total CLDQ assemblage, both intramatrix fragments and the well-documented macrovertebrate fauna, formed from multiple depositional events—perhaps seasonal—and not from a single catastrophic episode (*sensu* Gates, 2005). If the CLDQ had formed from a single event, multiple abrasion and weathering signatures on bone fragments would not be expected. Thus, the interpretation of the CLDQ as an attritional ephemeral deposit is further supported by the apparent lack of abundant identifiable freshwater microvertebrate remains; the lack of permanent water would reduce the abundance of freshwater taxa such as fish, turtles, and crocodilians. Meanwhile, seasonal fluctuations in the local water table related to the monsoonal climate patterns of the Late Jurassic in this region (e.g., Hallam, 1993; Rees, Ziegler & Valdes, 2000; Sellwood & Valdes, 2008) would have rejuvenated mobilization of fragments that otherwise remained at the surface (Fig. 9D).

### Paleoecological inferences

While geochemical and sedimentological data can assist in reconstructing the depositional environment and taphonomic history of the CLDQ (Figs. 9A–9D), the question remains—Why is there a dominating abundance of *Allosaurus* remains at the Cleveland-Lloyd Dinosaur Quarry?

Most multi-taxa bonebeds in the Morrison Formation are dominated by large herbivorous dinosaurs (Dodson et al., 1980), though many Morrison Formation bonebeds also include remains of theropods, commonly *Allosaurus* (Dodson et al., 1980). However, predator–dominant localities are known from other units in the Mesozoic system, such as the Upper Chinle *Coelophysis* quarry from Ghost Ranch, New Mexico (Schwartz & Gillette, 1994) and the Upper Horseshoe Canyon *Albertosaurus* bonebed from southern Alberta (Eberth & Currie, 2010). The nature of these accumulations are both interpreted to be the result of seasonally-influenced events; a drought-induced death assemblage of *Coelophysis* carcasses that were transported post-mortem by subsequent fluvial current (Schwartz & Gillette, 1994), and a storm-induced flooding event either directly or indirectly resulting in the death of a population of *Albertosaurus* (Eberth & Currie, 2010). Similarly, the CLDQ taphonomic data presented here also supports an interpretation of a post-mortem attritional accumulation of carcasses due to season fluctuations. However, it is also possible that the *Allosaurus* specimens from CLDQ died at, or very near the quarry.

Previous studies of the Morrison Formation have concluded that it represents a climate system dominated by strong seasonality consisting of periods of aridity during weak monsoons and sub-humid conditions during stronger monsoons, similar to climates seen in modern savannahs (Turner & Peterson, 2004; Parrish, Peterson & Turner, 2004; Tanner, Galli & Lucas, 2014). While such climate interpretations generally agree with taphonomic reconstructions of various Morrison Formation bonebeds, including the CLDQ, they can also offer insight into paleoecological interpretations that may have contributed to the quarry assemblage.

Fossil accumulations of multiple individuals of a single species are not necessarily indicative of complex familial or social behaviors. In modern savannahs, seasonal aridity brings grazing animals that are typically solitary together into larger groups near evaporating bodies of water (Western, 1975). Extant archosaurs, such as the Common Ostrich (*Struthio camelus*) and the Spectacled Caiman (*Caiman crocodilus crocodilus*) congregate in increased numbers during extreme seasonal changes, either for breeding or following food sources, leading to increased mortality during times of increased aridity or drought (e.g., Staton & Dixon, 1975; Knight, 1995). Furthermore, the reproductive cycles of many of these animals are tied to environmental and seasonal fluctuations, where fecundity, ovulation, and birth occur near an oncoming wet season (Hanks, 1979).

Breeding or nesting sites are relatively rare in the Morrison Formation; only nine such localities have been described to date (Bray & Hirsch, 1998). Although there is no direct evidence of the CLDQ being a breeding site for *Allosaurus*, a single fossilized egg possessing prismatoolithid eggshell was collected from the quarry (Hirsch et al., 1989), and has been attributed to *Allosaurus* due to the high frequency of the taxon in the quarry and the presence of perinatal remains of *Allosaurus* recovered from similar eggs from the Morrison (Madsen Jr, 1991; Carrano, Mateus & Mitchell, 2013). Furthermore, the CLDQ egg possesses an abnormally thick shell layer (Hirsch et al., 1989), which is frequently caused by environmental or seasonal stress (Mills, Marche & Faure, 1987; Hirsch, 2001). Though a single egg does not directly suggest that the CLDQ was a breeding site for *Allosaurus* or any other taxon, eggs could potentially have survived short distance transport in a fluvial system (e.g., Jackson, Schmidt & Oser, 2013).

Given the extreme seasonal variability in the Late Jurassic (e.g., Hallam, 1993) and the evidence described here for an attritional accumulation for the CLDQ, *Allosaurus* may have been congregating seasonally in the vicinity for a number of reasons, such as breeding, food, or water. With increased aridity, mortality rates may have been higher, producing carcasses that would mobilize during subsequent wet seasons similar to what is observed with extant archosaur carcasses during strong seasonality (Weigelt, 1989; Knight, 1995; Staton & Dixon, 1975). Transport processes and fluctuations in the water table could contribute to the disarticulated nature of the carcasses in the deposit (Gates, 2005). Further geochemical and isotopic analyses of remains recovered from CLDQ may help to evaluate whether *Allosaurus* were congregating before death and deposition, giving the bones similar isotopic and trace metal signatures, or, alternatively, were transported in from across the landscape post-mortem, bringing together a wide variety of isotopic and trace metal signatures. Further analyses may also help to determine whether the *Allosaurus* died at the CLDQ pond or elsewhere.

## Conclusions

The Cleveland-Lloyd Dinosaur Quarry is a potentially significant source of data for understanding Jurassic dinosaur paleoecology. However, interpretation is constrained by the taphonomic and environmental framework the quarry represents. Two new lines of evidence, sediment geochemistry and intramatrix bone fragment abrasion patterns, support previous conclusions that the CLDQ represents an ephemeral, seasonally dry pond. Furthermore, both data sets support the interpretation that the CLDQ was formed from multiple depositional events. In addition, each data set can add to the current understanding of CLDQ taphonomy.

New geochemical data show the quarry is enriched in heavy metals and sulfide minerals in stark contrast with the surrounding Morrison Formation strata, including mudstones at the same stratigraphic level surrounding the quarry deposit. While diagenetic processes certainly contributed to the heavy metal composition of CLDQ materials, the presence of sulfides and calcite/barite nodules in the CLDQ sediments suggest a hypereutrophic environment resulting from the decay of many dinosaurs in a small depression. Significant numbers of rotting dinosaurs in a body of standing water would potentially have contributed to the heavy metal composition of the water column via bioaccumulation. High rates of organic matter decay would have led to hypoxia or anoxia and the subsequent formation of sulfide minerals and the calcareous soaps required to form the calcite nodules.

Finally, hypereutrophy can help explain the near total lack of gnawing and other biostratinomic effects seen in a typical freshwater ecosystem. While these new geochemical data do not inform where the dinosaurs found at the CLDQ died, hypereutrophy related to dinosaur carcass decay is one possible explanation for the lack of typical freshwater faunal remains and feeding traces at the CLDQ. Initial geochemical data presented here support this taphonomic framework; however, a more extensive analysis of bone geochemistry is needed to support this hypothesis. Analysis of sediment and bone from the Mygatt-Moore Quarry support these conclusions by providing strong contrast. Although heavy metals are present in MMQ, as expected from bonebed diagenesis, the other geochemical indicators of hypereutrophy are not present. The unique preservation of bone found at CLDQ is a result of chemical conditions not present in the more common depositional setting represented by MMQ.

Furthermore, quantitative and qualitative assessment of intramatrix bone fragments at the CLDQ and the new Johnsonville site indicate subtle but important differences in the depositional systems that produced the respective assemblages. While the bone fragments at both localities suggest a comparable hydraulic equivalence, the differences in the local lithologies and average abrasion profiles for bone fragments at each locality suggest considerable disparity in the genesis of each site. In particular, the taphonomic characteristics of the IBFs of the CLDQ assemblage, coupled with the quarry lithology, support the interpretation that the quarry assemblage was produced by a series of separate depositional events punctuated by periodic aridity. The IBF data presented here contribute to former taphonomic assessments of the CLDQ by providing previously uninvestigated sedimentological and micro-taphonomic insight into the origins of the quarry assemblage. Unidentifiable fossil fragments are often overlooked. While small, the utilization of IBF data in conjunction with associated macro-taphonomic and sedimentologic data has the potential to improve the resolution of complex and perplexing taphonomic questions.

##  Supplemental Information

10.7717/peerj.3368/supp-1 Supplemental Tables 1–3 Table S1: Raw XRF data for stratigraphic samples from the CLDQ vicinity, CLDQ bone and sediment samples, JONS bone and sediment samples, and MMQ bone and sediment samples; Table S2—Intramatrix bone fragment data from the Cleveland-Lloyd Dinosaur Quarry; Table S3—Intramatrix bone fragment data from the JONS site

## Additional Information and Declarations

### Field Study Permissions

The following information was supplied relating to field study approvals (i.e., approving body and any reference numbers): This work was conducted under the permit numbers UT12-002S and UT12-003E issued by the Bureau of Land Management.

### Data Availability

The following information was supplied regarding data availability: Peterson, Joseph (2016): Peterson et al. CLDQ Taphonomy Supplemental Tables. figshare. https://doi.org/10.6084/m9.figshare.4238906.v1.
