# Supplementary material for: New data towards the development of a comprehensive taphonomic framework for the Late Jurassic Cleveland-Lloyd Dinosaur Quarry, Central Utah
Source: PeerJ. 2017 Jun 6;5:e3368. doi: 10.7717/peerj.3368 (PMC5463971; doi:10.7717/peerj.3368)
Supplement: Supplemental Tables 1–3 [file peerj-05-3368-s001.docx]

Table S1: XRF Data

| Sample # | Mg | Mg Error | Al | Al Error | Si | Si Error | P | P Error |
| --- | --- | --- | --- | --- | --- | --- | --- | --- |
| U1S1 | 0.00E+00 | 1.41E+04 | 7.13E+03 | 1.44E+03 | 3.65E+04 | 1.16E+03 | 0.00E+00 | 3.34E+02 |
| U1S2 | 8.75E+03 | 2.83E+03 | 2.25E+04 | 9.91E+02 | 1.29E+05 | 1.55E+03 | 3.51E+02 | 1.78E+02 |
| U2 | 0.00E+00 | 2.97E+04 | 7.98E+03 | 2.42E+03 | 4.82E+04 | 1.63E+03 | 0.00E+00 | 5.06E+02 |
| U3 | 0.00E+00 | 2.33E+04 | 2.10E+04 | 2.58E+03 | 1.06E+05 | 2.29E+03 | 0.00E+00 | 5.46E+02 |
| U4 | 0.00E+00 | 9.02E+03 | 1.19E+04 | 1.35E+03 | 2.53E+05 | 3.04E+03 | 0.00E+00 | 5.97E+02 |
| U5 | 0.00E+00 | 1.88E+04 | 7.10E+03 | 2.26E+03 | 4.70E+04 | 1.68E+03 | 0.00E+00 | 4.97E+02 |
| U6 | 0.00E+00 | 7.54E+03 | 1.24E+04 | 1.23E+03 | 2.25E+05 | 2.68E+03 | 0.00E+00 | 5.95E+02 |
| U7 | 1.85E+04 | 6.46E+03 | 3.56E+04 | 2.40E+03 | 3.29E+05 | 3.87E+03 | 9.96E+02 | 5.18E+02 |
| U8 | 0.00E+00 | 1.87E+04 | 5.70E+04 | 4.16E+03 | 2.90E+05 | 4.02E+03 | 1.74E+03 | 6.54E+02 |
| U9 | 1.18E+04 | 6.87E+03 | 1.49E+04 | 2.03E+03 | 3.72E+05 | 3.75E+03 | 0.00E+00 | 9.64E+02 |
| U10 | 1.28E+04 | 6.16E+03 | 1.31E+04 | 1.73E+03 | 3.83E+05 | 3.47E+03 | 9.09E+02 | 5.80E+02 |
| U11 | 0.00E+00 | 1.28E+04 | 2.67E+04 | 2.11E+03 | 2.34E+05 | 2.96E+03 | 0.00E+00 | 9.91E+02 |
| U12 | 0.00E+00 | 1.91E+04 | 2.12E+04 | 2.57E+03 | 4.23E+05 | 4.47E+03 | 0.00E+00 | 1.53E+03 |
| U13 | 1.74E+04 | 9.50E+03 | 2.05E+04 | 2.35E+03 | 1.42E+05 | 2.37E+03 | 0.00E+00 | 5.49E+02 |
| U14 | 0.00E+00 | 9.09E+03 | 3.98E+04 | 2.44E+03 | 2.42E+05 | 2.90E+03 | 7.70E+02 | 4.30E+02 |
| U15 | 0.00E+00 | 1.16E+04 | 1.91E+04 | 1.71E+03 | 1.45E+05 | 2.20E+03 | 0.00E+00 | 4.52E+02 |
| U16 | 0.00E+00 | 6.93E+03 | 1.33E+04 | 1.36E+03 | 4.61E+05 | 3.56E+03 | 1.11E+03 | 5.39E+02 |
| U16-1 | 0.00E+00 | 8.04E+03 | 9.48E+03 | 1.49E+03 | 2.89E+05 | 3.29E+03 | 0.00E+00 | 7.24E+02 |
| U17A | 0.00E+00 | 7.77E+03 | 2.62E+04 | 1.96E+03 | 2.48E+05 | 2.94E+03 | 6.75E+02 | 4.42E+02 |
| U17B | 1.13E+04 | 5.98E+03 | 3.81E+04 | 2.45E+03 | 3.28E+05 | 3.80E+03 | 8.82E+02 | 5.16E+02 |
| U18A | 0.00E+00 | 5.28E+03 | 3.73E+04 | 1.83E+03 | 2.97E+05 | 3.25E+03 | 6.67E+02 | 3.92E+02 |
| U18B | 1.12E+04 | 5.22E+03 | 2.89E+04 | 1.81E+03 | 2.14E+05 | 2.63E+03 | 0.00E+00 | 5.02E+02 |
| U19 | 0.00E+00 | 4.76E+03 | 1.61E+04 | 1.19E+03 | 3.48E+05 | 3.70E+03 | 0.00E+00 | 5.81E+02 |
| U20 | 0.00E+00 | 8.97E+03 | 1.94E+04 | 1.64E+03 | 1.56E+05 | 2.06E+03 | 0.00E+00 | 4.15E+02 |
| U21A | 0.00E+00 | 9.63E+03 | 2.33E+04 | 2.30E+03 | 3.61E+05 | 4.11E+03 | 1.56E+03 | 6.19E+02 |
| U22 - JONS | 9.43E+03 | 3.73E+03 | 3.42E+04 | 1.63E+03 | 2.54E+05 | 2.85E+03 | 9.15E+02 | 3.34E+02 |
| U23 | 0.00E+00 | 9.06E+03 | 1.93E+04 | 1.93E+03 | 3.33E+05 | 3.86E+03 | 0.00E+00 | 7.93E+02 |
| U24 | 1.63E+04 | 6.56E+03 | 3.38E+04 | 2.41E+03 | 3.64E+05 | 3.63E+03 | 1.51E+03 | 5.77E+02 |
| U25 | 0.00E+00 | 2.58E+04 | 1.15E+04 | 2.58E+03 | 1.43E+05 | 3.01E+03 | 0.00E+00 | 7.14E+02 |
| U26 | 0.00E+00 | 1.45E+04 | 9.35E+03 | 2.17E+03 | 1.44E+05 | 2.68E+03 | 0.00E+00 | 6.27E+02 |
| U27 | 0.00E+00 | 8.37E+03 | 4.91E+04 | 2.69E+03 | 4.10E+05 | 3.82E+03 | 3.21E+03 | 6.26E+02 |
| U28 | 0.00E+00 | 6.02E+03 | 2.51E+04 | 1.75E+03 | 2.64E+05 | 3.17E+03 | 2.36E+04 | 7.26E+02 |
| U29 | 0.00E+00 | 4.55E+03 | 1.73E+04 | 1.22E+03 | 3.94E+05 | 3.53E+03 | 1.57E+03 | 4.37E+02 |
| U30 | 1.25E+04 | 8.30E+03 | 5.64E+04 | 3.66E+03 | 5.22E+05 | 4.17E+03 | 9.73E+03 | 9.07E+02 |
| U31 | 1.02E+04 | 2.87E+03 | 4.17E+04 | 1.44E+03 | 3.54E+05 | 3.60E+03 | 3.11E+03 | 3.24E+02 |
| U32 | 1.08E+04 | 4.78E+03 | 3.19E+04 | 1.94E+03 | 3.82E+05 | 3.36E+03 | 4.82E+03 | 5.44E+02 |
| U33 | 0.00E+00 | 9.04E+03 | 4.96E+04 | 2.42E+03 | 2.67E+05 | 3.24E+03 | 7.43E+03 | 5.07E+02 |
| U34 | 5.02E+03 | 2.63E+03 | 4.89E+04 | 1.42E+03 | 2.59E+05 | 2.06E+03 | 1.07E+03 | 2.40E+02 |
| U34A | 0.00E+00 | 4.80E+03 | 1.88E+04 | 1.48E+03 | 3.58E+05 | 3.31E+03 | 8.68E+02 | 4.81E+02 |
| U34B | 8.11E+03 | 3.74E+03 | 3.28E+04 | 1.67E+03 | 3.92E+05 | 3.58E+03 | 0.00E+00 | 6.43E+02 |
| U35 | 0.00E+00 | 4.95E+03 | 2.86E+04 | 1.58E+03 | 4.23E+05 | 3.31E+03 | 0.00E+00 | 7.15E+02 |
| U36 | 0.00E+00 | 5.71E+03 | 6.79E+04 | 2.75E+03 | 2.95E+05 | 3.38E+03 | 1.26E+03 | 4.43E+02 |
| U37 | 0.00E+00 | 1.22E+04 | 1.33E+04 | 1.58E+03 | 2.56E+05 | 3.09E+03 | 0.00E+00 | 6.44E+02 |
| U37S1 | 0.00E+00 | 2.39E+04 | 4.17E+03 | 1.95E+03 | 7.54E+04 | 2.05E+03 | 0.00E+00 | 5.07E+02 |
| CLS | 0.00E+00 | 2.16E+04 | 8.09E+03 | 2.21E+03 | 3.06E+04 | 1.31E+03 | 1.13E+05 | 1.70E+03 |
| CLN | 0.00E+00 | 8.63E+03 | 7.62E+03 | 1.01E+03 | 2.46E+04 | 5.82E+02 | 1.16E+05 | 8.95E+02 |

| Sample # | S | S Error | Cl | Cl Error | K | K Error | Ca | Ca Error |
| --- | --- | --- | --- | --- | --- | --- | --- | --- |
| U1S1 | 1.14E+03 | 1.39E+02 | 0.00E+00 | 6.55E+01 | 5.10E+03 | 5.10E+02 | 3.38E+05 | 4.55E+03 |
| U1S2 | 4.26E+02 | 6.39E+01 | 0.00E+00 | 3.96E+01 | 1.89E+04 | 8.30E+02 | 7.90E+04 | 2.35E+03 |
| U2 | 2.81E+03 | 2.40E+02 | 0.00E+00 | 1.20E+02 | 5.89E+03 | 6.26E+02 | 4.76E+05 | 5.98E+03 |
| U3 | 3.13E+01 | 3.87E+00 | 0.00E+00 | 1.09E+02 | 1.96E+04 | 9.47E+02 | 2.83E+05 | 4.40E+03 |
| U4 | 1.22E+03 | 1.39E+02 | 0.00E+00 | 7.23E+01 | 3.33E+03 | 3.94E+02 | 1.12E+05 | 2.54E+03 |
| U5 | 1.18E+03 | 1.92E+02 | 0.00E+00 | 9.40E+01 | 5.65E+03 | 5.43E+02 | 4.07E+05 | 5.59E+03 |
| U6 | 2.40E+01 | 3.09E+00 | 0.00E+00 | 6.19E+01 | 6.55E+03 | 4.64E+02 | 1.18E+05 | 2.46E+03 |
| U7 | 8.01E+01 | 7.02E+00 | 0.00E+00 | 9.72E+01 | 1.64E+04 | 1.02E+03 | 1.52E+04 | 1.56E+03 |
| U8 | 5.95E+01 | 6.03E+00 | 0.00E+00 | 1.31E+02 | 4.91E+04 | 1.80E+03 | 1.11E+04 | 1.76E+03 |
| U9 | 1.44E+01 | 2.47E+00 | 0.00E+00 | 1.06E+02 | 7.65E+03 | 5.47E+02 | 9.04E+04 | 2.31E+03 |
| U10 | 1.76E+01 | 2.53E+00 | 0.00E+00 | 9.60E+01 | 2.23E+03 | 3.75E+02 | 1.09E+05 | 2.45E+03 |
| U11 | 7.45E+01 | 4.91E+00 | 0.00E+00 | 8.85E+01 | 1.44E+04 | 7.40E+02 | 1.19E+05 | 2.73E+03 |
| U12 | 2.90E+01 | 4.51E+00 | 0.00E+00 | 1.44E+02 | 2.28E+03 | 4.70E+02 | 1.40E+05 | 3.34E+03 |
| U13 | 1.00E+03 | 1.57E+02 | 0.00E+00 | 9.60E+01 | 1.13E+04 | 7.54E+02 | 3.31E+05 | 5.00E+03 |
| U14 | 1.74E+03 | 1.68E+02 | 3.74E+02 | 6.78E+01 | 2.76E+04 | 1.12E+03 | 1.19E+05 | 3.12E+03 |
| U15 | 3.18E+01 | 4.35E+00 | 0.00E+00 | 6.74E+01 | 9.99E+03 | 6.88E+02 | 2.54E+05 | 4.24E+03 |
| U16 | 8.05E+01 | 4.91E+00 | 0.00E+00 | 9.65E+01 | 1.50E+03 | 3.04E+02 | 3.80E+04 | 1.43E+03 |
| U16-1 | 2.47E+01 | 3.12E+00 | 0.00E+00 | 1.29E+02 | 6.09E+02 | 2.60E+02 | 1.16E+05 | 2.43E+03 |
| U17A | 7.56E+01 | 4.65E+00 | 1.91E+02 | 6.21E+01 | 1.35E+04 | 7.00E+02 | 1.03E+05 | 2.49E+03 |
| U17B | 6.83E+01 | 6.09E+00 | 0.00E+00 | 1.03E+02 | 6.57E+03 | 6.85E+02 | 4.16E+04 | 2.28E+03 |
| U18A | 4.04E+01 | 4.27E+00 | 2.91E+02 | 5.68E+01 | 1.99E+04 | 9.33E+02 | 9.07E+03 | 1.09E+03 |
| U18B | 6.76E+01 | 5.56E+00 | 1.85E+02 | 5.05E+01 | 1.01E+04 | 6.65E+02 | 1.56E+05 | 3.22E+03 |
| U19 | 3.72E+03 | 1.69E+02 | 2.57E+02 | 4.77E+01 | 1.06E+03 | 3.52E+02 | 2.43E+04 | 1.49E+03 |
| U20 | 1.44E+03 | 1.27E+02 | 2.07E+02 | 4.67E+01 | 8.64E+03 | 6.68E+02 | 3.02E+05 | 4.43E+03 |
| U21A | 2.90E+03 | 2.33E+02 | 4.13E+02 | 8.20E+01 | 2.80E+03 | 5.46E+02 | 1.24E+05 | 3.49E+03 |
| U22 - JONS | 1.28E+03 | 1.16E+02 | 8.64E+02 | 5.85E+01 | 1.47E+04 | 7.82E+02 | 2.67E+04 | 1.58E+03 |
| U23 | 4.17E+01 | 4.94E+00 | 2.70E+02 | 6.80E+01 | 3.00E+03 | 5.02E+02 | 1.07E+05 | 3.11E+03 |
| U24 | 3.74E+01 | 3.80E+00 | 3.53E+02 | 7.70E+01 | 6.08E+03 | 6.23E+02 | 3.62E+04 | 1.97E+03 |
| U25 | 9.63E+02 | 2.09E+02 | 1.64E+02 | 8.02E+01 | 2.00E+03 | 4.27E+02 | 3.16E+05 | 4.88E+03 |
| U26 | 1.48E+03 | 1.99E+02 | 2.25E+02 | 7.15E+01 | 1.47E+03 | 4.37E+02 | 3.52E+05 | 5.37E+03 |
| U27 | 5.77E+01 | 4.86E+00 | 0.00E+00 | 1.17E+02 | 7.26E+03 | 7.19E+02 | 1.67E+04 | 1.48E+03 |
| U28 | 3.26E+01 | 4.17E+00 | 0.00E+00 | 1.10E+02 | 9.24E+03 | 6.63E+02 | 6.52E+04 | 2.35E+03 |
| U29 | 2.03E+03 | 1.35E+02 | 2.21E+02 | 4.81E+01 | 1.57E+03 | 2.66E+02 | 4.94E+03 | 5.31E+02 |
| U30 | 3.39E+01 | 4.28E+00 | 0.00E+00 | 1.50E+02 | 2.32E+04 | 1.42E+03 | 3.72E+04 | 2.63E+03 |
| U31 | 2.04E+03 | 1.07E+02 | 1.51E+02 | 3.96E+01 | 6.44E+03 | 5.51E+02 | 1.53E+04 | 1.16E+03 |
| U32 | 3.51E+01 | 3.47E+00 | 2.38E+02 | 6.19E+01 | 1.26E+04 | 8.01E+02 | 1.80E+04 | 1.45E+03 |
| U33 | 5.86E+01 | 5.51E+00 | 9.63E+01 | 6.04E+01 | 1.39E+04 | 8.10E+02 | 2.96E+04 | 1.74E+03 |
| U34 | 3.61E+02 | 6.59E+01 | 1.13E+02 | 3.49E+01 | 2.23E+04 | 1.13E+03 | 8.17E+03 | 1.26E+03 |
| U34A | 4.74E+01 | 3.80E+00 | 1.18E+02 | 5.45E+01 | 3.03E+03 | 3.76E+02 | 9.42E+03 | 8.72E+02 |
| U34B | 3.35E+01 | 3.91E+00 | 1.14E+02 | 5.14E+01 | 1.39E+04 | 8.00E+02 | 4.77E+03 | 8.26E+02 |
| U35 | 1.47E+02 | 6.34E+00 | 1.43E+02 | 5.42E+01 | 2.97E+03 | 3.35E+02 | 1.75E+03 | 3.57E+02 |
| U36 | 7.49E+01 | 6.10E+00 | 0.00E+00 | 9.31E+01 | 2.19E+04 | 9.97E+02 | 5.33E+03 | 9.34E+02 |
| U37 | 5.76E+01 | 4.76E+00 | 1.75E+02 | 5.65E+01 | 1.50E+03 | 3.25E+02 | 1.47E+05 | 2.86E+03 |
| U37S1 | 2.32E+03 | 2.27E+02 | 0.00E+00 | 9.80E+01 | 9.36E+02 | 3.90E+02 | 3.94E+05 | 5.37E+03 |
| CLS | 1.10E+04 | 4.22E+02 | 1.92E+02 | 6.64E+01 | 3.33E+00 | 5.30E-01 | 4.15E+05 | 5.59E+03 |
| CLN | 1.06E+04 | 2.00E+02 | 2.80E+02 | 3.27E+01 | 3.18E+00 | 3.30E-01 | 3.80E+05 | 3.08E+03 |

| Sample # | Sc | Sc Error | Ti | Ti Error | V | V Error | Cr | Cr Error |
| --- | --- | --- | --- | --- | --- | --- | --- | --- |
| U1S1 | 8.37E+03 | 7.61E+01 | 0.00E+00 | 4.30E-01 | 0.00E+00 | 4.00E-01 | 0.00E+00 | 3.80E-01 |
| U1S2 | 1.12E+04 | 2.75E+01 | 2.18E+03 | 1.86E+02 | 0.00E+00 | 1.19E+02 | 1.16E+02 | 5.00E+01 |
| U2 | 6.57E+03 | 1.05E+02 | 0.00E+00 | 4.70E-01 | 0.00E+00 | 4.20E-01 | 0.00E+00 | 3.90E-01 |
| U3 | 8.70E+03 | 6.96E+01 | 1.52E+03 | 3.01E+02 | 0.00E+00 | 3.90E-01 | 0.00E+00 | 1.16E+02 |
| U4 | 1.13E+04 | 3.10E+01 | 5.83E+02 | 1.89E+02 | 0.00E+00 | 3.70E-01 | 0.00E+00 | 3.50E-01 |
| U5 | 7.46E+03 | 1.10E+02 | 0.00E+00 | 6.10E-01 | 0.00E+00 | 1.25E+02 | 0.00E+00 | 5.00E-01 |
| U6 | 1.12E+04 | 3.22E+01 | 6.28E+02 | 1.28E+02 | 0.00E+00 | 3.70E-01 | 0.00E+00 | 3.50E-01 |
| U7 | 1.17E+04 | 2.80E+01 | 2.36E+03 | 3.37E+02 | 0.00E+00 | 4.80E-01 | 0.00E+00 | 1.29E+02 |
| U8 | 1.12E+04 | 3.88E+01 | 5.16E+03 | 3.51E+02 | 0.00E+00 | 4.70E-01 | 1.92E+02 | 9.62E+01 |
| U9 | 1.15E+04 | 2.61E+01 | 5.94E+02 | 1.47E+02 | 0.00E+00 | 3.40E-01 | 0.00E+00 | 3.10E-01 |
| U10 | 1.13E+04 | 2.69E+01 | 3.22E+02 | 1.44E+02 | 0.00E+00 | 3.20E-01 | 0.00E+00 | 3.00E-01 |
| U11 | 1.09E+04 | 3.40E+01 | 1.83E+03 | 2.03E+02 | 0.00E+00 | 3.40E-01 | 9.73E+01 | 5.97E+01 |
| U12 | 1.09E+04 | 4.91E+01 | 1.02E+03 | 2.07E+02 | 0.00E+00 | 5.00E-01 | 0.00E+00 | 4.50E-01 |
| U13 | 8.27E+03 | 9.12E+01 | 1.06E+03 | 3.35E+02 | 0.00E+00 | 4.70E-01 | 0.00E+00 | 4.40E-01 |
| U14 | 1.04E+04 | 4.37E+01 | 3.26E+03 | 2.67E+02 | 0.00E+00 | 1.72E+02 | 1.96E+02 | 7.54E+01 |
| U15 | 9.28E+03 | 7.22E+01 | 1.29E+03 | 2.76E+02 | 0.00E+00 | 4.60E-01 | 0.00E+00 | 1.07E+02 |
| U16 | 1.22E+04 | 1.20E+01 | 1.28E+03 | 1.21E+02 | 0.00E+00 | 3.30E-01 | 0.00E+00 | 3.00E-01 |
| U16-1 | 1.13E+04 | 2.99E+01 | 4.53E+02 | 1.25E+02 | 0.00E+00 | 3.70E-01 | 0.00E+00 | 3.50E-01 |
| U17A | 1.12E+04 | 2.89E+01 | 1.87E+03 | 1.86E+02 | 0.00E+00 | 3.20E-01 | 0.00E+00 | 8.85E+01 |
| U17B | 1.15E+04 | 3.02E+01 | 2.38E+03 | 2.34E+02 | 0.00E+00 | 4.50E-01 | 0.00E+00 | 1.27E+02 |
| U18A | 1.20E+04 | 1.87E+01 | 3.04E+03 | 2.03E+02 | 0.00E+00 | 1.35E+02 | 1.33E+02 | 6.02E+01 |
| U18B | 1.05E+04 | 4.62E+01 | 1.85E+03 | 2.85E+02 | 0.00E+00 | 4.00E-01 | 0.00E+00 | 3.90E-01 |
| U19 | 1.23E+04 | 1.39E+01 | 1.28E+03 | 3.47E+02 | 0.00E+00 | 4.30E-01 | 0.00E+00 | 4.10E-01 |
| U20 | 8.67E+03 | 7.10E+01 | 1.31E+03 | 3.12E+02 | 0.00E+00 | 3.90E-01 | 0.00E+00 | 3.70E-01 |
| U21A | 1.07E+04 | 5.01E+01 | 1.96E+03 | 2.80E+02 | 0.00E+00 | 4.80E-01 | 0.00E+00 | 4.40E-01 |
| U22 - JONS | 1.20E+04 | 1.80E+01 | 2.35E+03 | 1.79E+02 | 0.00E+00 | 3.70E-01 | 1.30E+02 | 5.42E+01 |
| U23 | 1.11E+04 | 4.14E+01 | 9.60E+02 | 2.59E+02 | 0.00E+00 | 4.50E-01 | 0.00E+00 | 4.20E-01 |
| U24 | 1.17E+04 | 2.21E+01 | 1.68E+03 | 2.11E+02 | 0.00E+00 | 3.60E-01 | 1.35E+02 | 7.78E+01 |
| U25 | 8.63E+03 | 8.77E+01 | 4.73E+02 | 2.83E+02 | 0.00E+00 | 4.90E-01 | 0.00E+00 | 4.60E-01 |
| U26 | 8.20E+03 | 9.75E+01 | 6.41E+02 | 3.87E+02 | 0.00E+00 | 4.90E-01 | 0.00E+00 | 4.60E-01 |
| U27 | 1.21E+04 | 1.52E+01 | 3.16E+03 | 3.22E+02 | 0.00E+00 | 3.70E-01 | 0.00E+00 | 1.35E+02 |
| U28 | 1.15E+04 | 3.01E+01 | 1.43E+03 | 1.86E+02 | 0.00E+00 | 4.30E-01 | 1.03E+02 | 6.41E+01 |
| U29 | 1.27E+04 | 3.32E+00 | 8.09E+02 | 9.49E+01 | 0.00E+00 | 3.60E-01 | 0.00E+00 | 3.30E-01 |
| U30 | 1.13E+04 | 3.32E+01 | 3.48E+03 | 3.79E+02 | 0.00E+00 | 2.88E+02 | 2.66E+02 | 1.37E+02 |
| U31 | 1.23E+04 | 1.24E+01 | 2.23E+03 | 2.86E+02 | 0.00E+00 | 4.30E-01 | 0.00E+00 | 1.02E+02 |
| U32 | 1.20E+04 | 1.51E+01 | 2.03E+03 | 2.12E+02 | 0.00E+00 | 3.30E-01 | 1.13E+02 | 7.11E+01 |
| U33 | 1.18E+04 | 2.32E+01 | 2.84E+03 | 2.17E+02 | 1.49E+02 | 9.93E+01 | 1.75E+02 | 6.53E+01 |
| U34 |  |  | 3.12E+03 | 2.60E+02 | 2.09E+02 | 1.23E+02 | 1.90E+02 | 7.49E+01 |
| U34A | 1.24E+04 | 7.83E+00 | 8.00E+02 | 1.90E+02 | 0.00E+00 | 3.30E-01 | 8.33E+01 | 5.30E+01 |
| U34B | 1.22E+04 | 1.32E+01 | 2.12E+03 | 1.81E+02 | 0.00E+00 | 1.32E+02 | 1.21E+02 | 6.66E+01 |
| U35 | 1.27E+04 | 3.03E+00 | 2.36E+03 | 1.35E+02 | 0.00E+00 | 3.10E-01 | 0.00E+00 | 2.80E-01 |
| U36 | 1.20E+04 | 1.99E+01 | 5.20E+03 | 2.70E+02 | 0.00E+00 | 1.72E+02 | 2.46E+02 | 7.01E+01 |
| U37 | 1.08E+04 | 3.92E+01 | 1.07E+03 | 1.71E+02 | 0.00E+00 | 3.90E-01 | 0.00E+00 | 3.70E-01 |
| U37S1 | 7.76E+03 | 9.60E+01 | 0.00E+00 | 5.20E-01 | 0.00E+00 | 4.60E-01 | 0.00E+00 | 1.21E+02 |
| CLS | 7.48E+03 | 9.68E+01 | 0.00E+00 | 7.10E-01 | 0.00E+00 | 4.40E-01 | 2.59E+02 | 1.34E+02 |
| CLN | 5.57E+03 | 7.98E+01 | 0.00E+00 | 1.41E+00 | 0.00E+00 | 2.90E-01 | 3.32E+02 | 8.11E+01 |

| Sample # | Mn | Mn Error | Fe | Fe Error | Ni | Ni Error | Cu | Cu Error |
| --- | --- | --- | --- | --- | --- | --- | --- | --- |
| U1S1 | 2.24E+02 | 8.50E+01 | 2.92E+03 | 1.55E+02 | 0.00E+00 | 5.14E+01 | 0.00E+00 | 3.24E+01 |
| U1S2 | 3.42E+02 | 7.46E+01 | 2.65E+04 | 4.47E+02 | 0.00E+00 | 3.78E+01 | 0.00E+00 | 2.34E+01 |
| U2 | 5.52E+02 | 1.12E+02 | 4.36E+03 | 2.00E+02 | 0.00E+00 | 6.01E+01 | 0.00E+00 | 3.83E+01 |
| U3 | 3.82E+02 | 9.36E+01 | 1.72E+04 | 4.36E+02 | 0.00E+00 | 5.45E+01 | 0.00E+00 | 3.30E+01 |
| U4 | 0.00E+00 | 9.82E+01 | 1.30E+03 | 1.03E+02 | 0.00E+00 | 4.80E+01 | 0.00E+00 | 2.73E+01 |
| U5 | 7.81E+02 | 1.46E+02 | 3.09E+03 | 2.02E+02 | 0.00E+00 | 7.03E+01 | 0.00E+00 | 4.47E+01 |
| U6 | 0.00E+00 | 1.04E+02 | 1.87E+03 | 1.16E+02 | 0.00E+00 | 4.58E+01 | 0.00E+00 | 2.81E+01 |
| U7 | 0.00E+00 | 1.50E+02 | 5.01E+04 | 9.23E+02 | 0.00E+00 | 7.13E+01 | 0.00E+00 | 4.21E+01 |
| U8 | 0.00E+00 | 1.54E+02 | 5.82E+04 | 1.01E+03 | 0.00E+00 | 6.93E+01 | 0.00E+00 | 4.14E+01 |
| U9 | 1.06E+02 | 6.65E+01 | 3.81E+03 | 1.53E+02 | 0.00E+00 | 4.46E+01 | 0.00E+00 | 2.81E+01 |
| U10 | 1.91E+02 | 6.78E+01 | 2.87E+03 | 1.28E+02 | 0.00E+00 | 4.17E+01 | 0.00E+00 | 2.63E+01 |
| U11 | 1.30E+02 | 6.86E+01 | 1.06E+04 | 3.07E+02 | 0.00E+00 | 4.43E+01 | 0.00E+00 | 2.74E+01 |
| U12 | 0.00E+00 | 1.47E+02 | 2.84E+03 | 1.92E+02 | 0.00E+00 | 7.12E+01 | 0.00E+00 | 4.82E+01 |
| U13 | 1.48E+03 | 1.71E+02 | 1.10E+04 | 4.22E+02 | 0.00E+00 | 6.76E+01 | 0.00E+00 | 4.47E+01 |
| U14 | 1.93E+02 | 8.16E+01 | 3.83E+04 | 6.37E+02 | 0.00E+00 | 5.01E+01 | 0.00E+00 | 3.25E+01 |
| U15 | 8.36E+02 | 1.35E+02 | 1.01E+04 | 3.89E+02 | 0.00E+00 | 6.06E+01 | 0.00E+00 | 3.73E+01 |
| U16 | 0.00E+00 | 7.93E+01 | 1.58E+03 | 9.96E+01 | 0.00E+00 | 4.38E+01 | 0.00E+00 | 2.91E+01 |
| U16-1 | 0.00E+00 | 1.02E+02 | 1.13E+03 | 9.36E+01 | 0.00E+00 | 4.65E+01 | 0.00E+00 | 2.74E+01 |
| U17A | 1.27E+02 | 6.40E+01 | 6.64E+03 | 1.85E+02 | 0.00E+00 | 4.18E+01 | 0.00E+00 | 2.51E+01 |
| U17B | 1.56E+02 | 9.88E+01 | 4.71E+04 | 8.46E+02 | 0.00E+00 | 6.36E+01 | 0.00E+00 | 3.86E+01 |
| U18A | 0.00E+00 | 1.13E+02 | 2.87E+04 | 5.91E+02 | 0.00E+00 | 5.42E+01 | 0.00E+00 | 3.37E+01 |
| U18B | 5.85E+02 | 1.08E+02 | 1.38E+04 | 4.01E+02 | 0.00E+00 | 5.41E+01 | 0.00E+00 | 3.36E+01 |
| U19 | 0.00E+00 | 1.25E+02 | 1.39E+04 | 4.31E+02 | 0.00E+00 | 5.81E+01 | 0.00E+00 | 3.22E+01 |
| U20 | 1.67E+03 | 1.50E+02 | 1.03E+04 | 3.42E+02 | 0.00E+00 | 5.50E+01 | 0.00E+00 | 3.20E+01 |
| U21A | 1.35E+03 | 1.74E+02 | 3.09E+04 | 7.43E+02 | 0.00E+00 | 6.82E+01 | 0.00E+00 | 4.66E+01 |
| U22 - JONS | 0.00E+00 | 9.80E+01 | 2.00E+04 | 4.57E+02 | 0.00E+00 | 4.45E+01 | 0.00E+00 | 2.75E+01 |
| U23 | 6.54E+02 | 1.29E+02 | 2.48E+04 | 6.24E+02 | 0.00E+00 | 5.96E+01 | 0.00E+00 | 3.85E+01 |
| U24 | 1.90E+02 | 8.30E+01 | 3.96E+04 | 6.36E+02 | 0.00E+00 | 5.04E+01 | 0.00E+00 | 3.25E+01 |
| U25 | 3.14E+03 | 2.41E+02 | 3.48E+03 | 2.13E+02 | 0.00E+00 | 6.54E+01 | 0.00E+00 | 4.14E+01 |
| U26 | 3.39E+03 | 2.56E+02 | 2.42E+03 | 1.91E+02 | 0.00E+00 | 6.81E+01 | 0.00E+00 | 4.04E+01 |
| U27 | 0.00E+00 | 1.06E+02 | 2.49E+04 | 5.03E+02 | 0.00E+00 | 5.24E+01 | 0.00E+00 | 3.44E+01 |
| U28 | 2.13E+02 | 9.37E+01 | 2.55E+04 | 5.80E+02 | 0.00E+00 | 5.85E+01 | 0.00E+00 | 3.29E+01 |
| U29 | 0.00E+00 | 8.41E+01 | 1.95E+03 | 1.14E+02 | 0.00E+00 | 4.33E+01 | 0.00E+00 | 2.79E+01 |
| U30 | 0.00E+00 | 3.00E+05 | 5.01E+04 | 8.16E+02 | 0.00E+00 | 3.00E+05 | 0.00E+00 | 3.00E+05 |
| U31 | 0.00E+00 | 1.22E+02 | 1.05E+04 | 3.82E+02 | 0.00E+00 | 5.83E+01 | 0.00E+00 | 3.33E+01 |
| U32 | 0.00E+00 | 8.73E+01 | 2.55E+04 | 4.63E+02 | 0.00E+00 | 4.59E+01 | 0.00E+00 | 2.70E+01 |
| U33 | 0.00E+00 | 1.20E+02 | 2.84E+04 | 6.07E+02 | 0.00E+00 | 5.52E+01 | 0.00E+00 | 3.61E+01 |
| U34 | 0.00E+00 | 9.85E+01 | 5.92E+04 | 5.92E+02 | 0.00E+00 | 4.46E+01 | 0.00E+00 | 2.62E+01 |
| U34A | 0.00E+00 | 8.78E+01 | 1.57E+04 | 3.51E+02 | 0.00E+00 | 4.27E+01 | 0.00E+00 | 2.48E+01 |
| U34B | 0.00E+00 | 9.78E+01 | 2.13E+04 | 4.90E+02 | 0.00E+00 | 5.11E+01 | 0.00E+00 | 3.15E+01 |
| U35 | 0.00E+00 | 7.14E+01 | 2.19E+03 | 1.06E+02 | 0.00E+00 | 3.86E+01 | 0.00E+00 | 2.34E+01 |
| U36 | 0.00E+00 | 1.19E+02 | 3.08E+04 | 6.27E+02 | 0.00E+00 | 5.58E+01 | 0.00E+00 | 3.55E+01 |
| U37 | 7.16E+02 | 1.10E+02 | 2.13E+03 | 1.33E+02 | 0.00E+00 | 4.96E+01 | 0.00E+00 | 3.02E+01 |
| U37S1 | 1.87E+02 | 9.50E+01 | 7.75E+02 | 1.04E+02 | 0.00E+00 | 6.33E+01 | 0.00E+00 | 3.79E+01 |
| CLS | 2.19E+03 | 1.92E+02 | 2.83E+03 | 1.78E+02 | 5.89E+01 | 6.72E+01 | 6.85E+01 | 4.23E+01 |
| CLN | 2.09E+03 | 1.35E+02 | 1.13E+04 | 2.59E+02 | 6.30E+01 | 3.39E+01 | 6.90E+01 | 2.19E+01 |

| Sample # | Zn | Zn Error | As | As Error | Rb | Rb Error | Sr | Sr Error |
| --- | --- | --- | --- | --- | --- | --- | --- | --- |
| U1S1 | 2.95E+01 | 1.27E+01 | 0.00E+00 | 7.23E+00 | 6.59E+00 | 1.46E+00 | 1.11E+02 | 6.56E+00 |
| U1S2 | 6.95E+01 | 1.23E+01 | 0.00E+00 | 6.33E+00 | 2.72E+01 | 2.01E+00 | 8.24E+01 | 4.52E+00 |
| U2 | 0.00E+00 | 1.83E+01 | 0.00E+00 | 8.60E+00 | 8.75E+00 | 1.71E+00 | 2.28E+02 | 9.78E+00 |
| U3 | 3.18E+01 | 1.28E+01 | 0.00E+00 | 8.20E+00 | 3.76E+01 | 2.78E+00 | 2.67E+02 | 9.53E+00 |
| U4 | 0.00E+00 | 1.49E+01 | 0.00E+00 | 6.43E+00 | 4.29E+00 | 1.24E+00 | 9.53E+01 | 5.80E+00 |
| U5 | 0.00E+00 | 2.37E+01 | 0.00E+00 | 1.05E+01 | 9.43E+00 | 2.06E+00 | 2.38E+02 | 1.18E+01 |
| U6 | 0.00E+00 | 1.46E+01 | 0.00E+00 | 5.81E+00 | 9.51E+00 | 1.52E+00 | 7.06E+01 | 4.92E+00 |
| U7 | 1.14E+02 | 2.31E+01 | 0.00E+00 | 9.77E+00 | 2.59E+01 | 2.96E+00 | 1.81E+02 | 9.79E+00 |
| U8 | 6.71E+01 | 1.94E+01 | 0.00E+00 | 2.32E+01 | 9.79E+01 | 5.51E+00 | 1.70E+02 | 9.48E+00 |
| U9 | 2.11E+01 | 1.08E+01 | 0.00E+00 | 6.76E+00 | 1.24E+01 | 1.60E+00 | 7.04E+01 | 4.68E+00 |
| U10 | 1.79E+01 | 1.03E+01 | 2.28E+01 | 9.49E+00 | 5.02E+00 | 1.14E+00 | 7.79E+01 | 4.66E+00 |
| U11 | 2.34E+01 | 1.06E+01 | 1.58E+01 | 1.03E+01 | 2.58E+01 | 2.14E+00 | 1.52E+02 | 6.59E+00 |
| U12 | 0.00E+00 | 2.49E+01 | 0.00E+00 | 1.07E+01 | 3.15E+00 | 1.54E+00 | 5.89E+01 | 6.22E+00 |
| U13 | 2.74E+01 | 1.58E+01 | 0.00E+00 | 1.07E+01 | 2.02E+01 | 2.59E+00 | 2.57E+02 | 1.14E+01 |
| U14 | 9.00E+01 | 1.65E+01 | 9.51E+00 | 5.94E+00 | 7.46E+01 | 3.70E+00 | 1.31E+02 | 6.49E+00 |
| U15 | 3.44E+01 | 1.53E+01 | 0.00E+00 | 8.93E+00 | 1.93E+01 | 2.44E+00 | 1.51E+02 | 8.56E+00 |
| U16 | 0.00E+00 | 1.30E+01 | 0.00E+00 | 6.80E+00 | 3.30E+00 | 1.05E+00 | 4.11E+01 | 3.63E+00 |
| U16-1 | 0.00E+00 | 1.40E+01 | 0.00E+00 | 6.99E+00 | 1.62E+00 | 1.00E+00 | 8.35E+01 | 5.24E+00 |
| U17A | 2.68E+01 | 1.05E+01 | 0.00E+00 | 1.41E+01 | 2.51E+01 | 1.99E+00 | 1.40E+02 | 5.98E+00 |
| U17B | 7.65E+01 | 1.93E+01 | 0.00E+00 | 9.84E+00 | 1.31E+01 | 2.13E+00 | 1.73E+02 | 9.02E+00 |
| U18A | 5.09E+01 | 1.51E+01 | 0.00E+00 | 9.93E+00 | 5.02E+01 | 3.42E+00 | 8.13E+01 | 5.83E+00 |
| U18B | 4.02E+01 | 1.43E+01 | 0.00E+00 | 1.77E+01 | 2.20E+01 | 2.32E+00 | 3.19E+02 | 1.08E+01 |
| U19 | 2.27E+01 | 1.31E+01 | 0.00E+00 | 8.44E+00 | 2.55E+00 | 1.35E+00 | 4.99E+02 | 1.44E+01 |
| U20 | 3.60E+01 | 1.40E+01 | 0.00E+00 | 9.32E+00 | 2.29E+01 | 2.28E+00 | 2.93E+02 | 1.02E+01 |
| U21A | 8.83E+01 | 2.19E+01 | 0.00E+00 | 1.23E+01 | 6.37E+00 | 1.81E+00 | 1.02E+02 | 7.67E+00 |
| U22 - JONS | 5.37E+01 | 1.37E+01 | 0.00E+00 | 8.07E+00 | 3.42E+01 | 2.66E+00 | 6.97E+01 | 5.01E+00 |
| U23 | 4.66E+01 | 1.72E+01 | 0.00E+00 | 9.75E+00 | 6.86E+00 | 1.72E+00 | 1.31E+02 | 8.12E+00 |
| U24 | 6.70E+01 | 1.54E+01 | 0.00E+00 | 1.55E+01 | 1.32E+01 | 1.76E+00 | 1.19E+02 | 6.27E+00 |
| U25 | 0.00E+00 | 2.25E+01 | 0.00E+00 | 9.74E+00 | 3.84E+00 | 1.58E+00 | 3.67E+02 | 1.40E+01 |
| U26 | 6.70E+01 | 2.07E+01 | 0.00E+00 | 1.07E+01 | 7.13E+00 | 1.95E+00 | 5.41E+02 | 1.74E+01 |
| U27 | 6.77E+01 | 1.57E+01 | 0.00E+00 | 9.12E+00 | 2.16E+01 | 2.17E+00 | 2.51E+02 | 9.06E+00 |
| U28 | 3.81E+01 | 1.43E+01 | 2.72E+01 | 1.36E+01 | 3.19E+01 | 2.87E+00 | 1.61E+02 | 8.24E+00 |
| U29 | 0.00E+00 | 1.46E+01 | 0.00E+00 | 6.03E+00 | 4.31E+00 | 1.18E+00 | 4.97E+01 | 4.11E+00 |
| U30 | 6.01E+01 | 2.20E+01 | 2.62E+01 | 1.51E+01 | 4.94E+01 | 3.49E+00 | 1.28E+02 | 6.52E+00 |
| U31 | 3.66E+01 | 1.47E+01 | 0.00E+00 | 9.93E+00 | 1.56E+01 | 2.21E+00 | 2.88E+02 | 1.11E+01 |
| U32 | 3.46E+01 | 1.16E+01 | 0.00E+00 | 1.52E+01 | 3.81E+01 | 2.51E+00 | 1.55E+02 | 6.52E+00 |
| U33 | 8.67E+01 | 1.84E+01 | 0.00E+00 | 1.20E+01 | 3.57E+01 | 3.04E+00 | 2.86E+02 | 1.08E+01 |
| U34 | 8.88E+01 | 1.36E+01 | 2.65E+01 | 9.96E+00 | 6.77E+01 | 2.67E+00 | 1.21E+02 | 4.15E+00 |
| U34A | 0.00E+00 | 1.33E+01 | 9.74E+00 | 5.10E+00 | 1.12E+01 | 1.46E+00 | 1.71E+02 | 6.57E+00 |
| U34B | 6.89E+01 | 1.61E+01 | 0.00E+00 | 9.71E+00 | 4.26E+01 | 3.08E+00 | 1.18E+02 | 6.71E+00 |
| U35 | 2.37E+01 | 9.79E+00 | 0.00E+00 | 5.61E+00 | 7.19E+00 | 1.20E+00 | 2.50E+01 | 2.80E+00 |
| U36 | 8.47E+01 | 1.81E+01 | 3.41E+01 | 1.89E+01 | 6.23E+01 | 3.92E+00 | 2.63E+02 | 1.03E+01 |
| U37 | 2.33E+01 | 1.20E+01 | 0.00E+00 | 6.86E+00 | 3.67E+00 | 1.23E+00 | 7.80E+01 | 5.47E+00 |
| U37S1 | 0.00E+00 | 2.02E+01 | 0.00E+00 | 8.86E+00 | 6.95E+00 | 3.33E+00 | 2.67E+02 | 1.14E+01 |
| CLS | 8.28E+01 | 2.00E+01 | 5.13E+01 | 1.78E+01 | 9.61E+00 | 2.10E+00 | 2.22E+03 | 3.12E+01 |
| CLN | 9.58E+01 | 1.49E+01 | 5.46E+01 | 1.38E+01 | 7.02E+00 | 1.32E+00 | 2.90E+03 | 2.49E+01 |

| Sample # | Zr | Zr Error | Nb | Nb Error | Mo | Mo Error | W | W Error |
| --- | --- | --- | --- | --- | --- | --- | --- | --- |
| U1S1 | 1.73E+01 | 4.58E+00 | 0.00E+00 | 2.93E+00 | 0.00E+00 | 6.19E+00 | 0.00E+00 | 5.99E+01 |
| U1S2 | 5.49E+01 | 4.45E+00 | 4.59E+00 | 2.28E+00 | 0.00E+00 | 5.17E+00 | 0.00E+00 | 4.41E+01 |
| U2 | 5.33E+01 | 6.68E+00 | 4.70E+00 | 2.98E+00 | 8.03E+00 | 4.91E+00 | 0.00E+00 | 8.29E+01 |
| U3 | 1.29E+02 | 7.79E+00 | 6.95E+00 | 2.77E+00 | 6.98E+00 | 3.30E+00 | 0.00E+00 | 6.40E+01 |
| U4 | 1.19E+02 | 6.86E+00 | 0.00E+00 | 3.94E+00 | 4.95E+00 | 3.22E+00 | 0.00E+00 | 5.92E+01 |
| U5 | 4.36E+01 | 7.58E+00 | 0.00E+00 | 6.72E+00 | 0.00E+00 | 8.26E+00 | 0.00E+00 | 8.47E+01 |
| U6 | 9.65E+01 | 6.08E+00 | 0.00E+00 | 3.79E+00 | 0.00E+00 | 6.17E+00 | 0.00E+00 | 5.65E+01 |
| U7 | 3.21E+02 | 1.31E+01 | 1.58E+01 | 3.85E+00 | 1.24E+01 | 4.62E+00 | 0.00E+00 | 8.67E+01 |
| U8 | 2.38E+02 | 1.16E+01 | 2.02E+01 | 4.01E+00 | 7.64E+00 | 4.34E+00 | 0.00E+00 | 8.39E+01 |
| U9 | 5.74E+01 | 4.93E+00 | 6.87E+00 | 2.53E+00 | 1.41E+01 | 4.15E+00 | 0.00E+00 | 5.82E+01 |
| U10 | 7.05E+01 | 5.00E+00 | 8.68E+00 | 2.48E+00 | 1.04E+01 | 2.91E+00 | 0.00E+00 | 5.74E+01 |
| U11 | 3.01E+02 | 9.25E+00 | 8.64E+00 | 2.58E+00 | 1.02E+01 | 3.28E+00 | 0.00E+00 | 5.22E+01 |
| U12 | 1.16E+02 | 8.85E+00 | 1.07E+01 | 3.82E+00 | 1.10E+01 | 4.47E+00 | 0.00E+00 | 1.02E+02 |
| U13 | 9.15E+01 | 8.56E+00 | 9.47E+00 | 3.47E+00 | 7.92E+00 | 3.98E+00 | 0.00E+00 | 8.46E+01 |
| U14 | 1.42E+02 | 7.25E+00 | 1.26E+01 | 2.84E+00 | 9.41E+00 | 3.25E+00 | 0.00E+00 | 6.76E+01 |
| U15 | 1.31E+02 | 8.73E+00 | 5.19E+00 | 3.17E+00 | 6.93E+00 | 3.87E+00 | 0.00E+00 | 7.53E+01 |
| U16 | 3.20E+02 | 9.01E+00 | 1.13E+01 | 2.64E+00 | 1.16E+01 | 3.28E+00 | 1.07E+02 | 7.03E+01 |
| U16-1 | 9.89E+01 | 6.13E+00 | 0.00E+00 | 4.64E+00 | 4.59E+00 | 3.04E+00 | 0.00E+00 | 5.53E+01 |
| U17A | 3.04E+02 | 8.72E+00 | 9.70E+00 | 2.46E+00 | 6.72E+00 | 3.02E+00 | 0.00E+00 | 5.33E+01 |
| U17B | 2.74E+02 | 1.16E+01 | 1.81E+01 | 3.71E+00 | 1.12E+01 | 4.24E+00 | 0.00E+00 | 8.42E+01 |
| U18A | 1.60E+02 | 8.26E+00 | 1.10E+01 | 3.14E+00 | 7.41E+00 | 3.62E+00 | 0.00E+00 | 6.58E+01 |
| U18B | 2.73E+02 | 1.06E+01 | 1.00E+01 | 3.01E+00 | 0.00E+00 | 5.35E+00 | 0.00E+00 | 6.97E+01 |
| U19 | 1.79E+02 | 1.04E+01 | 6.15E+00 | 3.09E+00 | 6.39E+00 | 3.78E+00 | 0.00E+00 | 6.82E+01 |
| U20 | 8.94E+01 | 7.24E+00 | 1.03E+01 | 2.94E+00 | 1.02E+01 | 3.41E+00 | 0.00E+00 | 7.40E+01 |
| U21A | 2.91E+02 | 1.26E+01 | 1.20E+01 | 3.76E+00 | 1.08E+01 | 4.59E+00 | 0.00E+00 | 9.07E+01 |
| U22 - JONS | 1.32E+02 | 7.02E+00 | 1.04E+01 | 2.91E+00 | 0.00E+00 | 6.67E+00 | 0.00E+00 | 5.48E+01 |
| U23 | 1.68E+02 | 9.62E+00 | 9.20E+00 | 3.43E+00 | 1.03E+01 | 4.15E+00 | 0.00E+00 | 8.29E+01 |
| U24 | 1.48E+02 | 7.41E+00 | 1.40E+01 | 2.93E+00 | 9.00E+00 | 3.30E+00 | 0.00E+00 | 1.17E+02 |
| U25 | 5.27E+01 | 8.24E+00 | 6.82E+00 | 3.46E+00 | 0.00E+00 | 5.96E+00 | 0.00E+00 | 8.69E+01 |
| U26 | 3.99E+01 | 9.03E+00 | 8.66E+00 | 3.65E+00 | 9.62E+00 | 4.22E+00 | 0.00E+00 | 9.39E+01 |
| U27 | 2.30E+02 | 9.27E+00 | 2.14E+01 | 3.23E+00 | 1.26E+01 | 3.56E+00 | 0.00E+00 | 1.22E+02 |
| U28 | 1.31E+02 | 8.21E+00 | 1.53E+01 | 3.38E+00 | 0.00E+00 | 7.42E+00 | 0.00E+00 | 6.64E+01 |
| U29 | 1.25E+02 | 6.42E+00 | 5.47E+00 | 2.57E+00 | 5.44E+00 | 3.06E+00 | 0.00E+00 | 5.42E+01 |
| U30 | 1.11E+02 | 7.00E+00 | 2.49E+01 | 3.74E+00 | 1.19E+01 | 3.85E+00 | 2.43E+02 | 1.07E+02 |
| U31 | 2.84E+02 | 1.16E+01 | 1.44E+01 | 3.46E+00 | 8.97E+00 | 4.06E+00 | 0.00E+00 | 7.08E+01 |
| U32 | 1.39E+02 | 6.74E+00 | 1.52E+01 | 2.73E+00 | 7.67E+00 | 2.99E+00 | 0.00E+00 | 5.77E+01 |
| U33 | 2.35E+02 | 1.06E+01 | 2.92E+01 | 3.87E+00 | 7.13E+00 | 3.83E+00 | 0.00E+00 | 7.15E+01 |
| U34 | 1.46E+02 | 5.08E+00 | 1.70E+01 | 2.25E+00 | 3.79E+00 | 2.37E+00 | 0.00E+00 | 5.21E+01 |
| U34A | 1.88E+02 | 7.27E+00 | 9.96E+00 | 2.49E+00 | 1.11E+01 | 3.01E+00 | 0.00E+00 | 5.16E+01 |
| U34B | 1.33E+02 | 7.61E+00 | 1.56E+01 | 3.20E+00 | 6.95E+00 | 3.45E+00 | 0.00E+00 | 6.62E+01 |
| U35 | 5.80E+02 | 1.09E+01 | 1.04E+01 | 2.41E+00 | 1.17E+01 | 3.27E+00 | 0.00E+00 | 5.08E+01 |
| U36 | 2.99E+02 | 1.15E+01 | 3.18E+01 | 3.93E+00 | 8.70E+00 | 3.95E+00 | 0.00E+00 | 6.89E+01 |
| U37 | 2.32E+02 | 9.12E+00 | 5.01E+00 | 2.75E+00 | 0.00E+00 | 6.97E+00 | 0.00E+00 | 6.09E+01 |
| U37S1 | 3.04E+01 | 6.70E+00 | 4.83E+00 | 3.17E+00 | 0.00E+00 | 7.61E+00 | 0.00E+00 | 7.75E+01 |
| CLS | 3.84E+01 | 1.34E+01 | 6.37E+01 | 5.06E+00 | 2.28E+01 | 4.20E+00 | 7.11E+01 | 8.73E+01 |
| CLN | 5.33E+01 | 7.85E+00 | 6.09E+01 | 3.20E+00 | 1.86E+01 | 2.65E+00 | 7.04E+01 | 4.30E+01 |

| Sample # | Pb | Pb Error | Bi | Bi Error | Th | Th Error | U | U Error |
| --- | --- | --- | --- | --- | --- | --- | --- | --- |
| U1S1 | 0.00E+00 | 8.12E+00 | 0.00E+00 | 6.62E+00 | 0.00E+00 | 5.34E+00 | 0.00E+00 | 8.37E+00 |
| U1S2 | 0.00E+00 | 7.59E+00 | 0.00E+00 | 1.05E+01 | 0.00E+00 | 5.42E+00 | 0.00E+00 | 8.84E+00 |
| U2 | 0.00E+00 | 1.12E+01 | 0.00E+00 | 1.05E+01 | 0.00E+00 | 6.66E+00 | 0.00E+00 | 1.10E+01 |
| U3 | 0.00E+00 | 9.99E+00 | 0.00E+00 | 1.07E+01 | 0.00E+00 | 6.90E+00 | 0.00E+00 | 1.25E+01 |
| U4 | 0.00E+00 | 7.31E+00 | 0.00E+00 | 6.53E+00 | 0.00E+00 | 5.31E+00 | 0.00E+00 | 8.04E+00 |
| U5 | 0.00E+00 | 1.35E+01 | 0.00E+00 | 9.78E+00 | 0.00E+00 | 7.43E+00 | 0.00E+00 | 1.27E+01 |
| U6 | 0.00E+00 | 7.48E+00 | 0.00E+00 | 6.53E+00 | 0.00E+00 | 5.08E+00 | 0.00E+00 | 8.36E+00 |
| U7 | 0.00E+00 | 1.23E+01 | 0.00E+00 | 1.37E+01 | 0.00E+00 | 8.84E+00 | 0.00E+00 | 1.41E+01 |
| U8 | 2.21E+01 | 1.02E+01 | 0.00E+00 | 2.15E+01 | 0.00E+00 | 1.08E+01 | 0.00E+00 | 2.13E+01 |
| U9 | 0.00E+00 | 8.14E+00 | 0.00E+00 | 1.03E+01 | 0.00E+00 | 5.59E+00 | 0.00E+00 | 8.55E+00 |
| U10 | 0.00E+00 | 7.98E+00 | 0.00E+00 | 5.89E+00 | 0.00E+00 | 4.61E+00 | 0.00E+00 | 7.32E+00 |
| U11 | 1.36E+01 | 6.47E+00 | 0.00E+00 | 7.31E+00 | 0.00E+00 | 5.59E+00 | 0.00E+00 | 1.02E+01 |
| U12 | 0.00E+00 | 1.26E+01 | 0.00E+00 | 1.53E+01 | 0.00E+00 | 8.17E+00 | 0.00E+00 | 1.15E+01 |
| U13 | 0.00E+00 | 1.24E+01 | 0.00E+00 | 1.84E+01 | 0.00E+00 | 8.35E+00 | 0.00E+00 | 1.30E+01 |
| U14 | 1.14E+01 | 6.62E+00 | 0.00E+00 | 1.49E+01 | 0.00E+00 | 7.32E+00 | 0.00E+00 | 1.43E+01 |
| U15 | 0.00E+00 | 1.12E+01 | 0.00E+00 | 1.38E+01 | 0.00E+00 | 7.24E+00 | 0.00E+00 | 1.19E+01 |
| U16 | 0.00E+00 | 8.19E+00 | 0.00E+00 | 7.81E+00 | 0.00E+00 | 5.31E+00 | 0.00E+00 | 7.33E+00 |
| U16-1 | 0.00E+00 | 8.57E+00 | 0.00E+00 | 6.17E+00 | 0.00E+00 | 5.46E+00 | 0.00E+00 | 7.63E+00 |
| U17A | 1.29E+01 | 6.00E+00 | 0.00E+00 | 7.89E+00 | 0.00E+00 | 5.19E+00 | 0.00E+00 | 9.19E+00 |
| U17B | 1.32E+01 | 8.22E+00 | 0.00E+00 | 1.43E+01 | 0.00E+00 | 7.84E+00 | 0.00E+00 | 1.16E+01 |
| U18A | 1.50E+01 | 7.76E+00 | 0.00E+00 | 1.70E+01 | 8.23E+00 | 5.33E+00 | 0.00E+00 | 1.35E+01 |
| U18B | 1.33E+01 | 7.41E+00 | 0.00E+00 | 1.07E+01 | 8.97E+00 | 5.06E+00 | 0.00E+00 | 1.19E+01 |
| U19 | 0.00E+00 | 9.96E+00 | 0.00E+00 | 8.10E+00 | 0.00E+00 | 6.52E+00 | 0.00E+00 | 1.10E+01 |
| U20 | 1.39E+01 | 7.43E+00 | 0.00E+00 | 1.33E+01 | 0.00E+00 | 6.94E+00 | 1.46E+01 | 8.33E+00 |
| U21A | 2.17E+01 | 1.03E+01 | 0.00E+00 | 1.25E+01 | 9.46E+00 | 6.28E+00 | 0.00E+00 | 1.14E+01 |
| U22 - JONS | 0.00E+00 | 8.95E+00 | 1.15E+01 | 7.19E+00 | 1.14E+01 | 4.92E+00 | 0.00E+00 | 1.16E+01 |
| U23 | 0.00E+00 | 1.19E+01 | 0.00E+00 | 8.79E+00 | 0.00E+00 | 7.40E+00 | 0.00E+00 | 1.08E+01 |
| U24 | 1.01E+01 | 6.47E+00 | 1.28E+01 | 6.80E+00 | 1.23E+01 | 5.03E+00 | 0.00E+00 | 9.21E+00 |
| U25 | 0.00E+00 | 1.15E+01 | 0.00E+00 | 8.63E+00 | 0.00E+00 | 6.94E+00 | 0.00E+00 | 1.20E+01 |
| U26 | 0.00E+00 | 1.19E+01 | 0.00E+00 | 1.74E+01 | 0.00E+00 | 8.63E+00 | 2.33E+01 | 1.08E+01 |
| U27 | 1.96E+01 | 7.74E+00 | 0.00E+00 | 1.03E+01 | 8.18E+00 | 4.82E+00 | 0.00E+00 | 1.12E+01 |
| U28 | 1.59E+01 | 8.19E+00 | 0.00E+00 | 1.35E+01 | 0.00E+00 | 7.50E+00 | 2.22E+01 | 9.68E+00 |
| U29 | 0.00E+00 | 7.72E+00 | 0.00E+00 | 7.08E+00 | 0.00E+00 | 5.19E+00 | 0.00E+00 | 7.27E+00 |
| U30 | 2.03E+01 | 1.01E+01 | 0.00E+00 | 1.51E+01 | 0.00E+00 | 3.00E+05 | 0.00E+00 | 3.00E+05 |
| U31 | 1.23E+01 | 7.89E+00 | 0.00E+00 | 1.17E+01 | 9.72E+00 | 5.45E+00 | 0.00E+00 | 1.23E+01 |
| U32 | 1.49E+01 | 6.38E+00 | 0.00E+00 | 1.40E+01 | 7.03E+00 | 4.27E+00 | 1.43E+01 | 7.67E+00 |
| U33 | 3.01E+01 | 9.78E+00 | 1.42E+01 | 8.61E+00 | 1.31E+01 | 6.07E+00 | 1.51E+01 | 9.77E+00 |
| U34 | 3.05E+01 | 7.26E+00 | 0.00E+00 | 1.27E+01 | 7.54E+00 | 4.45E+00 | 2.12E+01 | 8.58E+00 |
| U34A | 9.54E+00 | 5.53E+00 | 0.00E+00 | 5.95E+00 | 0.00E+00 | 4.92E+00 | 3.06E+01 | 8.15E+00 |
| U34B | 1.65E+01 | 7.72E+00 | 0.00E+00 | 1.71E+01 | 8.03E+00 | 5.08E+00 | 0.00E+00 | 1.34E+01 |
| U35 | 0.00E+00 | 7.22E+00 | 0.00E+00 | 7.92E+00 | 0.00E+00 | 4.72E+00 | 0.00E+00 | 6.96E+00 |
| U36 | 5.98E+01 | 1.25E+01 | 2.04E+01 | 9.89E+00 | 1.74E+01 | 6.79E+00 | 0.00E+00 | 1.66E+01 |
| U37 | 0.00E+00 | 8.36E+00 | 0.00E+00 | 1.02E+01 | 0.00E+00 | 6.02E+00 | 0.00E+00 | 8.24E+00 |
| U37S1 | 0.00E+00 | 9.31E+00 | 0.00E+00 | 1.06E+01 | 0.00E+00 | 7.06E+00 | 0.00E+00 | 9.84E+00 |
| CLS | 3.12E+01 | 1.11E+01 | 0.00E+00 | 9.53E+00 | 0.00E+00 | 8.14E+00 | 8.15E+01 | 1.58E+01 |
| CLN | 6.21E+01 | 9.08E+00 | 0.00E+00 | 7.97E+00 | 0.00E+00 | 6.64E+00 | 4.33E+01 | 1.08E+01 |

| Sample # | Bal | Bal Error |
| --- | --- | --- |
| U1S1 | 6.08E+05 | 3.33E+03 |
| U1S2 | 7.12E+05 | 2.26E+03 |
| U2 | 4.52E+05 | 4.51E+03 |
| U3 | 5.50E+05 | 3.62E+03 |
| U4 | 6.16E+05 | 3.22E+03 |
| U5 | 5.09E+05 | 5.03E+03 |
| U6 | 6.34E+05 | 3.02E+03 |
| U7 | 5.30E+05 | 4.73E+03 |
| U8 | 5.27E+05 | 4.72E+03 |
| U9 | 4.96E+05 | 3.47E+03 |
| U10 | 4.74E+05 | 3.34E+03 |
| U11 | 5.93E+05 | 3.10E+03 |
| U12 | 4.07E+05 | 5.06E+03 |
| U13 | 4.63E+05 | 5.06E+03 |
| U14 | 5.20E+05 | 3.67E+03 |
| U15 | 5.58E+05 | 4.16E+03 |
| U16 | 4.81E+05 | 3.32E+03 |
| U16-1 | 5.75E+05 | 3.29E+03 |
| U17A | 5.90E+05 | 2.97E+03 |
| U17B | 5.23E+05 | 4.43E+03 |
| U18A | 6.03E+05 | 3.67E+03 |
| U18B | 5.62E+05 | 3.79E+03 |
| U19 | 5.87E+05 | 3.86E+03 |
| U20 | 4.92E+05 | 4.00E+03 |
| U21A | 4.49E+05 | 4.81E+03 |
| U22 - JONS | 6.35E+05 | 3.31E+03 |
| U23 | 5.00E+05 | 4.47E+03 |
| U24 | 5.00E+05 | 3.78E+03 |
| U25 | 5.16E+05 | 4.66E+03 |
| U26 | 4.82E+05 | 4.99E+03 |
| U27 | 4.75E+05 | 4.04E+03 |
| U28 | 5.85E+05 | 3.80E+03 |
| U29 | 5.70E+05 | 3.45E+03 |
| U30 | 2.82E+05 | 4.65E+03 |
| U31 | 5.54E+05 | 4.41E+03 |
| U32 | 5.12E+05 | 3.44E+03 |
| U33 | 6.00E+05 | 3.87E+03 |
| U34 | 5.92E+05 | 2.54E+03 |
| U34A | 5.92E+05 | 2.95E+03 |
| U34B | 5.24E+05 | 3.98E+03 |
| U35 | 5.33E+05 | 3.18E+03 |
| U36 | 5.71E+05 | 4.15E+03 |
| U37 | 5.77E+05 | 3.47E+03 |
| U37S1 | 5.22E+05 | 4.37E+03 |
| CLS | 4.12E+05 | 4.89E+03 |
| CLN | 4.36E+05 | 3.11E+03 |

Table S2: CLDQ IBF Measurements and Data

| **Speciman** | **Size (length)** | **Size (thickness)** | **Shape** | **Abrasion** | **Weathering** |
| --- | --- | --- | --- | --- | --- |
| CLN-1 | 0.36 | 0.05 | Tabular | 2 | 2 |
| CLN-2 | 0.24 | 0.12 | Compact | 1 | 1 |
| CLN-3 | 0.18 | 0.12 | Compact | 2 | 1 |
| CLN-4 | 0.09 | 0.03 | Compact | 2 | 0 |
| CLN-5 | 0.19 | 0.05 | Tabular | 0 | 1 |
| CLN-6 | 0.13 | 0.04 | Compact | 0 | 1 |
| CLN-7 | 0.12 | 0.02 | Tabular | 2 | 1 |
| CLN-8 | 0.22 | 0.1 | Compact | 2 | 1 |
| CLN-9 | 0.12 | 0.05 | Compact | 3 | 1 |
| CLN-10 | 0.05 | 0.04 | Compact | 0 | 0 |
| CLN-11 | 0.14 | 0.03 | Tabular | 1 | 2 |
| CLN-12 | 0.18 | 0.08 | Compact | 0 | 1 |
| CLN-13 | 0.12 | 0.02 | Tabular | 1 | 0 |
| CLN-14 | 0.13 | 0.07 | Compact | 0 | 1 |
| CLN-15 | 0.41 | 0.06 | Tabular | 0 | 3 |
| CLN-16 | 0.21 | 0.08 | Conical | 3 | 0 |
| CLN-17 | 0.09 | 0.02 | Compact | 2 | 0 |
| CLN-18 | 0.1 | 0.06 | Compact | 0 | 1 |
| CLN-19 | 0.12 | 0.04 | Tabular | 3 | 3 |
| CLN-20 | 0.13 | 0.06 | Tabular | 1 | 1 |
| CLN-21 | 0.13 | 0.08 | Compact | 2 | 2 |
| CLN-22 | 0.31 | 0.07 | Tabular | 1 | 1 |
| CLN-23 | 0.08 | 0.02 | Tabular | 2 | 1 |
| CLN-24 | 0.25 | 0.05 | Compact | 2 | 3 |
| CLN-25 | 0.16 | 0.03 | Tabular | 0 | 2 |
| CLN-26 | 0.1 | 0.07 | Compact | 1 | 2 |
| CLN-27 | 0.3 | 0.05 | Tabular | 2 | 3 |
| CLN-28 | 0.06 | 0.05 | Compact | 1 | 1 |
| CLN-29 | 0.09 | 0.04 | Compact | 1 | 0 |
| CLN-30 | 0.26 | 0.06 | Elongate | 3 | 2 |
| CLN-31 | 0.19 | 0.04 | Elongate | 1 | 2 |
| CLN-32 | 0.12 | 0.03 | Compact | 0 | 2 |
| CLN-33 | 0.12 | 0.06 | Compact | 3 | 3 |
| CLN-34 | 0.19 | 0.06 | Elongate | 1 | 2 |
| CLN-35 | 0.16 | 0.04 | Elongate | 3 | 2 |
| CLN-36 | 0.16 | 0.07 | Compact | 1 | 2 |
| CLN-37 | 0.06 | 0.05 | Compact | 3 | 2 |
| CLN-38 | 0.05 | 0.05 | Compact | 0 | 1 |
| CLN-39 | 0.14 | 0.04 | Compact | 1 | 3 |
| CLN-40 | 0.15 | 0.06 | Compact | 2 | 2 |
| CLN-41 | 0.1 | 0.05 | Compact | 1 | 1 |
| CLN-42 | 0.17 | 0.03 | Compact | 0 | 0 |
| CLN-43 | 0.25 | 0.07 | Compact | 0 | 0 |
| CLN-44 | 0.09 | 0.04 | Compact | 2 | 2 |
| CLN-45 | 0.19 | 0.1 | Compact | 3 | 3 |
| CLN-46 | 0.12 | 0.09 | Compact | 3 | 3 |
| CLN-47 | 0.33 | 0.04 | Elongate | 2 | 1 |
| CLN-48 | 0.28 | 0.06 | Elongate | 0 | 1 |
| CLN-49 | 0.15 | 0.04 | Compact | 0 | 1 |
| CLN-50 | 0.08 | 0.03 | Compact | 0 | 1 |
| CLN-51 | 0.05 | 0.01 | Tabular | 0 | 1 |
| CLN-52 | 0.06 | 0.03 | Compact | 0 | 2 |
| CLN-53 | 0.22 | 0.19 | Compact | 2 | 3 |
| CLN-54 | 0.41 | 0.11 | Compact | 1 | 2 |
| CLN-55 | 0.26 | 0.16 | Compact | 1 | 2 |
| CLN-56 | 0.45 | 0.11 | Elongate | 3 | 3 |
| CLN-57 | 0.22 | 0.08 | Elongate | 3 | 2 |
| CLN-58 | 0.11 | 0.03 | Compact | 3 | 3 |
| CLN-59 | 0.14 | 0.05 | Compact | 2 | 1 |
| CLN-60 | 0.27 | 0.11 | Compact | 2 | 3 |
| CLN-61 | 0.09 | 0.07 | Compact | 0 | 2 |
| CLN-62 | 0.1 | 0.07 | Compact | 2 | 2 |
| CLN-63 | 0.09 | 0.03 | Tabular | 0 | 1 |
| CLN-64 | 0.16 | 0.06 | Compact | 0 | 1 |
| CLN-65 | 0.12 | 0.07 | Compact | 3 | 3 |
| CLN-66 | 0.07 | 0.03 | Tabular | 1 | 1 |
| CLN-67 | 0.07 | 0.03 | Compact | 3 | 2 |
| CLN-68 | 0.25 | 0.02 | Tabular | 1 | 3 |
| CLN-69 | 0.22 | 0.1 | Compact | 3 | 3 |
| CLN-70 | 0.18 | 0.05 | Compact | 2 | 3 |
| CLN-71 | 0.12 | 0.06 | Compact | 1 | 1 |
| CLN-72 | 0.07 | 0.05 | Compact | 3 | 3 |
| CLN-73 | 0.25 | 0.12 | Compact | 3 | 3 |
| CLN-74 | 0.09 | 0.05 | Compact | 1 | 2 |
| CLN-75 | 0.12 | 0.04 | Compact | 0 | 1 |
| CLN-76 | 0.1 | 0.06 | Compact | 2 | 2 |
| CLN-77 | 0.12 | 0.05 | Compact | 2 | 3 |
| CLN-78 | 0.1 | 0.07 | Compact | 3 | 3 |
| CLN-79 | 0.12 | 0.04 | Compact | 1 | 2 |
| CLN-80 | 0.17 | 0.06 | Compact | 0 | 3 |
| CLN-81 | 0.26 | 0.07 | Compact | 3 | 3 |
| CLN-82 | 0.2 | 0.09 | Compact | 3 | 3 |
| CLN-83 | 0.16 | 0.12 | Compact | 3 | 3 |
| CLN-84 | 0.17 | 0.09 | Compact | 1 | 3 |
| CLN-85 | 0.08 | 0.05 | Compact | 1 | 1 |
| CLN-86 | 0.06 | 0.05 | Compact | 2 | 2 |
| CLN-87 | 0.19 | 0.09 | Compact | 2 | 3 |
| CLN-88 | 0.11 | 0.07 | Compact | 2 | 2 |
| CLN-89 | 0.12 | 0.06 | Compact | 2 | 0 |
| CLN-90 | 0.13 | 0.07 | Compact | 3 | 2 |
| CLN-91 | 0.17 | 0.08 | Compact | 3 | 3 |
| CLN-92 | 0.09 | 0.04 | Tabular | 1 | 1 |
| CLN-93 | 0.09 | 0.05 | Compact | 2 | 0 |
| CLN-94 | 0.17 | 0.03 | Tabular | 2 | 1 |
| CLN-95 | 0.25 | 0.08 | Compact | 3 | 3 |
| CLN-96 | 0.05 | 0.05 | Compact | 2 | 1 |
| CLN-97 | 0.14 | 0.04 | Tabular | 3 | 3 |
| CLN-98 | 0.11 | 0.06 | Compact | 0 | 3 |
| CLN-99 | 0.09 | 0.06 | Compact | 1 | 3 |
| CLN-100 | 0.18 | 0.07 | Compact | 2 | 3 |
| CLN-101 | 0.13 | 0.05 | Compact | 3 | 3 |
| CLN-102 | 0.16 | 0.1 | Compact | 3 | 3 |
| CLN-103 | 0.09 | 0.05 | Compact | 1 | 0 |
| CLN-104 | 0.11 | 0.03 | Tabular | 2 | 2 |
| CLN-105 | 0.18 | 0.08 | Tabular | 1 | 2 |
| CLN-106 | 0.08 | 0.04 | Compact | 2 | 3 |
| CLN-107 | 0.09 | 0.01 | Tabular | 0 | 0 |
| CLN-108 | 0.13 | 0.05 | Compact | 3 | 1 |
| CLN-109 | 0.08 | 0.05 | Compact | 0 | 1 |
| CLN-110 | 0.21 | 0.02 | Elongate | 0 | 1 |
| CLN-111 | 0.15 | 0.05 | Elongate | 2 | 2 |
| CLN-112 | 0.09 | 0.08 | Compact | 3 | 3 |
| CLN-113 | 0.14 | 0.06 | Compact | 2 | 3 |
| CLN-114 | 0.1 | 0.04 | Compact | 2 | 3 |
| CLN-115 | 0.12 | 0.04 | Compact | 3 | 3 |
| CLN-116 | 0.13 | 0.07 | Compact | 2 | 3 |
| CLN-117 | 0.16 | 0.06 | Compact | 1 | 3 |
| CLN-118 | 0.1 | 0.06 | Compact | 2 | 3 |
| CLN-119 | 0.1 | 0.06 | Compact | 1 | 2 |
| CLN-120 | 0.12 | 0.04 | Compact | 0 | 1 |
| CLN-121 | 0.13 | 0.03 | Compact | 1 | 2 |
| CLN-122 | 0.08 | 0.02 | Compact | 1 | 2 |
| CLN-123 | 0.11 | 0.03 | Tabular | 1 | 1 |
| CLN-124 | 0.14 | 0.07 | Compact | 2 | 3 |
| CLN-125 | 0.11 | 0.01 | Tabular | 1 | 0 |
| CLN-126 | 0.09 | 0.04 | Compact | 3 | 3 |
| CLN-127 | 0.13 | 0.06 | Compact | 0 | 3 |
| CLN-128 | 0.11 | 0.06 | Compact | 1 | 1 |
| CLN-129 | 0.17 | 0.04 | Compact | 0 | 3 |
| CLN-130 | 0.1 | 0.05 | Compact | 0 | 3 |
| CLN-131 | 0.1 | 0.02 | Tabular | 2 | 2 |
| CLN-132 | 0.13 | 0.03 | Tabular | 3 | 3 |
| CLN-133 | 0.1 | 0.06 | Compact | 0 | 2 |
| CLN-134 | 0.16 | 0.03 | Tabular | 2 | 3 |
| CLN-135 | 0.08 | 0.06 | Compact | 2 | 1 |
| CLN-136 | 0.09 | 0.03 | Compact | 1 | 3 |
| CLN-137 | 0.1 | 0.04 | Compact | 3 | 3 |
| CLN-138 | 0.09 | 0.07 | Compact | 1 | 2 |
| CLN-139 | 0.11 | 0.06 | Compact | 1 | 3 |
| CLN-140 | 0.2 | 0.02 | Elongate | 0 | 2 |
| CLN-141 | 0.06 | 0.05 | Compact | 3 | 3 |
| CLN-142 | 0.1 | 0.04 | Compact | 0 | 1 |
| CLN-143 | 0.09 | 0.04 | Compact | 0 | 2 |
| CLN-144 | 0.08 | 0.03 | Tabular | 0 | 1 |
| CLN-145 | 0.11 | 0.02 | Tabular | 2 | 0 |
| CLN-146 | 0.06 | 0.06 | Compact | 3 | 3 |
| CLN-147 | 0.12 | 0.06 | Compact | 2 | 3 |
| CLN-148 | 0.07 | 0.04 | Compact | 0 | 2 |
| CLN-149 | 0.11 | 0.04 | Compact | 2 | 3 |
| CLN-150 | 0.07 | 0.05 | Compact | 0 | 2 |
| CLN-151 | 0.04 | 0.03 | Compact | 0 | 3 |
| CLN-152 | 0.12 | 0.04 | Compact | 0 | 3 |
| CLN-153 | 0.09 | 0.06 | Compact | 1 | 2 |
| CLN-154 | 0.06 | 0.04 | Compact | 3 | 3 |
| CLN-155 | 0.11 | 0.02 | Tabular | 0 | 2 |
| CLN-156 | 0.03 | 0.03 | Compact | 3 | 3 |
| CLN-157 | 0.11 | 0.03 | Tabular | 2 | 3 |
| CLN-158 | 0.06 | 0.03 | Compact | 3 | 3 |
| CLN-159 | 0.09 | 0.03 | Tabular | 0 | 3 |
| CLN-160 | 0.09 | 0.05 | Compact | 1 | 2 |
| CLN-161 | 0.06 | 0.05 | Compact | 3 | 3 |
| CLN-162 | 0.07 | 0.05 | Compact | 0 | 2 |
| CLN-163 | 0.04 | 0.02 | Compact | 3 | 3 |
| CLN-164 | 0.08 | 0.03 | Compact | 2 | 3 |
| CLN-165 | 0.1 | 0.05 | Compact | 1 | 3 |
| CLN-166 | 0.12 | 0.02 | Tabular | 0 | 2 |
| CLN-167 | 0.13 | 0.04 | Compact | 2 | 1 |
| CLN-168 | 0.05 | 0.05 | Compact | 2 | 3 |
| CLN-169 | 0.08 | 0.03 | Compact | 0 | 3 |
| CLN-170 | 0.07 | 0.04 | Compact | 2 | 2 |
| CLN-171 | 0.06 | 0.05 | Compact | 3 | 3 |
| CLN-172 | 0.08 | 0.04 | Compact | 2 | 2 |
| CLN-173 | 0.09 | 0.04 | Elongate | 2 | 1 |
| CLN-174 | 0.08 | 0.05 | Compact | 1 | 3 |
| CLN-175 | 0.09 | 0.04 | Compact | 0 | 3 |
| CLN-176 | 0.07 | 0.06 | Compact | 2 | 3 |
| CLN-177 | 0.08 | 0.04 | Compact | 1 | 2 |
| CLN-178 | 0.08 | 0.03 | Tabular | 0 | 2 |
| CLN-179 | 0.09 | 0.04 | Compact | 2 | 1 |
| CLN-180 | 0.07 | 0.03 | Tabular | 0 | 2 |
| CLN-181 | 0.07 | 0.05 | Tabular | 1 | 1 |
| CLN-182 | 0.08 | 0.04 | Compact | 3 | 3 |
| CLN-183 | 0.08 | 0.04 | Tabular | 1 | 2 |
| CLN-184 | 0.14 | 0.03 | Elongate | 2 | 1 |
| CLN-185 | 0.07 | 0.05 | Compact | 2 | 2 |
| CLN-186 | 0.08 | 0.02 | Tabular | 2 | 3 |
| CLN-187 | 0.07 | 0.03 | Compact | 1 | 1 |
| CLN-188 | 0.1 | 0.02 | Tabular | 3 | 3 |
| CLN-189 | 0.09 | 0.04 | Tabular | 1 | 2 |
| CLN-190 | 0.1 | 0.03 | Elongate | 3 | 2 |
| CLN-191 | 0.1 | 0.05 | Compact | 1 | 1 |
| CLN-192 | 0.06 | 0.04 | Compact | 1 | 1 |
| CLN-193 | 0.06 | 0.04 | Compact | 2 | 2 |
| CLN-194 | 0.13 | 0.03 | Tabular | 3 | 1 |
| CLN-195 | 0.06 | 0.05 | Compact | 2 | 3 |
| CLN-196 | 0.14 | 0.04 | Tabular | 3 | 3 |
| CLN-197 | 0.1 | 0.03 | Tabular | 2 | 0 |
| CLN-198 | 0.05 | 0.03 | Compact | 2 | 2 |
| CLN-199 | 0.07 | 0.05 | Tabular | 3 | 1 |
| CLN-200 | 0.07 | 0.05 | Compact | 1 | 1 |
| CLN-201 | 0.08 | 0.05 | Compact | 3 | 3 |
| CLN-202 | 0.11 | 0.02 | Tabular | 3 | 3 |
| CLN-203 | 0.09 | 0.04 | Compact | 2 | 3 |
| CLN-204 | 0.07 | 0.03 | Compact | 0 | 0 |
| CLN-205 | 0.1 | 0.01 | Tabular | 0 | 2 |
| CLN-206 | 0.06 | 0.03 | Compact | 0 | 1 |
| CLN-207 | 0.08 | 0.04 | Compact | 2 | 2 |
| CLN-208 | 0.08 | 0.03 | Compact | 2 | 3 |
| CLN-209 | 0.06 | 0.05 | Compact | 0 | 1 |
| CLN-210 | 0.08 | 0.03 | Compact | 3 | 2 |
| CLN-211 | 0.07 | 0.03 | Tabular | 3 | 2 |
| CLN-212 | 0.13 | 0.04 | Compact | 0 | 3 |
| CLN-213 | 0.06 | 0.04 | Compact | 3 | 3 |
| CLN-214 | 0.06 | 0.04 | Compact | 1 | 0 |
| CLN-215 | 0.1 | 0.03 | Tabular | 1 | 1 |
| CLN-216 | 0.1 | 0.02 | Tabular | 1 | 3 |
| CLN-217 | 0.11 | 0.03 | Elongate | 3 | 3 |
| CLN-218 | 0.13 | 0.02 | Tabular | 2 | 2 |
| CLN-219 | 0.06 | 0.04 | Compact | 3 | 3 |
| CLN-220 | 0.08 | 0.03 | Compact | 2 | 3 |
| CLN-221 | 0.09 | 0.03 | Compact | 0 | 2 |
| CLN-222 | 0.07 | 0.03 | Tabular | 3 | 2 |
| CLN-223 | 0.08 | 0.03 | Compact | 2 | 3 |
| CLN-224 | 0.09 | 0.02 | Tabular | 2 | 0 |
| CLN-225 | 0.1 | 0.02 | Elongate | 3 | 1 |
| CLN-226 | 0.07 | 0.02 | Compact | 3 | 2 |
| CLN-227 | 0.08 | 0.02 | Compact | 0 | 1 |
| CLN-228 | 0.05 | 0.04 | Compact | 0 | 1 |
| CLN-229 | 0.06 | 0.04 | Compact | 2 | 3 |
| CLN-230 | 0.08 | 0.02 | Compact | 3 | 1 |
| CLN-231 | 0.06 | 0.04 | Compact | 3 | 3 |
| CLN-232 | 0.08 | 0.04 | Compact | 1 | 3 |
| CLN-233 | 0.09 | 0.04 | Compact | 0 | 3 |
| CLN-234 | 0.1 | 0.03 | Compact | 3 | 3 |
| CLN-235 | 0.07 | 0.03 | Compact | 1 | 2 |
| CLN-236 | 0.08 | 0.03 | Compact | 1 | 3 |
| CLN-237 | 0.07 | 0.03 | Tabular | 1 | 2 |
| CLN-238 | 0.07 | 0.02 | Tabular | 0 | 2 |
| CLN-239 | 0.1 | 0.03 | Compact | 2 | 3 |
| CLN-240 | 0.06 | 0.03 | Compact | 3 | 2 |
| CLN-241 | 0.05 | 0.04 | Compact | 3 | 2 |
| CLN-242 | 0.07 | 0.04 | Compact | 3 | 3 |
| CLN-243 | 0.06 | 0.02 | Tabular | 1 | 1 |
| CLN-244 | 0.06 | 0.02 | Compact | 2 | 1 |
| CLN-245 | 0.05 | 0.02 | Tabular | 0 | 2 |
| CLN-246 | 0.06 | 0.02 | Compact | 1 | 2 |
| CLN-247 | 0.09 | 0.02 | Tabular | 2 | 1 |
| CLN-248 | 0.05 | 0.01 | Tabular | 2 | 1 |
| CLN-249 | 0.07 | 0.01 | Tabular | 3 | 2 |
| CLN-250 | 0.07 | 0.02 | Compact | 1 | 1 |
| CLN-251 | 0.47 | 0.29 | Compact | 3 | 3 |
| CLN-252 | 0.38 | 0.16 | Compact | 3 | 3 |
| CLN-253 | 0.64 | 0.21 | Elongate | 2 | 3 |
| CLN-254 | 0.52 | 0.24 | Compact | 1 | 1 |
| CLN-255 | 0.63 | 0.18 | Elongate | 2 | 3 |
| CLN-256 | 0.29 | 0.13 | Compact | 3 | 2 |
| CLS-1 | 0.08 | 0.01 | Tabular | 0 | 0 |
| CLS-2 | 0.15 | 0.01 | Tabular | 0 | 0 |
| CLS-3 | 0.07 | 0.01 | Tabular | 1 | 0 |
| CLS-4 | 0.13 | 0.01 | Tabular | 0 | 0 |
| CLS-5 | 0.18 | 0.01 | Tabular | 1 | 0 |
| CLS-6 | 0.11 | 0.01 | Tabular | 0 | 0 |
| CLS-7 | 0.06 | 0.08 | Compact | 2 | 2 |
| CLS-8 | 0.12 | 0.04 | Tabular | 1 | 1 |
| CLS-9 | 0.19 | 0.02 | Tabular | 0 | 1 |
| CLS-10 | 0.09 | 0.01 | Tabular | 0 | 0 |
| CLS-11 | 0.13 | 0.01 | Tabular | 1 | 1 |
| CLS-12 | 0.07 | 0.01 | Tabular | 0 | 0 |
| CLS-13 | 0.12 | 0.06 | Compact | 1 | 2 |
| CLS-14 | 0.07 | 0.05 | Compact | 2 | 1 |
| CLS-15 | 0.25 | 0.02 | Tabular | 0 | 0 |
| CLS-16 | 0.11 | 0.05 | Elongate | 0 | 1 |
| CLS-17 | 0.35 | 0.13 | Compact | 0 | 3 |
| CLS-18 | 0.13 | 0.01 | Tabular | 0 | 0 |
| CLS-19 | 0.1 | 0.08 | Compact | 3 | 2 |
| CLS-20 | 0.21 | 0.06 | Elongate | 0 | 3 |
| CLS-21 | 0.2 | 0.08 | Tabular | 1 | 1 |
| CLS-22 | 0.32 | 0.02 | Tabular | 2 | 1 |
| CLS-23 | 0.09 | 0.04 | Tabular | 0 | 0 |
| CLS-24 | 0.31 | 0.03 | Elongate | 1 | 1 |
| CLS-25 | 0.07 | 0.01 | Tabular | 1 | 2 |
| CLS-26 | 0.32 | 0.08 | compact | 0 | 3 |
| CLS-27 | 0.08 | 0.01 | Tabular | 0 | 0 |
| CLS-28 | 0.22 | 0.05 | Tabular | 1 | 2 |
| CLS-29 | 0.1 | 0.02 | Tabular | 0 | 1 |
| CLS-30 | 0.12 | 0.02 | Tabular | 1 | 1 |
| CLS-31 | 0.11 | 0.05 | Tabular | 2 | 2 |
| CLS-32 | 0.08 | 0.01 | Tabular | 1 | 1 |
| CLS-33 | 0.12 | 0.02 | Tabular | 1 | 1 |
| CLS-34 | 0.14 | 0.02 | Tabular | 1 | 1 |
| CLS-35 | 0.14 | 0.01 | Tabular | 2 | 1 |
| CLS-36 | 0.2 | 0.05 | Tabular | 0 | 3 |
| CLS-37 | 0.23 | 0.17 | Compact | 3 | 3 |
| CLS-38 | 0.07 | 0.02 | Tabular | 0 | 2 |
| CLS-39 | 0.19 | 0.02 | Tabular | 0 | 1 |
| CLS-40 | 0.11 | 0.01 | Tabular | 2 | 2 |
| CLS-41 | 0.13 | 0.05 | Tabular | 1 | 1 |
| CLS-42 | 0.06 | 0.01 | Tabular | 0 | 1 |
| CLS-43 | 0.12 | 0.01 | Tabular | 2 | 2 |
| CLS-44 | 0.08 | 0.02 | Compact | 2 | 3 |
| CLS-45 | 0.2 | 0.07 | Tabular | 3 | 3 |
| CLS-46 | 0.17 | 0.04 | Elongate | 0 | 2 |
| CLS-47 | 0.16 | 0.06 | Compact | 1 | 3 |
| CLS-48 | 0.31 | 0.11 | Tabular | 2 | 2 |
| CLS-49 | 0.17 | 0.02 | Tabular | 1 | 1 |
| CLS-50 | 0.19 | 0.03 | Tabular | 0 | 0 |
| CLS-51 | 0.14 | 0.02 | Tabular | 2 | 1 |
| CLS-52 | 0.19 | 0.05 | Tabular | 3 | 1 |
| CLS-53 | 0.11 | 0.04 | Compact | 1 | 1 |
| CLS-54 | 0.29 | 0.03 | Elongate | 2 | 0 |
| CLS-55 | 0.1 | 0.02 | Tabular | 1 | 2 |
| CLS-56 | 0.27 | 0.08 | Compact | 1 | 3 |
| CLS-57 | 0.14 | 0.13 | Compact | 2 | 2 |
| CLS-58 | 0.29 | 0.08 | Compact | 1 | 0 |
| CLS-59 | 0.24 | 0.04 | Tabular | 0 | 2 |
| CLS-60 | 0.12 | 0.04 | Tabular | 1 | 2 |
| CLS-61 | 0.36 | 0.11 | Elongate | 2 | 0 |
| CLS-62 | 0.17 | 0.02 | Elongate | 0 | 1 |
| CLS-63 | 0.09 | 0.06 | Compact | 0 | 3 |
| CLS-64 | 0.29 | 0.06 | Elongate | 2 | 3 |
| CLS-65 | 0.31 | 0.02 | Tabular | 1 | 1 |
| CLS-66 | 0.19 | 0.05 | Compact | 0 | 2 |
| CLS-67 | 0.17 | 0.03 | Tabular | 1 | 2 |
| CLS-68 | 0.19 | 0.06 | Compact | 3 | 3 |
| CLS-69 | 0.21 | 0.08 | Elongate | 1 | 3 |
| CLS-70 | 0.17 | 0.03 | Tabular | 1 | 1 |
| CLS-71 | 0.3 | 0.11 | Compact | 3 | 1 |
| CLS-72 | 0.13 | 0.05 | Compact | 1 | 2 |
| CLS-73 | 0.24 | 0.05 | Tabular | 2 | 3 |
| CLS-74 | 0.17 | 0.05 | Compact | 3 | 0 |
| CLS-75 | 0.1 | 0.04 | Tabular | 0 | 2 |
| CLS-76 | 0.11 | 0.03 | Tabular | 2 | 1 |
| CLS-77 | 0.1 | 0.06 | Compact | 0 | 0 |
| CLS-78 | 0.18 | 0.06 | Compact | 3 | 3 |
| CLS-79 | 0.2 | 0.02 | Elongate | 0 | 2 |
| CLS-80 | 0.23 | 0.06 | Compact | 1 | 2 |
| CLS-81 | 0.28 | 0.08 | Compact | 1 | 2 |
| CLS-82 | 0.09 | 0.02 | Tabular | 1 | 1 |
| CLS-83 | 0.07 | 0.02 | Tabular | 0 | 0 |
| CLS-84 | 0.13 | 0.06 | Compact | 2 | 1 |
| CLS-85 | 0.17 | 0.08 | Compact | 3 | 2 |
| CLS-86 | 0.12 | 0.1 | Compact | 3 | 1 |
| CLS-87 | 0.28 | 0.03 | Tabular | 0 | 2 |
| CLS-88 | 0.18 | 0.01 | Tabular | 0 | 0 |
| CLS-89 | 0.3 | 0.02 | Elongate | 1 | 1 |
| CLS-90 | 0.23 | 0.09 | Compact | 1 | 2 |
| CLS-91 | 0.12 | 0.02 | Tabular | 2 | 2 |
| CLS-92 | 0.09 | 0.07 | Compact | 2 | 1 |
| CLS-93 | 0.28 | 0.07 | Compact | 1 | 3 |
| CLS-94 | 0.08 | 0.01 | Tabular | 1 | 0 |
| CLS-95 | 0.1 | 0.04 | Compact | 2 | 1 |
| CLS-96 | 0.11 | 0.02 | Tabular | 1 | 0 |
| CLS-97 | 0.17 | 0.02 | Tabular | 2 | 1 |
| CLS-98 | 0.11 | 0.04 | Compact | 1 | 2 |
| CLS-99 | 0.14 | 0.06 | Compact | 0 | 3 |
| CLS-100 | 0.41 | 0.11 | Compact | 1 | 1 |
| CLS-101 | 0.1 | 0.08 | Compact | 0 | 3 |
| CLS-102 | 0.15 | 0.06 | Compact | 1 | 0 |
| CLS-103 | 0.08 | 0.02 | Tabular | 1 | 0 |
| CLS-104 | 0.2 | 0.02 | Tabular | 0 | 0 |
| CLS-105 | 0.22 | 0.03 | Tabular | 0 | 3 |
| CLS-106 | 0.11 | 0.06 | Compact | 3 | 3 |
| CLS-107 | 0.06 | 0.04 | Compact | 3 | 0 |
| CLS-108 | 0.08 | 0.01 | Tabular | 2 | 0 |
| CLS-109 | 0.17 | 0.03 | Tabular | 2 | 0 |
| CLS-110 | 0.33 | 0.06 | Tabular | 1 | 3 |
| CLS-111 | 0.21 | 0.07 | Tabular | 1 | 1 |
| CLS-112 | 0.19 | 0.03 | Elongate | 2 | 1 |
| CLS-113 | 0.32 | 0.06 | Tabular | 3 | 3 |
| CLS-114 | 0.06 | 0.05 | Elongate | 0 | 0 |
| CLS-115 | 0.08 | 0.01 | Tabular | 3 | 1 |
| CLS-116 | 0.07 | 0.05 | Compact | 3 | 2 |
| CLS-117 | 0.14 | 0.09 | Compact | 1 | 0 |
| CLS-118 | 0.13 | 0.06 | Compact | 1 | 1 |
| CLS-119 | 0.12 | 0.06 | Compact | 1 | 0 |
| CLS-120 | 0.13 | 0.01 | Tabular | 2 | 1 |
| CLS-121 | 0.11 | 0.02 | Tabular | 3 | 0 |
| CLS-122 | 0.12 | 0.03 | Elongate | 1 | 2 |
| CLS-123 | 0.17 | 0.04 | Elongate | 0 | 1 |
| CLS-124 | 0.1 | 0.02 | Tabular | 2 | 1 |
| CLS-125 | 0.2 | 0.02 | Tabular | 2 | 1 |
| CLS-126 | 0.29 | 0.14 | Compact | 2 | 2 |
| CLS-127 | 0.06 | 0.05 | Compact | 3 | 1 |
| CLS-128 | 0.13 | 0.05 | Compact | 2 | 1 |
| CLS-129 | 0.15 | 0.01 | Tabular | 1 | 1 |
| CLS-130 | 0.21 | 0.02 | Tabular | 1 | 0 |
| CLS-131 | 0.14 | 0.04 | Compact | 1 | 2 |
| CLS-132 | 0.1 | 0.06 | Compact | 0 | 0 |
| CLS-133 | 0.09 | 0.05 | Compact | 1 | 1 |
| CLS-134 | 0.11 | 0.05 | Compact | 1 | 2 |
| CLS-135 | 0.4 | 0.06 | Elongate | 1 | 2 |
| CLS-136 | 0.11 | 0.05 | Compact | 1 | 2 |
| CLS-137 | 0.1 | 0.02 | Tabular | 1 | 3 |
| CLS-138 | 0.37 | 0.07 | Tabular | 3 | 2 |
| CLS-139 | 0.21 | 0.03 | Tabular | 0 | 0 |
| CLS-140 | 0.08 | 0.07 | Compact | 0 | 2 |
| CLS-141 | 0.16 | 0.02 | Tabular | 1 | 0 |
| CLS-142 | 0.08 | 0.06 | Compact | 1 | 2 |
| CLS-143 | 0.34 | 0.07 | Tabular | 2 | 2 |
| CLS-144 | 0.14 | 0.02 | Tabular | 1 | 1 |
| CLS-145 | 0.2 | 0.09 | Compact | 2 | 3 |
| CLS-146 | 0.21 | 0.03 | Tabular | 1 | 2 |
| CLS-147 | 0.21 | 0.03 | Compact | 2 | 1 |
| CLS-148 | 0.21 | 0.03 | Tabular | 2 | 1 |
| CLS-149 | 0.11 | 0.02 | Tabular | 0 | 1 |
| CLS-150 | 0.11 | 0.03 | Compact | 3 | 2 |
| CLS-151 | 0.15 | 0.05 | Compact | 3 | 3 |
| CLS-152 | 0.2 | 0.09 | Compact | 1 | 1 |
| CLS-153 | 0.07 | 0.06 | Compact | 0 | 0 |
| CLS-154 | 0.21 | 0.05 | Compact | 1 | 2 |
| CLS-155 | 0.11 | 0.02 | Elongate | 1 | 2 |
| CLS-156 | 0.18 | 0.03 | Tabular | 1 | 2 |
| CLS-157 | 0.22 | 0.11 | Compact | 2 | 3 |
| CLS-158 | 0.14 | 0.09 | Compact | 2 | 2 |
| CLS-159 | 0.09 | 0.03 | Compact | 1 | 0 |
| CLS-160 | 0.32 | 0.06 | Elongate | 0 | 3 |
| CLS-161 | 0.12 | 0.02 | Tabular | 0 | 1 |
| CLS-162 | 0.12 | 0.08 | Compact | 3 | 0 |
| CLS-163 | 0.29 | 0.09 | Compact | 3 | 3 |
| CLS-164 | 0.14 | 0.01 | Tabular | 0 | 1 |
| CLS-165 | 0.08 | 0.02 | Tabular | 0 | 0 |
| CLS-166 | 0.17 | 0.11 | Compact | 1 | 2 |
| CLS-167 | 0.18 | 0.06 | Compact | 2 | 2 |
| CLS-168 | 0.21 | 0.09 | Compact | 3 | 2 |
| CLS-169 | 0.06 | 0.02 | Tabular | 1 | 0 |
| CLS-170 | 0.13 | 0.04 | Tabular | 1 | 2 |
| CLS-171 | 0.09 | 0.04 | Compact | 2 | 1 |
| CLS-172 | 0.13 | 0.05 | Compact | 0 | 2 |
| CLS-173 | 0.15 | 0.06 | Compact | 0 | 1 |
| CLS-174 | 0.12 | 0.04 | Tabular | 1 | 2 |
| CLS-175 | 0.13 | 0.04 | Elongate | 2 | 2 |
| CLS-176 | 0.06 | 0.03 | Compact | 1 | 0 |
| CLS-177 | 0.08 | 0.02 | Tabular | 1 | 0 |
| CLS-178 | 0.16 | 0.04 | Elongate | 0 | 2 |
| CLS-179 | 0.24 | 0.14 | Compact | 3 | 3 |
| CLS-180 | 0.17 | 0.06 | Compact | 2 | 3 |
| CLS-181 | 0.15 | 0.05 | Compact | 2 | 3 |
| CLS-182 | 0.07 | 0.05 | Compact | 3 | 2 |
| CLS-183 | 0.14 | 0.08 | Compact | 2 | 3 |
| CLS-184 | 0.07 | 0.03 | Compact | 1 | 2 |
| CLS-185 | 0.06 | 0.02 | Compact | 3 | 1 |
| CLS-186 | 0.08 | 0.03 | Compact | 1 | 1 |
| CLS-187 | 0.4 | 0.05 | Tabular | 2 | 1 |
| CLS-188 | 0.14 | 0.05 | Compact | 1 | 1 |
| CLS-189 | 0.07 | 0.02 | Tabular | 1 | 0 |
| CLS-190 | 0.08 | 0.01 | Tabular | 0 | 0 |
| CLS-191 | 0.11 | 0.07 | Compact | 3 | 2 |
| CLS-192 | 0.16 | 0.03 | Compact | 3 | 1 |
| CLS-193 | 0.11 | 0.02 | Tabular | 1 | 3 |
| CLS-194 | 0.09 | 0.06 | Compact | 3 | 3 |
| CLS-195 | 0.05 | 0.02 | Compact | 2 | 1 |
| CLS-196 | 0.21 | 0.03 | Tabular | 1 | 2 |
| CLS-197 | 0.2 | 0.06 | Elongate | 3 | 0 |
| CLS-198 | 0.21 | 0.08 | Compact | 2 | 2 |
| CLS-199 | 0.17 | 0.04 | Compact | 1 | 3 |
| CLS-200 | 0.08 | 0.03 | Compact | 1 | 2 |
| CLS-201 | 0.28 | 0.1 | Compact | 1 | 3 |
| CLS-202 | 0.08 | 0.05 | Compact | 3 | 1 |
| CLS-203 | 0.06 | 0.04 | Compact | 2 | 1 |
| CLS-204 | 0.12 | 0.03 | Compact | 2 | 2 |
| CLS-205 | 0.16 | 0.05 | Compact | 3 | 3 |
| CLS-206 | 0.15 | 0.14 | Compact | 3 | 3 |
| CLS-207 | 0.16 | 0.09 | Compact | 1 | 2 |
| CLS-208 | 0.2 | 0.08 | Compact | 1 | 0 |
| CLS-209 | 0.15 | 0.04 | Tabular | 0 | 1 |
| CLS-210 | 0.13 | 0.04 | Compact | 0 | 0 |
| CLS-211 | 0.17 | 0.06 | Compact | 1 | 1 |
| CLS-212 | 0.2 | 0.06 | Tabular | 2 | 3 |
| CLS-213 | 0.09 | 0.04 | Tabular | 1 | 0 |
| CLS-214 | 0.11 | 0.06 | Compact | 2 | 1 |
| CLS-215 | 0.08 | 0.03 | Tabular | 0 | 1 |
| CLS-216 | 0.23 | 0.09 | Elongate | 3 | 1 |
| CLS-217 | 0.17 | 0.05 | Tabular | 1 | 2 |
| CLS-218 | 0.13 | 0.04 | Compact | 2 | 2 |
| CLS-219 | 0.1 | 0.03 | Elongate | 2 | 1 |
| CLS-220 | 0.09 | 0.03 | Compact | 1 | 1 |
| CLS-221 | 0.32 | 0.03 | Tabular | 1 | 3 |
| CLS-222 | 0.08 | 0.05 | Compact | 0 | 0 |
| CLS-223 | 0.22 | 0.03 | Tabular | 0 | 3 |
| CLS-224 | 0.12 | 0.02 | Elongate | 1 | 1 |
| CLS-225 | 0.08 | 0.06 | Compact | 2 | 1 |
| CLS-226 | 0.06 | 0.03 | Compact | 2 | 3 |
| CLS-227 | 0.07 | 0.06 | Compact | 2 | 2 |
| CLS-228 | 0.13 | 0.04 | Tabular | 0 | 1 |
| CLS-229 | 0.08 | 0.05 | Compact | 3 | 1 |
| CLS-230 | 0.05 | 0.03 | Compact | 1 | 0 |
| CLS-231 | 0.18 | 0.08 | Compact | 1 | 1 |
| CLS-232 | 0.07 | 0.04 | Compact | 2 | 1 |
| CLS-233 | 0.07 | 0.04 | Compact | 1 | 1 |
| CLS-234 | 0.08 | 0.04 | Compact | 3 | 1 |
| CLS-235 | 0.2 | 0.05 | Tabular | 2 | 1 |
| CLS-236 | 0.13 | 0.03 | Tabular | 0 | 1 |
| CLS-237 | 0.18 | 0.08 | Compact | 3 | 2 |
| CLS-238 | 0.08 | 0.02 | Tabular | 0 | 1 |
| CLS-239 | 0.17 | 0.05 | Compact | 1 | 2 |
| CLS-240 | 0.08 | 0.03 | Tabular | 0 | 1 |
| CLS-241 | 0.26 | 0.09 | Compact | 2 | 2 |
| CLS-242 | 0.26 | 0.07 | Elongate | 3 | 3 |
| CLS-243 | 0.08 | 0.01 | Tabular | 1 | 3 |
| CLS-244 | 0.09 | 0.02 | Tabular | 3 | 1 |
| CLS-245 | 0.06 | 0.04 | Compact | 1 | 1 |
| CLS-246 | 0.17 | 0.07 | Compact | 0 | 3 |
| CLS-247 | 0.11 | 0.04 | Compact | 0 | 1 |
| CLS-248 | 0.1 | 0.02 | Tabular | 2 | 2 |
| CLS-249 | 0.11 | 0.09 | Compact | 1 | 2 |
| CLS-250 | 0.12 | 0.02 | Tabular | 1 | 1 |
| CLS-251 | 0.22 | 0.02 | Elongate | 3 | 3 |
| CLS-252 | 0.16 | 0.06 | Compact | 2 | 3 |
| CLS-253 | 0.13 | 0.07 | Compact | 3 | 2 |
| CLS-254 | 0.19 | 0.08 | Compact | 2 | 2 |
| CLS-255 | 0.09 | 0.02 | Tabular | 0 | 0 |
| CLS-256 | 0.1 | 0.03 | Compact | 3 | 3 |
| CLS-257 | 0.22 | 0.03 | Tabular | 2 | 2 |
| CLS-258 | 0.08 | 0.03 | Compact | 0 | 1 |
| CLS-259 | 0.1 | 0.01 | Tabular | 0 | 1 |
| CLS-260 | 0.17 | 0.02 | Tabular | 1 | 1 |
| CLS-261 | 0.13 | 0.04 | Compact | 1 | 1 |
| CLS-262 | 0.17 | 0.04 | Compact | 0 | 1 |
| CLS-263 | 0.15 | 0.11 | Compact | 2 | 2 |
| CLS-264 | 0.15 | 0.09 | Compact | 2 | 3 |
| CLS-265 | 0.14 | 0.01 | Tabular | 2 | 2 |
| CLS-266 | 0.14 | 0.02 | Tabular | 2 | 1 |
| CLS-267 | 0.13 | 0.02 | Tabular | 1 | 1 |
| CLS-268 | 0.09 | 0.01 | Tabular | 1 | 1 |
| CLS-269 | 0.1 | 0.02 | Tabular | 1 | 1 |
| CLS-270 | 0.11 | 0.02 | Tabular | 1 | 2 |
| CLS-271 | 0.09 | 0.01 | Tabular | 3 | 2 |
| CLS-272 | 0.17 | 0.11 | Compact | 1 | 3 |
| CLS-273 | 0.1 | 0.05 | Compact | 3 | 3 |
| CLS-274 | 0.05 | 0.02 | Tabular | 0 | 1 |
| CLS-275 | 0.22 | 0.14 | Compact | 2 | 3 |
| CLS-276 | 0.2 | 0.05 | Compact | 2 | 1 |
| CLS-277 | 0.1 | 0.01 | Tabular | 0 | 0 |
| CLS-278 | 0.13 | 0.04 | Compact | 0 | 1 |
| CLS-279 | 0.17 | 0.01 | Tabular | 3 | 3 |
| CLS-280 | 0.12 | 0.05 | Compact | 3 | 2 |
| CLS-281 | 0.1 | 0.05 | Compact | 1 | 2 |
| CLS-282 | 0.1 | 0.04 | Compact | 2 | 1 |
| CLS-283 | 0.17 | 0.03 | Compact | 3 | 2 |
| CLS-284 | 0.08 | 0.02 | Tabular | 0 | 1 |
| CLS-285 | 0.11 | 0.03 | Compact | 0 | 1 |
| CLS-286 | 0.14 | 0.05 | Compact | 2 | 1 |
| CLS-287 | 0.1 | 0.05 | Compact | 2 | 2 |
| CLS-288 | 0.13 | 0.04 | Tabular | 2 | 2 |
| CLS-289 | 0.12 | 0.04 | Compact | 3 | 2 |
| CLS-290 | 0.22 | 0.05 | Compact | 1 | 3 |
| CLS-291 | 0.08 | 0.01 | Tabular | 1 | 0 |
| CLS-292 | 0.14 | 0.03 | Compact | 2 | 2 |
| CLS-293 | 0.13 | 0.01 | Tabular | 2 | 1 |
| CLS-294 | 0.11 | 0.01 | Tabular | 1 | 3 |
| CLS-295 | 0.08 | 0.04 | Compact | 1 | 1 |
| CLS-296 | 0.13 | 0.02 | Tabular | 0 | 1 |
| CLS-297 | 0.1 | 0.04 | Compact | 2 | 2 |
| CLS-298 | 0.07 | 0.07 | Compact | 0 | 1 |
| CLS-299 | 0.15 | 0.05 | Compact | 3 | 1 |
| CLS-300 | 0.14 | 0.01 | Tabular | 0 | 1 |
| CLS-301 | 0.08 | 0.03 | Compact | 2 | 1 |
| CLS-302 | 0.16 | 0.05 | Compact | 3 | 2 |
| CLS-303 | 0.12 | 0.04 | Compact | 2 | 2 |
| CLS-304 | 0.13 | 0.02 | Tabular | 0 | 1 |
| CLS-305 | 0.15 | 0.08 | Compact | 1 | 2 |
| CLS-306 | 0.13 | 0.03 | Compact | 1 | 2 |
| CLS-307 | 0.18 | 0.08 | Compact | 0 | 3 |
| CLS-308 | 0.12 | 0.01 | Tabular | 0 | 1 |
| CLS-309 | 0.22 | 0.09 | Compact | 1 | 2 |
| CLS-310 | 0.18 | 0.07 | Compact | 2 | 2 |
| CLS-311 | 0.06 | 0.03 | Compact | 3 | 1 |
| CLS-312 | 0.05 | 0.04 | Compact | 2 | 3 |
| CLS-313 | 0.09 | 0.04 | Compact | 3 | 3 |
| CLS-314 | 0.09 | 0.02 | Tabular | 0 | 1 |
| CLS-315 | 0.25 | 0.08 | Compact | 3 | 3 |
| CLS-316 | 0.12 | 0.07 | Compact | 3 | 3 |
| CLS-317 | 0.12 | 0.05 | Compact | 3 | 2 |
| CLS-318 | 0.18 | 0.07 | Compact | 1 | 2 |
| CLS-319 | 0.09 | 0.06 | Compact | 0 | 2 |
| CLS-320 | 0.26 | 0.02 | Tabular | 0 | 0 |
| CLS-321 | 0.12 | 0.07 | Compact | 2 | 3 |
| CLS-322 | 0.08 | 0.05 | Compact | 3 | 3 |
| CLS-323 | 0.1 | 0.06 | Compact | 1 | 2 |
| CLS-324 | 0.11 | 0.04 | Compact | 2 | 1 |
| CLS-325 | 0.16 | 0.08 | Compact | 2 | 1 |
| CLS-326 | 0.15 | 0.07 | Elongate | 3 | 1 |
| CLS-327 | 0.13 | 0.09 | Compact | 3 | 3 |
| CLS-328 | 0.1 | 0.02 | Tabular | 3 | 1 |
| CLS-329 | 0.15 | 0.13 | Compact | 1 | 2 |
| CLS-330 | 0.09 | 0.02 | Compact | 2 | 3 |
| CLS-331 | 0.17 | 0.07 | Compact | 3 | 3 |
| CLS-332 | 0.09 | 0.04 | Compact | 2 | 1 |
| CLS-333 | 0.15 | 0.05 | Compact | 2 | 2 |
| CLS-334 | 0.19 | 0.08 | Compact | 2 | 2 |
| CLS-335 | 0.17 | 0.11 | Compact | 1 | 2 |
| CLS-336 | 0.14 | 0.07 | Compact | 3 | 3 |
| CLS-337 | 0.13 | 0.07 | Compact | 3 | 3 |
| CLS-338 | 0.11 | 0.06 | Compact | 1 | 3 |
| CLS-339 | 0.08 | 0.03 | Compact | 1 | 1 |
| CLS-340 | 0.07 | 0.03 | Elongate | 3 | 1 |
| CLS-341 | 0.14 | 0.05 | Compact | 1 | 3 |
| CLS-342 | 0.13 | 0.03 | Compact | 1 | 1 |
| CLS-343 | 0.11 | 0.01 | Tabular | 0 | 0 |
| CLS-344 | 0.1 | 0.05 | Compact | 3 | 3 |
| CLS-345 | 0.13 | 0.02 | Compact | 2 | 3 |
| CLS-346 | 0.12 | 0.05 | Compact | 3 | 3 |
| CLS-347 | 0.12 | 0.09 | Compact | 1 | 3 |
| CLS-348 | 0.11 | 0.08 | Compact | 1 | 2 |
| CLS-349 | 0.1 | 0.05 | Compact | 3 | 2 |
| CLS-350 | 0.14 | 0.08 | Compact | 3 | 2 |
| CLS-351 | 0.07 | 0.05 | Compact | 3 | 1 |
| CLS-352 | 0.15 | 0.06 | Compact | 1 | 3 |
| CLS-353 | 0.09 | 0.01 | Tabular | 0 | 0 |
| CLS-354 | 0.11 | 0.05 | Compact | 0 | 1 |
| CLS-355 | 0.09 | 0.05 | Elongate | 3 | 2 |
| CLS-356 | 0.14 | 0.04 | Compact | 0 | 0 |
| CLS-357 | 0.13 | 0.04 | Tabular | 1 | 2 |
| CLS-358 | 0.06 | 0.05 | Compact | 0 | 1 |
| CLS-359 | 0.08 | 0.03 | Compact | 0 | 1 |
| CLS-360 | 0.2 | 0.06 | Compact | 0 | 3 |
| CLS-361 | 0.1 | 0.03 | Compact | 3 | 2 |
| CLS-362 | 0.12 | 0.03 | Compact | 3 | 2 |
| CLS-363 | 0.12 | 0.02 | Tabular | 2 | 1 |
| CLS-364 | 0.11 | 0.02 | Tabular | 1 | 2 |
| CLS-365 | 0.1 | 0.06 | Compact | 3 | 3 |
| CLS-366 | 0.09 | 0.02 | Tabular | 2 | 1 |
| CLS-367 | 0.08 | 0.05 | Compact | 3 | 2 |
| CLS-368 | 0.09 | 0.03 | Compact | 1 | 1 |
| CLS-369 | 0.13 | 0.01 | Tabular | 2 | 0 |
| CLS-370 | 0.07 | 0.03 | Compact | 3 | 1 |
| CLS-371 | 0.07 | 0.04 | Compact | 3 | 2 |
| CLS-372 | 0.09 | 0.03 | Tabular | 1 | 0 |
| CLS-373 | 0.11 | 0.03 | Compact | 1 | 3 |
| CLS-374 | 0.06 | 0.02 | Compact | 2 | 1 |
| CLS-375 | 0.23 | 0.08 | Compact | 1 | 3 |
| CLS-376 | 0.16 | 0.03 | Tabular | 2 | 2 |
| CLS-377 | 0.09 | 0.02 | Tabular | 0 | 1 |
| CLS-378 | 0.04 | 0.03 | Compact | 0 | 1 |
| CLS-379 | 0.05 | 0.04 | Compact | 0 | 1 |
| CLS-380 | 0.05 | 0.03 | Compact | 2 | 2 |
| CLS-381 | 0.04 | 0.02 | Compact | 1 | 2 |
| CLS-382 | 0.08 | 0.06 | Compact | 0 | 2 |
| CLS-383 | 0.1 | 0.01 | Tabular | 0 | 2 |
| CLS-384 | 0.07 | 0.01 | Tabular | 0 | 1 |
| CLS-385 | 0.06 | 0.01 | Tabular | 3 | 1 |
| CLS-386 | 0.08 | 0.02 | Compact | 1 | 2 |
| CLS-387 | 0.08 | 0.02 | Tabular | 2 | 1 |
| CLS-388 | 0.05 | 0.03 | Compact | 2 | 2 |
| CLS-389 | 0.08 | 0.02 | Compact | 3 | 2 |
| CLS-390 | 0.1 | 0.02 | Tabular | 1 | 1 |
| CLS-391 | 0.11 | 0.02 | Tabular | 2 | 2 |
| CLS-392 | 0.1 | 0.05 | Tabular | 2 | 1 |
| CLS-393 | 0.13 | 0.03 | Tabular | 3 | 1 |
| CLS-394 | 0.09 | 0.02 | Tabular | 2 | 1 |
| CLS-395 | 0.12 | 0.02 | Compact | 1 | 1 |
| CLS-396 | 0.12 | 0.07 | Compact | 0 | 1 |
| CLS-397 | 0.13 | 0.03 | Compact | 2 | 3 |
| CLS-398 | 0.13 | 0.04 | Compact | 2 | 0 |
| CLS-399 | 0.06 | 0.05 | Compact | 3 | 0 |
| CLS-400 | 0.06 | 0.04 | Compact | 3 | 1 |
| CLS-401 | 0.12 | 0.02 | Tabular | 1 | 1 |
| CLS-402 | 0.16 | 0.03 | Tabular | 2 | 3 |
| CLS-403 | 0.11 | 0.05 | Tabular | 3 | 0 |
| CLS-404 | 0.18 | 0.02 | Tabular | 1 | 1 |
| CLS-405 | 0.14 | 0.03 | Tabular | 2 | 0 |
| CLS-406 | 0.08 | 0.02 | Compact | 3 | 3 |
| CLS-407 | 0.24 | 0.11 | Compact | 0 | 3 |
| CLS-408 | 0.08 | 0.02 | Tabular | 1 | 1 |
| CLS-409 | 0.09 | 0.04 | Compact | 0 | 1 |
| CLS-410 | 0.23 | 0.09 | Compact | 2 | 3 |
| CLS-411 | 0.09 | 0.04 | Compact | 0 | 1 |
| CLS-412 | 0.2 | 0.08 | Compact | 0 | 2 |
| CLS-413 | 0.16 | 0.01 | Tabular | 0 | 2 |
| CLS-414 | 0.19 | 0.04 | Compact | 2 | 2 |
| CLS-415 | 0.14 | 0.06 | Compact | 3 | 3 |
| CLS-416 | 0.14 | 0.05 | Compact | 1 | 2 |
| CLS-417 | 0.09 | 0.04 | Compact | 0 | 2 |
| CLS-418 | 0.12 | 0.03 | Elongate | 2 | 2 |
| CLS-419 | 0.15 | 0.03 | Compact | 2 | 1 |
| CLS-420 | 0.1 | 0.03 | Compact | 0 | 2 |
| CLS-421 | 0.1 | 0.01 | Tabular | 0 | 1 |
| CLS-422 | 0.1 | 0.03 | Compact | 2 | 2 |
| CLS-423 | 0.06 | 0.03 | Compact | 0 | 1 |
| CLS-424 | 0.07 | 0.01 | Tabular | 0 | 1 |
| CLS-425 | 0.13 | 0.02 | Tabular | 0 | 1 |
| CLS-426 | 0.09 | 0.05 | Compact | 3 | 3 |
| CLS-427 | 0.06 | 0.01 | Tabular | 0 | 2 |
| CLS-428 | 0.09 | 0.02 | Tabular | 1 | 0 |
| CLS-429 | 0.09 | 0.05 | Compact | 2 | 3 |
| CLS-430 | 0.25 | 0.1 | Compact | 1 | 2 |
| CLS-431 | 0.28 | 0.12 | Compact | 2 | 3 |
| CLS-432 | 0.11 | 0.03 | Compact | 0 | 1 |
| CLS-433 | 0.12 | 0.02 | Tabular | 3 | 1 |
| CLS-434 | 0.09 | 0.01 | Tabular | 3 | 3 |
| CLS-435 | 0.11 | 0.05 | Compact | 3 | 3 |
| CLS-436 | 0.11 | 0.09 | Compact | 0 | 3 |
| CLS-437 | 0.09 | 0.01 | Tabular | 0 | 0 |
| CLS-438 | 0.12 | 0.03 | Tabular | 2 | 1 |
| CLS-439 | 0.15 | 0.02 | Tabular | 1 | 1 |
| CLS-440 | 0.14 | 0.03 | Compact | 2 | 1 |
| CLS-441 | 0.07 | 0.02 | Compact | 2 | 2 |
| CLS-442 | 0.1 | 0.02 | Compact | 2 | 2 |
| CLS-443 | 0.07 | 0.02 | Compact | 2 | 1 |
| CLS-444 | 0.06 | 0.02 | Compact | 1 | 1 |
| CLS-445 | 0.12 | 0.02 | Compact | 2 | 3 |
| CLS-446 | 0.15 | 0.03 | Compact | 2 | 2 |
| CLS-447 | 0.07 | 0.02 | Tabular | 1 | 2 |
| CLS-448 | 0.07 | 0.03 | Compact | 1 | 1 |
| CLS-449 | 0.09 | 0.01 | Tabular | 0 | 1 |
| CLS-450 | 0.17 | 0.05 | Compact | 3 | 2 |
| CLS-451 | 0.1 | 0.02 | Tabular | 1 | 1 |
| CLS-452 | 0.08 | 0.02 | Tabular | 1 | 1 |
| CLS-453 | 0.12 | 0.03 | Compact | 1 | 2 |
| CLS-454 | 0.14 | 0.03 | Compact | 1 | 2 |
| CLS-455 | 0.1 | 0.02 | Compact | 3 | 2 |
| CLS-456 | 0.12 | 0.03 | Compact | 1 | 1 |
| CLS-457 | 0.1 | 0.02 | Tabular | 2 | 2 |
| CLS-458 | 0.18 | 0.05 | Compact | 1 | 3 |
| CLS-459 | 0.16 | 0.05 | Compact | 1 | 3 |
| CLS-460 | 0.09 | 0.01 | Tabular | 1 | 1 |
| CLS-461 | 0.12 | 0.02 | Tabular | 2 | 1 |
| CLS-462 | 0.07 | 0.01 | Tabular | 0 | 0 |
| CLS-463 | 0.06 | 0.05 | Compact | 0 | 2 |
| CLS-464 | 0.09 | 0.01 | Tabular | 0 | 1 |
| CLS-465 | 0.09 | 0.01 | Tabular | 1 | 0 |
| CLS-466 | 0.06 | 0.03 | Compact | 2 | 3 |
| CLS-467 | 0.06 | 0.04 | Compact | 1 | 3 |
| CLS-468 | 0.1 | 0.02 | Compact | 2 | 1 |
| CLS-469 | 0.1 | 0.01 | Tabular | 0 | 1 |
| CLS-470 | 0.05 | 0.02 | Compact | 2 | 1 |
| CLS-471 | 0.13 | 0.01 | Elongate | 1 | 1 |
| CLS-472 | 0.1 | 0.03 | Compact | 2 | 3 |
| CLS-473 | 0.09 | 0.05 | Compact | 0 | 1 |
| CLS-474 | 0.22 | 0.06 | Elongate | 1 | 3 |
| CLS-475 | 0.08 | 0.03 | Compact | 3 | 3 |
| CLS-476 | 0.12 | 0.06 | Compact | 3 | 3 |
| CLS-477 | 0.15 | 0.06 | Compact | 1 | 3 |
| CLS-478 | 0.11 | 0.03 | Tabular | 3 | 3 |
| CLS-479 | 0.06 | 0.04 | Compact | 1 | 1 |
| CLS-480 | 0.06 | 0.02 | Compact | 3 | 1 |
| CLS-481 | 0.07 | 0.04 | Compact | 1 | 1 |
| CLS-482 | 0.09 | 0.04 | Compact | 1 | 2 |
| CLS-483 | 0.12 | 0.02 | Tabular | 0 | 1 |
| CLS-484 | 0.13 | 0.02 | Tabular | 1 | 1 |
| CLS-485 | 0.09 | 0.02 | Tabular | 2 | 1 |
| CLS-486 | 0.1 | 0.02 | Tabular | 1 | 1 |
| CLS-487 | 0.06 | 0.01 | Tabular | 0 | 1 |
| CLS-488 | 0.11 | 0.02 | Tabular | 1 | 1 |
| CLS-489 | 0.13 | 0.03 | Compact | 0 | 2 |
| CLS-490 | 0.17 | 0.07 | Compact | 2 | 3 |
| CLS-491 | 0.16 | 0.01 | Elongate | 3 | 1 |
| CLS-492 | 0.14 | 0.08 | Compact | 2 | 3 |
| CLS-493 | 0.09 | 0.04 | Tabular | 2 | 1 |
| CLS-494 | 0.15 | 0.06 | Compact | 0 | 1 |
| CLS-495 | 0.09 | 0.02 | Tabular | 3 | 0 |
| CLS-496 | 0.07 | 0.03 | Tabular | 0 | 1 |
| CLS-497 | 0.2 | 0.08 | Compact | 0 | 3 |
| CLS-498 | 0.11 | 0.07 | Compact | 1 | 1 |
| CLS-499 | 0.1 | 0.03 | Compact | 0 | 0 |
| CLS-500 | 0.12 | 0.05 | Compact | 1 | 1 |
| CLS-501 | 0.09 | 0.01 | Tabular | 0 | 0 |
| CLS-502 | 0.12 | 0.04 | Tabular | 2 | 2 |
| CLS-503 | 0.13 | 0.04 | Compact | 0 | 1 |
| CLS-504 | 0.11 | 0.06 | Compact | 0 | 2 |
| CLS-505 | 0.19 | 0.1 | Compact | 2 | 2 |
| CLS-506 | 0.1 | 0.04 | Compact | 0 | 1 |
| CLS-507 | 0.09 | 0.02 | Tabular | 3 | 2 |
| CLS-508 | 0.06 | 0.01 | Tabular | 1 | 1 |
| CLS-509 | 0.06 | 0.03 | Compact | 3 | 1 |
| CLS-510 | 0.12 | 0.03 | Tabular | 2 | 2 |
| CLS-511 | 0.17 | 0.05 | Compact | 3 | 2 |
| CLS-512 | 0.07 | 0.04 | Compact | 1 | 1 |
| CLS-513 | 0.09 | 0.04 | Compact | 3 | 3 |
| CLS-514 | 0.06 | 0.06 | Compact | 2 | 1 |
| CLS-515 | 0.08 | 0.02 | Compact | 3 | 3 |
| CLS-516 | 0.12 | 0.05 | Compact | 2 | 2 |
| CLS-517 | 0.11 | 0.03 | Compact | 3 | 1 |
| CLS-518 | 0.11 | 0.06 | Compact | 3 | 3 |
| CLS-519 | 0.1 | 0.01 | Tabular | 2 | 1 |
| CLS-520 | 0.11 | 0.02 | Tabular | 1 | 1 |
| CLS-521 | 0.11 | 0.03 | Compact | 2 | 1 |
| CLS-522 | 0.09 | 0.02 | Tabular | 2 | 2 |
| CLS-523 | 0.1 | 0.02 | Tabular | 1 | 1 |
| CLS-524 | 0.08 | 0.06 | Compact | 2 | 3 |
| CLS-525 | 0.09 | 0.03 | Compact | 2 | 3 |
| CLS-526 | 0.07 | 0.02 | Tabular | 1 | 0 |
| CLS-527 | 0.14 | 0.08 | Compact | 2 | 2 |
| CLS-528 | 0.13 | 0.05 | Compact | 2 | 2 |
| CLS-529 | 0.06 | 0.03 | Compact | 3 | 1 |
| CLS-530 | 0.08 | 0.05 | Compact | 2 | 2 |
| CLS-531 | 0.11 | 0.04 | Compact | 3 | 2 |
| CLS-532 | 0.13 | 0.02 | Tabular | 3 | 2 |
| CLS-533 | 0.06 | 0.04 | Compact | 3 | 3 |
| CLS-534 | 0.06 | 0.02 | Compact | 1 | 1 |
| CLS-535 | 0.06 | 0.02 | Tabular | 3 | 1 |
| CLS-536 | 0.09 | 0.02 | Tabular | 1 | 2 |
| CLS-537 | 0.07 | 0.02 | Tabular | 3 | 2 |
| CLS-538 | 0.06 | 0.01 | Tabular | 2 | 1 |
| CLS-539 | 0.06 | 0.01 | Tabular | 1 | 2 |
| CLS-540 | 0.08 | 0.03 | Tabular | 3 | 3 |
| CLS-541 | 0.1 | 0.05 | Compact | 2 | 1 |
| CLS-542 | 0.09 | 0.01 | Tabular | 1 | 1 |
| CLS-543 | 0.09 | 0.05 | Compact | 0 | 3 |
| CLS-544 | 0.06 | 0.01 | Tabular | 2 | 2 |
| CLS-545 | 0.06 | 0.01 | Tabular | 3 | 3 |
| CLS-546 | 0.06 | 0.04 | Compact | 0 | 1 |
| CLS-547 | 0.06 | 0.02 | Tabular | 0 | 1 |
| CLS-548 | 0.09 | 0.02 | Tabular | 3 | 3 |
| CLS-549 | 0.06 | 0.02 | Compact | 2 | 2 |
| CLS-550 | 0.07 | 0.02 | Tabular | 2 | 1 |
| CLS-551 | 0.05 | 0.03 | Compact | 3 | 3 |
| CLS-552 | 0.06 | 0.04 | Compact | 3 | 1 |
| CLS-553 | 0.1 | 0.02 | Tabular | 0 | 1 |
| CLS-554 | 0.15 | 0.05 | Compact | 2 | 1 |
| CLS-555 | 0.09 | 0.02 | Tabular | 3 | 2 |
| CLS-556 | 0.11 | 0.01 | Tabular | 2 | 1 |
| CLS-557 | 0.12 | 0.03 | Compact | 1 | 1 |
| CLS-558 | 0.15 | 0.05 | Compact | 2 | 1 |
| CLS-559 | 0.11 | 0.03 | Tabular | 2 | 2 |
| CLS-560 | 0.09 | 0.02 | Tabular | 3 | 1 |
| CLS-561 | 0.09 | 0.04 | Compact | 3 | 3 |
| CLS-562 | 0.08 | 0.01 | Tabular | 1 | 2 |
| CLS-563 | 0.08 | 0.02 | Tabular | 1 | 3 |
| CLS-564 | 0.06 | 0.04 | Compact | 3 | 1 |
| CLS-565 | 0.06 | 0.01 | Tabular | 3 | 2 |
| CLS-566 | 0.18 | 0.06 | Compact | 1 | 3 |
| CLS-567 | 0.08 | 0.06 | Compact | 2 | 2 |
| CLS-568 | 0.07 | 0.05 | Compact | 2 | 2 |
| CLS-569 | 0.15 | 0.02 | Elongate | 3 | 1 |
| CLS-570 | 0.1 | 0.03 | Compact | 2 | 1 |
| CLS-571 | 0.07 | 0.01 | Tabular | 1 | 2 |
| CLS-572 | 0.13 | 0.02 | Tabular | 2 | 2 |
| CLS-573 | 0.1 | 0.05 | Compact | 3 | 1 |
| CLS-574 | 0.04 | 0.01 | Tabular | 0 | 0 |
| CLS-575 | 0.08 | 0.02 | Compact | 2 | 1 |
| CLS-576 | 0.06 | 0.02 | Tabular | 3 | 1 |
| CLS-577 | 0.09 | 0.02 | Compact | 2 | 3 |
| CLS-578 | 0.06 | 0.02 | Tabular | 1 | 1 |
| CLS-579 | 0.09 | 0.04 | Compact | 3 | 2 |
| CLS-580 | 0.07 | 0.03 | Compact | 0 | 1 |
| CLS-581 | 0.09 | 0.03 | Compact | 1 | 1 |
| CLS-582 | 0.07 | 0.03 | Compact | 1 | 1 |
| CLS-583 | 0.07 | 0.03 | Compact | 3 | 2 |
| CLS-584 | 0.1 | 0.02 | Compact | 3 | 2 |
| CLS-585 | 0.06 | 0.02 | Compact | 3 | 3 |
| CLS-586 | 0.08 | 0.04 | Compact | 3 | 3 |
| CLS-587 | 0.13 | 0.04 | Compact | 3 | 2 |
| CLS-588 | 0.08 | 0.01 | Tabular | 3 | 1 |
| CLS-589 | 0.05 | 0.03 | Compact | 2 | 2 |
| CLS-590 | 0.1 | 0.02 | Tabular | 3 | 2 |
| CLS-591 | 0.08 | 0.02 | Tabular | 1 | 1 |
| CLS-592 | 0.1 | 0.02 | Compact | 2 | 1 |
| CLS-593 | 0.08 | 0.04 | Compact | 3 | 2 |
| CLS-594 | 0.07 | 0.03 | Compact | 3 | 2 |
| CLS-595 | 0.07 | 0.03 | Compact | 1 | 1 |
| CLS-596 | 0.08 | 0.03 | Compact | 3 | 3 |
| CLS-597 | 0.06 | 0.05 | Compact | 2 | 2 |
| CLS-598 | 0.09 | 0.03 | Compact | 3 | 3 |
| CLS-599 | 0.12 | 0.03 | Compact | 2 | 2 |
| CLS-600 | 0.11 | 0.01 | Tabular | 2 | 1 |
| CLS-601 | 0.07 | 0.04 | Compact | 3 | 1 |
| CLS-602 | 0.06 | 0.03 | Compact | 3 | 1 |
| CLS-603 | 0.06 | 0.02 | Compact | 3 | 1 |
| CLS-604 | 0.07 | 0.02 | Tabular | 1 | 1 |
| CLS-605 | 0.12 | 0.03 | Compact | 3 | 2 |
| CLS-606 | 0.09 | 0.01 | Tabular | 0 | 1 |
| CLS-607 | 0.1 | 0.02 | Compact | 2 | 1 |
| CLS-608 | 0.07 | 0.03 | Compact | 1 | 1 |
| CLS-609 | 0.08 | 0.05 | Compact | 3 | 2 |
| CLS-610 | 0.05 | 0.04 | Compact | 0 | 2 |
| CLS-611 | 0.1 | 0.03 | Compact | 2 | 3 |
| CLS-612 | 0.07 | 0.04 | Compact | 3 | 2 |
| CLS-613 | 0.09 | 0.05 | Compact | 3 | 3 |
| CLS-614 | 0.09 | 0.04 | Compact | 2 | 2 |
| CLS-615 | 0.08 | 0.02 | Tabular | 1 | 1 |
| CLS-616 | 0.1 | 0.04 | Compact | 1 | 1 |
| CLS-617 | 0.09 | 0.04 | Compact | 2 | 3 |
| CLS-618 | 0.14 | 0.04 | Compact | 0 | 2 |
| CLS-619 | 0.05 | 0.01 | Tabular | 2 | 2 |
| CLS-620 | 0.05 | 0.04 | Compact | 3 | 1 |
| CLS-621 | 0.07 | 0.01 | Tabular | 2 | 1 |
| CLS-622 | 0.07 | 0.03 | Compact | 1 | 2 |
| CLS-623 | 0.06 | 0.02 | Compact | 3 | 2 |
| CLS-624 | 0.15 | 0.01 | Elongate | 1 | 2 |
| CLS-625 | 0.08 | 0.02 | Tabular | 0 | 1 |
| CLS-626 | 0.06 | 0.01 | Compact | 2 | 2 |
| CLS-627 | 0.1 | 0.02 | Compact | 2 | 2 |
| CLS-628 | 0.1 | 0.01 | Tabular | 3 | 1 |
| CLS-629 | 0.06 | 0.01 | Tabular | 1 | 1 |
| CLS-630 | 0.09 | 0.05 | Compact | 3 | 3 |
| CLS-631 | 0.09 | 0.02 | Tabular | 2 | 1 |
| CLS-632 | 0.09 | 0.01 | Elongate | 1 | 1 |
| CLS-633 | 0.1 | 0.02 | Elongate | 1 | 1 |
| CLS-634 | 0.08 | 0.02 | Compact | 3 | 2 |
| CLS-635 | 0.1 | 0.02 | Compact | 2 | 1 |
| CLS-636 | 0.07 | 0.01 | Tabular | 1 | 1 |
| CLS-637 | 0.06 | 0.02 | Compact | 3 | 1 |
| CLS-638 | 0.06 | 0.02 | Compact | 1 | 1 |
| CLS-639 | 0.07 | 0.05 | Compact | 2 | 1 |
| CLS-640 | 0.07 | 0.02 | Compact | 1 | 3 |
| CLS-641 | 0.12 | 0.02 | Elongate | 1 | 2 |
| CLS-642 | 0.07 | 0.02 | Tabular | 1 | 1 |
| CLS-643 | 0.09 | 0.03 | Compact | 1 | 1 |
| CLS-644 | 0.09 | 0.02 | Compact | 2 | 2 |
| CLS-645 | 0.06 | 0.01 | Tabular | 1 | 1 |
| CLS-646 | 0.07 | 0.03 | Compact | 3 | 1 |
| CLS-647 | 0.09 | 0.05 | Compact | 2 | 1 |
| CLS-648 | 0.11 | 0.02 | Tabular | 1 | 1 |
| CLS-649 | 0.1 | 0.03 | Compact | 0 | 1 |
| CLS-650 | 0.06 | 0.04 | Compact | 3 | 2 |
| CLS-651 | 0.06 | 0.03 | Compact | 1 | 1 |
| CLS-652 | 0.06 | 0.04 | Compact | 2 | 2 |
| CLS-653 | 0.05 | 0.04 | Compact | 3 | 2 |
| CLS-654 | 0.07 | 0.02 | Compact | 2 | 1 |
| CLS-655 | 0.06 | 0.02 | Tabular | 2 | 1 |
| CLS-656 | 0.1 | 0.02 | Tabular | 0 | 1 |
| CLS-657 | 0.09 | 0.05 | Compact | 3 | 1 |
| CLS-658 | 0.06 | 0.05 | Compact | 1 | 3 |
| CLS-659 | 0.09 | 0.05 | Compact | 3 | 3 |
| CLS-660 | 0.06 | 0.03 | Compact | 1 | 1 |
| CLS-661 | 0.05 | 0.03 | Compact | 3 | 3 |
| CLS-662 | 0.07 | 0.04 | Compact | 1 | 3 |
| CLS-663 | 0.1 | 0.05 | Compact | 1 | 3 |
| CLS-664 | 0.1 | 0.03 | Compact | 2 | 1 |
| CLS-665 | 0.06 | 0.04 | Compact | 3 | 1 |
| CLS-666 | 0.09 | 0.02 | Compact | 1 | 2 |
| CLS-667 | 0.08 | 0.02 | Tabular | 2 | 2 |
| CLS-668 | 0.06 | 0.02 | Compact | 3 | 2 |
| CLS-669 | 0.06 | 0.02 | Compact | 2 | 2 |
| CLS-670 | 0.05 | 0.04 | Compact | 3 | 3 |
| CLS-671 | 0.05 | 0.02 | Tabular | 1 | 2 |
| CLS-672 | 0.07 | 0.04 | Compact | 3 | 3 |
| CLS-673 | 0.05 | 0.02 | Compact | 2 | 1 |
| CLS-674 | 0.07 | 0.02 | Compact | 1 | 3 |
| CLS-675 | 0.07 | 0.02 | Tabular | 1 | 2 |
| CLS-676 | 0.05 | 0.04 | Compact | 3 | 3 |
| CLS-677 | 0.06 | 0.04 | Compact | 3 | 2 |
| CLS-678 | 0.06 | 0.03 | Compact | 3 | 3 |
| CLS-679 | 0.1 | 0.04 | Compact | 1 | 1 |
| CLS-680 | 0.1 | 0.01 | Tabular | 1 | 2 |
| CLS-681 | 0.08 | 0.02 | Tabular | 1 | 1 |
| CLS-682 | 0.08 | 0.02 | Compact | 3 | 3 |
| CLS-683 | 0.09 | 0.05 | Compact | 1 | 2 |
| CLS-684 | 0.07 | 0.01 | Tabular | 3 | 3 |
| CLS-685 | 0.04 | 0.02 | Compact | 1 | 2 |
| CLS-686 | 0.07 | 0.05 | Compact | 2 | 3 |
| CLS-687 | 0.07 | 0.02 | Compact | 3 | 3 |
| CLS-688 | 0.06 | 0.02 | Tabular | 1 | 1 |
| CLS-689 | 0.07 | 0.06 | Compact | 1 | 1 |
| CLS-690 | 0.06 | 0.03 | Compact | 3 | 3 |
| CLS-691 | 0.07 | 0.05 | Compact | 3 | 1 |
| CLS-692 | 0.06 | 0.02 | Compact | 1 | 2 |
| CLS-693 | 0.06 | 0.02 | Compact | 3 | 1 |
| CLS-694 | 0.06 | 0.01 | Tabular | 1 | 1 |
| CLS-695 | 0.06 | 0.01 | Tabular | 2 | 1 |
| CLS-696 | 0.06 | 0.04 | Compact | 3 | 3 |
| CLS-697 | 0.06 | 0.04 | Compact | 3 | 3 |
| CLS-698 | 0.09 | 0.05 | Compact | 0 | 2 |
| CLS-699 | 0.11 | 0.04 | Compact | 2 | 1 |
| CLS-700 | 0.07 | 0.03 | Tabular | 1 | 2 |
| CLS-701 | 0.1 | 0.02 | Tabular | 2 | 1 |
| CLS-702 | 0.06 | 0.03 | Compact | 1 | 1 |
| CLS-703 | 0.07 | 0.03 | Compact | 0 | 1 |
| CLS-704 | 0.13 | 0.06 | Compact | 3 | 2 |
| CLS-705 | 0.09 | 0.02 | Tabular | 0 | 1 |
| CLS-706 | 0.12 | 0.06 | Compact | 1 | 1 |
| CLS-707 | 0.09 | 0.03 | Compact | 3 | 2 |
| CLS-708 | 0.1 | 0.03 | Tabular | 2 | 2 |
| CLS-709 | 0.09 | 0.03 | Compact | 1 | 1 |
| CLS-710 | 0.11 | 0.04 | Compact | 2 | 3 |
| CLS-711 | 0.1 | 0.02 | Tabular | 0 | 1 |
| CLS-712 | 0.09 | 0.05 | Compact | 3 | 1 |
| CLS-713 | 0.09 | 0.01 | Tabular | 3 | 2 |
| CLS-714 | 0.08 | 0.02 | Tabular | 1 | 1 |
| CLS-715 | 0.1 | 0.02 | Compact | 3 | 2 |
| CLS-716 | 0.09 | 0.03 | Compact | 0 | 1 |
| CLS-717 | 0.09 | 0.03 | Compact | 1 | 2 |
| CLS-718 | 0.16 | 0.02 | Tabular | 0 | 3 |
| CLS-719 | 0.14 | 0.02 | Tabular | 2 | 1 |
| CLS-720 | 0.07 | 0.05 | Compact | 1 | 2 |
| CLS-721 | 0.15 | 0.05 | Tabular | 1 | 2 |
| CLS-722 | 0.13 | 0.03 | Tabular | 3 | 3 |
| CLS-723 | 0.13 | 0.06 | Compact | 2 | 3 |
| CLS-724 | 0.1 | 0.02 | Compact | 2 | 2 |
| CLS-725 | 0.08 | 0.02 | Tabular | 3 | 2 |
| CLS-726 | 0.09 | 0.02 | Tabular | 2 | 1 |
| CLS-727 | 0.1 | 0.02 | Tabular | 3 | 2 |
| CLS-728 | 0.09 | 0.05 | Compact | 1 | 1 |
| CLS-729 | 0.08 | 0.04 | Compact | 1 | 1 |
| CLS-730 | 0.22 | 0.08 | Compact | 3 | 3 |
| CLS-731 | 0.14 | 0.01 | Tabular | 2 | 3 |
| CLS-732 | 0.07 | 0.01 | Tabular | 0 | 3 |
| CLS-733 | 0.13 | 0.03 | Compact | 1 | 1 |
| CLS-734 | 0.28 | 0.09 | Compact | 3 | 2 |
| CLS-735 | 0.11 | 0.04 | Compact | 3 | 2 |
| CLS-736 | 0.07 | 0.05 | Compact | 3 | 2 |
| CLS-737 | 0.08 | 0.05 | Compact | 1 | 2 |
| CLS-738 | 0.08 | 0.01 | Tabular | 1 | 1 |
| CLS-739 | 0.08 | 0.02 | Tabular | 3 | 2 |
| CLS-740 | 0.12 | 0.05 | Compact | 1 | 3 |
| CLS-741 | 0.16 | 0.03 | Compact | 0 | 3 |
| CLS-742 | 0.16 | 0.03 | Tabular | 1 | 3 |
| CLS-743 | 0.06 | 0.01 | Tabular | 2 | 1 |
| CLS-744 | 0.1 | 0.02 | Tabular | 2 | 2 |
| CLS-745 | 0.08 | 0.04 | Compact | 3 | 2 |
| CLS-746 | 0.11 | 0.02 | Tabular | 1 | 1 |
| CLS-747 | 0.07 | 0.03 | Tabular | 3 | 3 |
| CLS-748 | 0.07 | 0.02 | Tabular | 2 | 1 |
| CLS-749 | 0.08 | 0.03 | Compact | 1 | 3 |
| CLS-750 | 0.1 | 0.05 | Compact | 3 | 3 |
| CLS-751 | 0.06 | 0.02 | Compact | 1 | 1 |
| CLS-752 | 0.17 | 0.03 | Tabular | 2 | 3 |
| CLS-753 | 0.1 | 0.03 | Compact | 1 | 3 |
| CLS-754 | 0.08 | 0.07 | Compact | 2 | 3 |
| CLS-755 | 0.07 | 0.04 | Compact | 1 | 1 |
| CLS-756 | 0.08 | 0.03 | Tabular | 1 | 2 |
| CLS-757 | 0.13 | 0.04 | Compact | 1 | 2 |
| CLS-758 | 0.08 | 0.03 | Compact | 3 | 2 |
| CLS-759 | 0.07 | 0.03 | Compact | 3 | 3 |
| CLS-760 | 0.11 | 0.05 | Compact | 2 | 2 |
| CLS-761 | 0.09 | 0.05 | Compact | 2 | 1 |
| CLS-762 | 0.11 | 0.02 | Compact | 1 | 2 |
| CLS-763 | 0.15 | 0.03 | Elongate | 0 | 3 |
| CLS-764 | 0.08 | 0.02 | Compact | 0 | 3 |
| CLS-765 | 0.07 | 0.02 | Tabular | 1 | 1 |
| CLS-766 | 0.09 | 0.03 | Compact | 0 | 1 |
| CLS-767 | 0.1 | 0.02 | Tabular | 3 | 2 |
| CLS-768 | 0.11 | 0.05 | Compact | 3 | 1 |
| CLS-769 | 0.14 | 0.06 | Compact | 3 | 3 |
| CLS-770 | 0.15 | 0.07 | Compact | 3 | 3 |
| CLS-771 | 0.09 | 0.02 | Compact | 0 | 0 |
| CLS-772 | 0.12 | 0.02 | Tabular | 1 | 2 |
| CLS-773 | 0.1 | 0.03 | Compact | 2 | 3 |
| CLS-774 | 0.21 | 0.02 | Tabular | 2 | 3 |
| CLS-775 | 0.06 | 0.03 | Compact | 0 | 3 |
| CLS-776 | 0.06 | 0.02 | Compact | 2 | 1 |
| CLS-777 | 0.08 | 0.03 | Compact | 1 | 1 |
| CLS-778 | 0.11 | 0.04 | Compact | 1 | 3 |
| CLS-779 | 0.1 | 0.02 | Compact | 1 | 3 |
| CLS-780 | 0.11 | 0.02 | Tabular | 1 | 1 |
| CLS-781 | 0.12 | 0.02 | Tabular | 1 | 2 |
| CLS-782 | 0.2 | 0.1 | Compact | 3 | 3 |
| CLS-783 | 0.05 | 0.03 | Compact | 1 | 1 |
| CLS-784 | 0.12 | 0.02 | Compact | 1 | 2 |
| CLS-785 | 0.11 | 0.04 | Compact | 3 | 3 |
| CLS-786 | 0.25 | 0.11 | Compact | 1 | 3 |
| CLS-787 | 0.11 | 0.02 | Compact | 2 | 1 |
| CLS-788 | 0.07 | 0.02 | Tabular | 3 | 2 |
| CLS-789 | 0.16 | 0.03 | Elongate | 0 | 3 |
| CLS-790 | 0.06 | 0.02 | Tabular | 2 | 2 |
| CLS-791 | 0.13 | 0.02 | Tabular | 1 | 1 |
| CLS-792 | 0.05 | 0.01 | Tabular | 1 | 2 |
| CLS-793 | 0.08 | 0.03 | Compact | 2 | 2 |
| CLS-794 | 0.06 | 0.05 | Compact | 1 | 1 |
| CLS-795 | 0.09 | 0.02 | Compact | 1 | 2 |
| CLS-796 | 0.13 | 0.02 | Tabular | 2 | 1 |
| CLS-797 | 0.05 | 0.01 | Tabular | 0 | 1 |
| CLS-798 | 0.08 | 0.04 | Compact | 3 | 3 |
| CLS-799 | 0.11 | 0.06 | Compact | 3 | 3 |
| CLS-800 | 0.08 | 0.02 | Compact | 2 | 2 |
| CLS-801 | 0.05 | 0.04 | Compact | 2 | 3 |
| CLS-802 | 0.07 | 0.02 | Compact | 1 | 3 |
| CLS-803 | 0.1 | 0.02 | Tabular | 1 | 1 |
| CLS-804 | 0.07 | 0.01 | Tabular | 2 | 1 |
| CLS-805 | 0.06 | 0.03 | Compact | 3 | 3 |
| CLS-806 | 0.08 | 0.03 | Compact | 2 | 2 |
| CLS-807 | 0.06 | 0.03 | Compact | 1 | 2 |
| CLS-808 | 0.07 | 0.03 | Compact | 1 | 2 |
| CLS-809 | 0.11 | 0.03 | Compact | 2 | 2 |
| CLS-810 | 0.09 | 0.02 | Tabular | 1 | 2 |
| CLS-811 | 0.06 | 0.02 | Compact | 2 | 3 |
| CLS-812 | 0.07 | 0.03 | Tabular | 1 | 0 |
| CLS-813 | 0.06 | 0.03 | Compact | 1 | 1 |
| CLS-814 | 0.06 | 0.02 | Tabular | 3 | 1 |
| CLS-815 | 0.07 | 0.04 | Compact | 2 | 2 |
| CLS-816 | 0.09 | 0.03 | Compact | 3 | 3 |
| CLS-817 | 0.08 | 0.02 | Tabular | 1 | 1 |
| CLS-818 | 0.1 | 0.02 | Compact | 2 | 1 |
| CLS-819 | 0.07 | 0.02 | Compact | 3 | 2 |
| CLS-820 | 0.08 | 0.02 | Compact | 2 | 1 |
| CLS-821 | 0.06 | 0.03 | Compact | 1 | 2 |
| CLS-822 | 0.08 | 0.01 | Tabular | 3 | 2 |
| CLS-823 | 0.08 | 0.03 | Compact | 1 | 2 |
| CLS-824 | 0.1 | 0.02 | Compact | 1 | 2 |
| CLS-825 | 0.11 | 0.02 | Tabular | 1 | 1 |
| CLS-826 | 0.13 | 0.05 | Compact | 2 | 3 |
| CLS-827 | 0.05 | 0.01 | Tabular | 3 | 1 |
| CLS-828 | 0.06 | 0.02 | Compact | 0 | 2 |
| CLS-829 | 0.07 | 0.02 | Compact | 1 | 2 |
| CLS-830 | 0.06 | 0.02 | Compact | 1 | 3 |
| CLS-831 | 0.08 | 0.02 | Tabular | 2 | 2 |
| CLS-832 | 0.06 | 0.02 | Compact | 1 | 1 |
| CLS-833 | 0.1 | 0.05 | Compact | 2 | 3 |
| CLS-834 | 0.13 | 0.03 | Tabular | 2 | 3 |
| CLS-835 | 0.08 | 0.02 | Compact | 3 | 1 |
| CLS-836 | 0.07 | 0.01 | Tabular | 1 | 2 |
| CLS-837 | 0.16 | 0.02 | Tabular | 2 | 1 |
| CLS-838 | 0.13 | 0.03 | Compact | 3 | 3 |
| CLS-839 | 0.09 | 0.03 | Compact | 1 | 2 |
| CLS-840 | 0.11 | 0.06 | Compact | 2 | 1 |
| CLS-841 | 0.06 | 0.02 | Compact | 3 | 3 |
| CLS-842 | 0.08 | 0.02 | Compact | 1 | 2 |
| CLS-843 | 0.06 | 0.05 | Compact | 1 | 3 |
| CLS-844 | 0.06 | 0.03 | Compact | 1 | 2 |
| CLS-845 | 0.08 | 0.05 | Compact | 2 | 3 |
| CLS-846 | 0.07 | 0.03 | Compact | 2 | 1 |
| CLS-847 | 0.12 | 0.04 | Elongate | 2 | 3 |
| CLS-848 | 0.09 | 0.03 | Compact | 2 | 2 |
| CLS-849 | 0.06 | 0.01 | Tabular | 1 | 1 |
| CLS-850 | 0.06 | 0.02 | Tabular | 1 | 2 |
| CLS-851 | 0.08 | 0.04 | Compact | 2 | 3 |
| CLS-852 | 0.07 | 0.06 | Compact | 1 | 2 |
| CLS-853 | 0.09 | 0.02 | Tabular | 2 | 3 |
| CLS-854 | 0.06 | 0.02 | Compact | 1 | 1 |
| CLS-855 | 0.06 | 0.02 | Compact | 3 | 1 |
| CLS-856 | 0.06 | 0.02 | Compact | 3 | 3 |
| CLS-857 | 0.06 | 0.03 | Compact | 3 | 3 |
| CLS-858 | 0.08 | 0.03 | Compact | 1 | 2 |
| CLS-859 | 0.06 | 0.05 | Compact | 1 | 3 |
| CLS-860 | 0.1 | 0.05 | Compact | 3 | 3 |
| CLS-861 | 0.07 | 0.03 | Tabular | 1 | 2 |
| CLS-862 | 0.08 | 0.04 | Compact | 1 | 2 |
| CLS-863 | 0.05 | 0.03 | Compact | 2 | 1 |
| CLS-864 | 0.06 | 0.04 | Compact | 2 | 1 |
| CLS-865 | 0.06 | 0.02 | Compact | 1 | 3 |
| CLS-866 | 0.07 | 0.02 | Compact | 3 | 2 |
| CLS-867 | 0.07 | 0.03 | Compact | 1 | 1 |
| CLS-868 | 0.11 | 0.05 | Compact | 1 | 3 |
| CLS-869 | 0.05 | 0.01 | Compact | 3 | 2 |
| CLS-870 | 0.07 | 0.06 | Compact | 3 | 3 |
| CLS-871 | 0.06 | 0.03 | Compact | 2 | 1 |
| CLS-872 | 0.1 | 0.04 | Compact | 2 | 3 |
| CLS-873 | 0.06 | 0.01 | Tabular | 1 | 2 |
| CLS-874 | 0.07 | 0.03 | Compact | 2 | 2 |
| CLS-875 | 0.06 | 0.02 | Compact | 2 | 2 |
| CLS-876 | 0.07 | 0.03 | Compact | 1 | 3 |
| CLS-877 | 0.08 | 0.02 | Tabular | 2 | 3 |
| CLS-878 | 0.06 | 0.04 | Compact | 2 | 1 |
| CLS-879 | 0.07 | 0.02 | Compact | 3 | 2 |
| CLS-880 | 0.09 | 0.03 | Compact | 3 | 3 |
| CLS-881 | 0.44 | 0.11 | Tabular | 2 | 2 |
| CLS-882 | 0.24 | 0.12 | Compact | 1 | 1 |
| CLS-883 | 0.45 | 0.07 | Tabular | 1 | 2 |
| CLS-884 | 0.22 | 0.11 | Compact | 1 | 2 |
| CLS-885 | 0.41 | 0.12 | Compact | 2 | 3 |
| CLS-886 | 0.39 | 0.11 | Compact | 2 | 3 |
| CLS-887 | 0.35 | 0.17 | Compact | 3 | 2 |
| CLS-888 | 0.38 | 0.21 | Compact | 1 | 2 |
| CLS-889 | 0.48 | 0.16 | Compact | 1 | 2 |
| CLS-890 | 0.32 | 0.13 | Compact | 3 | 3 |
| CLS-891 | 0.37 | 0.19 | Compact | 2 | 2 |
| CLS-892 | 0.37 | 0.2 | Compact | 2 | 2 |
| CLS-893 | 0.39 | 0.18 | Compact | 2 | 3 |
| CLS-894 | 0.41 | 0.27 | Elongate | 2 | 1 |
| CLS-895 | 0.63 | 0.31 | Elongate | 3 | 1 |
| CLS-896 | 0.37 | 0.26 | Compact | 2 | 2 |
| CLS-897 | 1.04 | 0.26 | Tabular | 3 | 3 |
| CLS-898 | 0.63 | 0.19 | Tabular | 3 | 3 |
| CLS-899 | 0.54 | 0.21 | Compact | 0 | 2 |

Table S3: JONS IBF Measurements and Data

| **Specimen** | **Size (length)** | **Size (thickness)** | **Shape** | **Abrasion** | **Weathering** |
| --- | --- | --- | --- | --- | --- |
| JSV-1 | 0.78 | 0.33 | compact | 3 | 2 |
| JSV-2 | 0.64 | 0.32 | compact | 3 | 3 |
| JSV-3 | 0.38 | 0.14 | compact | 1 | 2 |
| JSV-4 | 0.26 | 0.1 | tabular | 2 | 2 |
| JSV-5 | 0.31 | 0.11 | compact | 2 | 2 |
| JSV-6 | 0.24 | 0.05 | elongate | 1 | 1 |
| JSV-7 | 0.14 | 0.07 | compact | 3 | 2 |
| JSV-8 | 0.15 | 0.09 | compact | 3 | 3 |
| JSV-9 | 0.25 | 0.08 | compact | 3 | 3 |
| JSV-10 | 0.15 | 0.07 | compact | 2 | 2 |
| JSV-11 | 0.09 | 0.09 | compact | 2 | 1 |
| JSV-12 | 0.22 | 0.08 | compact | 3 | 3 |
| JSV-13 | 0.2 | 0.07 | compact | 1 | 1 |
| JSV-14 | 0.14 | 0.08 | compact | 1 | 2 |
| JSV-15 | 0.82 | 0.3 | tabular | 2 | 1 |
| JSV-16 | 0.34 | 0.15 | elongate | 3 | 2 |
| JSV-17 | 0.54 | 0.26 | compact | 3 | 2 |
| JSV-18 | 0.31 | 0.19 | tabular | 2 | 2 |
| JSV-19 | 0.29 | 0.13 | compact | 1 | 3 |
| JSV-20 | 0.51 | 0.3 | compact | 3 | 3 |
| JSV-21 | 0.36 | 0.26 | compact | 3 | 3 |
| JSV-22 | 0.53 | 0.26 | elongate | 3 | 3 |
| JSV-23 | 0.43 | 0.16 | compact | 2 | 3 |
| JSV-24 | 0.62 | 0.31 | elongate | 3 | 0 |
| JSV-25 | 0.77 | 0.22 | elongate | 3 | 3 |
| JSV-26 | 0.34 | 0.18 | elongate | 1 | 3 |
| JSV-27 | 0.47 | 0.25 | compact | 1 | 3 |
| JSV-28 | 0.32 | 0.15 | tabular | 3 | 3 |
| JSV-29 | 0.6 | 0.21 | elongate | 3 | 3 |
| JSV-30 | 0.34 | 0.19 | compact | 2 | 3 |
| JSV-31 | 0.09 | 0.05 | compact | 2 | 3 |
| JSV-32 | 0.06 | 0.04 | compact | 3 | 2 |
| JSV-33 | 0.11 | 0.04 | tabular | 3 | 3 |
| JSV-34 | 0.12 | 0.08 | compact | 2 | 2 |
| JSV-35 | 0.21 | 0.07 | compact | 2 | 3 |
| JSV-36 | 0.22 | 0.08 | compact | 1 | 3 |
| JSV-37 | 0.34 | 0.1 | elongate | 1 | 3 |
| JSV-38 | 0.22 | 0.15 | compact | 0 | 0 |
| JSV-39 | 0.31 | 0.16 | compact | 3 | 3 |
| JSV-40 | 0.24 | 0.1 | tabular | 0 | 3 |
| JSV-41 | 0.18 | 0.1 | tabular | 1 | 3 |
| JSV-42 | 0.3 | 0.09 | compact | 3 | 3 |
| JSV-43 | 0.31 | 0.11 | elongate | 2 | 3 |
| JSV-44 | 0.26 | 0.2 | compact | 1 | 3 |
| JSV-45 | 0.21 | 0.12 | compact | 2 | 3 |
| JSV-46 | 0.23 | 0.09 | compact | 1 | 3 |
| JSV-47 | 0.21 | 0.12 | compact | 2 | 3 |
| JSV-48 | 0.16 | 0.08 | compact | 3 | 3 |
| JSV-49 | 0.18 | 0.09 | compact | 2 | 2 |
| JSV-50 | 0.11 | 0.08 | compact | 3 | 2 |
| JSV-51 | 0.17 | 0.07 | compact | 1 | 2 |
| JSV-52 | 0.2 | 0.12 | compact | 1 | 3 |
| JSV-53 | 0.09 | 0.06 | compact | 3 | 1 |
| JSV-54 | 0.21 | 0.09 | elongate | 2 | 2 |
| JSV-55 | 0.2 | 0.04 | elongate | 1 | 2 |
| JSV-56 | 0.12 | 0.08 | compact | 2 | 3 |
| JSV-57 | 0.19 | 0.1 | compact | 3 | 3 |
| JSV-58 | 0.07 | 0.04 | compact | 3 | 3 |
| JSV-59 | 0.11 | 0.06 | compact | 2 | 2 |
| JSV-60 | 0.12 | 0.06 | compact | 3 | 2 |
| JSV-61 | 0.14 | 0.03 | tabular | 2 | 3 |
| JSV-62 | 0.13 | 0.08 | compact | 3 | 3 |
| JSV-63 | 0.16 | 0.12 | compact | 3 | 3 |
| JSV-64 | 0.18 | 0.02 | tabular | 2 | 2 |
| JSV-65 | 0.15 | 0.09 | elongate | 3 | 3 |
| JSV-66 | 0.14 | 0.08 | compact | 2 | 2 |
| JSV-67 | 0.08 | 0.06 | compact | 1 | 3 |
| JSV-68 | 0.07 | 0.04 | compact | 1 | 3 |
| JSV-69 | 0.18 | 0.08 | compact | 1 | 2 |
| JSV-70 | 0.07 | 0.06 | compact | 3 | 2 |
| JSV-71 | 0.25 | 0.02 | tabular | 0 | 3 |
| JSV-72 | 0.09 | 0.03 | compact | 3 | 2 |
| JSV-73 | 0.11 | 0.04 | tabular | 2 | 3 |
| JSV-74 | 0.15 | 0.1 | compact | 2 | 2 |
| JSV-75 | 0.07 | 0.02 | compact | 3 | 3 |
| JSV-76 | 0.15 | 0.08 | compact | 2 | 2 |
| JSV-77 | 0.11 | 0.06 | compact | 3 | 2 |
| JSV-78 | 0.11 | 0.03 | tabular | 1 | 3 |
| JSV-79 | 0.12 | 0.01 | tabular | 1 | 3 |
| JSV-80 | 0.15 | 0.08 | compact | 2 | 2 |
| JSV-81 | 0.08 | 0.06 | compact | 1 | 2 |
| JSV-82 | 0.08 | 0.04 | compact | 2 | 3 |
| JSV-83 | 0.18 | 0.04 | compact | 1 | 2 |
| JSV-84 | 0.07 | 0.06 | compact | 3 | 1 |
| JSV-85 | 0.2 | 0.12 | compact | 1 | 3 |
| JSV-86 | 0.09 | 0.06 | compact | 3 | 1 |
| JSV-87 | 0.21 | 0.09 | elongate | 2 | 2 |
| JSV-88 | 0.18 | 0.04 | elongate | 1 | 2 |
| JSV-89 | 0.19 | 0.08 | compact | 2 | 3 |
| JSV-90 | 0.2 | 0.1 | compact | 1 | 3 |
| JSV-91 | 0.07 | 0.05 | compact | 3 | 2 |
| JSV-92 | 0.12 | 0.08 | compact | 2 | 2 |
| JSV-93 | 0.21 | 0.07 | compact | 2 | 1 |
| JSV-94 | 0.23 | 0.08 | compact | 1 | 3 |
| JSV-95 | 0.64 | 0.33 | compact | 3 | 3 |
| JSV-96 | 0.38 | 0.14 | compact | 2 | 1 |
| JSV-97 | 0.26 | 0.1 | tabular | 2 | 2 |
| JSV-98 | 0.24 | 0.11 | compact | 3 | 2 |
| JSV-99 | 0.27 | 0.06 | elongate | 1 | 2 |
| JSV-100 | 0.31 | 0.09 | tabular | 2 | 3 |
| JSV-101 | 0.11 | 0.06 | compact | 3 | 2 |
| JSV-102 | 0.29 | 0.13 | compact | 0 | 3 |
| JSV-103 | 0.16 | 0.09 | compact | 2 | 2 |
| JSV-104 | 0.17 | 0.09 | compact | 2 | 2 |
| JSV-105 | 0.07 | 0.06 | compact | 3 | 2 |
| JSV-106 | 0.16 | 0.09 | compact | 2 | 2 |
| JSV-107 | 0.11 | 0.04 | compact | 2 | 3 |
| JSV-108 | 0.11 | 0.03 | elongate | 1 | 2 |
| JSV-109 | 0.22 | 0.04 | elongate | 1 | 1 |
| JSV-110 | 0.14 | 0.06 | tabular | 0 | 0 |
| JSV-111 | 0.18 | 0.1 | compact | 2 | 3 |
| JSV-112 | 0.32 | 0.17 | compact | 0 | 3 |
| JSV-113 | 0.13 | 0.06 | tabular | 3 | 3 |
| JSV-114 | 0.11 | 0.05 | compact | 2 | 2 |
| JSV-115 | 0.08 | 0.04 | compact | 3 | 2 |
| JSV-116 | 0.06 | 0.05 | compact | 2 | 2 |
| JSV-117 | 0.08 | 0.07 | compact | 2 | 2 |
| JSV-118 | 0.22 | 0.06 | tabular | 1 | 3 |
| JSV-119 | 0.17 | 0.06 | tabular | 3 | 1 |
| JSV-120 | 0.07 | 0.03 | compact | 1 | 1 |
| JSV-121 | 0.12 | 0.07 | compact | 2 | 3 |
| JSV-122 | 0.08 | 0.03 | tabular | 1 | 2 |
| JSV-123 | 0.11 | 0.05 | compact | 2 | 2 |
| JSV-124 | 0.17 | 0.09 | compact | 1 | 1 |
| JSV-125 | 0.14 | 0.1 | compact | 3 | 3 |
| JSV-126 | 0.14 | 0.09 | compact | 3 | 3 |
| JSV-127 | 0.06 | 0.01 | compact | 3 | 2 |
| JSV-128 | 0.08 | 0.04 | compact | 3 | 3 |
| JSV-129 | 0.09 | 0.01 | tabular | 1 | 3 |
| JSV-130 | 0.07 | 0.02 | tabular | 1 | 3 |
| JSV-131 | 0.1 | 0.03 | compact | 2 | 2 |
| JSV-132 | 0.1 | 0.04 | compact | 3 | 3 |
| JSV-133 | 12 | 0.02 | elongate | 1 | 2 |
| JSV-134 | 0.14 | 0.04 | elongate | 0 | 1 |
| JSV-135 | 0.1 | 0.05 | compact | 1 | 3 |
| JSV-136 | 0.09 | 0.05 | compact | 1 | 3 |
| JSV-137 | 0.13 | 0.04 | tabular | 2 | 2 |
| JSV-138 | 0.06 | 0.01 | tabular | 2 | 2 |
| JSV-139 | 0.1 | 0.08 | compact | 1 | 3 |
| JSV-140 | 0.07 | 0.02 | compact | 0 | 3 |
| JSV-141 | 0.12 | 0.06 | elongate | 1 | 2 |
| JSV-142 | 0.11 | 0.06 | compact | 2 | 2 |
| JSV-143 | 0.15 | 0.03 | tabular | 0 | 2 |
| JSV-144 | 0.09 | 0.06 | tabular | 1 | 2 |
| JSV-145 | 0.09 | 0.05 | compact | 2 | 3 |
| JSV-146 | 0.08 | 0.06 | compact | 3 | 2 |
| JSV-147 | 0.07 | 0.03 | compact | 2 | 2 |
| JSV-148 | 0.06 | 0.03 | compact | 3 | 2 |
| JSV-149 | 0.07 | 0.05 | compact | 2 | 3 |
| JSV-150 | 0.12 | 0.06 | elongate | 1 | 3 |
| JSV-151 | 0.04 | 0.02 | compact | 2 | 2 |
| JSV-152 | 0.06 | 0.05 | compact | 2 | 2 |
| JSV-153 | 0.08 | 0.03 | compact | 1 | 2 |
| JSV-154 | 0.12 | 0.02 | tabular | 1 | 2 |
| JSV-155 | 0.08 | 0.05 | compact | 2 | 3 |
| JSV-156 | 0.17 | 0.05 | conical | 0 | 2 |
| JSV-157 | 0.13 | 0.08 | compact | 1 | 3 |
| JSV-158 | 0.11 | 0.09 | compact | 1 | 3 |
| JSV-159 | 0.1 | 0.02 | tabular | 2 | 2 |
| JSV-160 | 0.09 | 0.07 | compact | 2 | 2 |
| JSV-161 | 0.09 | 0.05 | compact | 1 | 3 |
| JSV-162 | 0.17 | 0.07 | compact | 3 | 3 |
| JSV-163 | 0.13 | 0.05 | compact | 2 | 3 |
| JSV-164 | 0.09 | 0.06 | compact | 1 | 3 |
| JSV-165 | 0.18 | 0.04 | elongate | 1 | 3 |
| JSV-166 | 0.05 | 0.03 | compact | 2 | 3 |
| JSV-167 | 0.09 | 0.05 | compact | 2 | 3 |
| JSV-168 | 0.12 | 0.02 | tabular | 3 | 2 |
| JSV-169 | 0.14 | 0.03 | tabular | 1 | 3 |
| JSV-170 | 0.13 | 0.08 | compact | 2 | 3 |
| JSV-171 | 0.07 | 0.05 | compact | 3 | 2 |
| JSV-172 | 0.08 | 0.03 | compact | 1 | 3 |
| JSV-173 | 0.08 | 0.04 | compact | 2 | 3 |
| JSV-174 | 0.05 | 0.04 | compact | 2 | 3 |
| JSV-175 | 0.13 | 0.07 | compact | 1 | 2 |
| JSV-176 | 0.09 | 0.05 | compact | 1 | 1 |
| JSV-177 | 0.09 | 0.03 | compact | 2 | 2 |
| JSV-178 | 0.06 | 0.07 | compact | 3 | 3 |
| JSV-179 | 0.08 | 0.04 | compact | 2 | 3 |
| JSV-180 | 0.07 | 0.05 | compact | 1 | 3 |
| JSV-181 | 0.05 | 0.04 | compact | 1 | 2 |
| JSV-182 | 0.09 | 0.06 | compact | 2 | 2 |
| JSV-183 | 0.12 | 0.04 | compact | 1 | 3 |
| JSV-184 | 0.09 | 0.04 | compact | 2 | 3 |
| JSV-185 | 0.05 | 0.03 | compact | 3 | 2 |
| JSV-186 | 0.08 | 0.05 | compact | 1 | 3 |
| JSV-187 | 0.1 | 0.03 | compact | 2 | 2 |
| JSV-188 | 0.07 | 0.06 | compact | 3 | 2 |
| JSV-189 | 0.06 | 0.04 | compact | 3 | 3 |
| JSV-190 | 0.11 | 0.06 | compact | 2 | 3 |
| JSV-191 | 0.07 | 0.06 | compact | 2 | 3 |
| JSV-192 | 0.11 | 0.04 | compact | 1 | 2 |
| JSV-193 | 0.07 | 0.03 | tabular | 2 | 2 |
| JSV-194 | 0.05 | 0.04 | compact | 3 | 3 |
| JSV-195 | 0.07 | 0.04 | compact | 3 | 3 |
| JSV-196 | 0.2 | 0.07 | elongate | 2 | 3 |
| JSV-197 | 0.09 | 0.06 | compact | 2 | 2 |
| JSV-198 | 0.09 | 0.04 | compact | 1 | 3 |
| JSV-199 | 0.2 | 0.09 | tabular | 1 | 3 |
| JSV-200 | 0.12 | 0.05 | compact | 1 | 3 |
| JSV-201 | 0.13 | 0.11 | compact | 2 | 3 |
| JSV-202 | 0.7 | 0.07 | compact | 3 | 3 |
| JSV-203 | 0.07 | 0.03 | compact | 3 | 2 |
| JSV-204 | 0.09 | 0.05 | compact | 2 | 2 |
| JSV-205 | 0.12 | 0.02 | tabular | 1 | 1 |
| JSV-206 | 0.08 | 0.04 | compact | 1 | 3 |
| JSV-207 | 0.11 | 0.06 | compact | 2 | 3 |
| JSV-208 | 0.1 | 0.07 | compact | 3 | 2 |
| JSV-209 | 0.09 | 0.05 | compact | 2 | 3 |
| JSV-210 | 0.11 | 0.07 | compact | 3 | 3 |
| JSV-211 | 0.08 | 0.06 | compact | 2 | 3 |
| JSV-212 | 0.13 | 0.05 | compact | 3 | 3 |
| JSV-213 | 0.06 | 0.05 | compact | 1 | 1 |
| JSV-214 | 0.08 | 0.06 | compact | 3 | 0 |
| JSV-215 | 0.08 | 0.06 | compact | 2 | 2 |
| JSV-216 | 0.07 | 0.05 | compact | 1 | 3 |
| JSV-217 | 0.11 | 0.07 | compact | 1 | 3 |
| JSV-218 | 0.08 | 0.04 | compact | 2 | 3 |
| JSV-219 | 0.08 | 0.05 | compact | 2 | 3 |
| JSV-220 | 0.05 | 0.06 | compact | 2 | 2 |
| JSV-221 | 0.08 | 0.06 | compact | 1 | 2 |
| JSV-222 | 0.08 | 0.05 | compact | 1 | 3 |
| JSV-223 | 0.11 | 0.07 | compact | 1 | 3 |
| JSV-224 | 0.06 | 0.05 | compact | 3 | 3 |
| JSV-225 | 0.11 | 0.05 | compact | 2 | 3 |
| JSV-226 | 0.13 | 0.06 | compact | 0 | 3 |
| JSV-227 | 0.08 | 0.06 | compact | 1 | 2 |
| JSV-228 | 0.1 | 0.06 | compact | 2 | 2 |
| JSV-229 | 0.09 | 0.05 | compact | 3 | 2 |
| JSV-230 | 0.12 | 0.06 | elongate | 0 | 3 |
| JSV-231 | 0.09 | 0.03 | tabular | 1 | 1 |
| JSV-232 | 0.16 | 0.07 | compact | 3 | 3 |
| JSV-233 | 0.18 | 0.06 | elongate | 0 | 3 |
| JSV-234 | 0.1 | 0.07 | compact | 1 | 2 |
| JSV-235 | 0.12 | 0.07 | compact | 2 | 3 |
| JSV-236 | 0.11 | 0.08 | compact | 2 | 1 |
| JSV-237 | 0.19 | 0.14 | conical | 0 | 2 |
| JSV-238 | 0.2 | 0.09 | compact | 0 | 3 |
| JSV-239 | 0.08 | 0.05 | compact | 2 | 3 |
| JSV-240 | 0.09 | 0.07 | compact | 3 | 3 |
| JSV-241 | 0.15 | 0.06 | compact | 2 | 3 |
| JSV-242 | 0.19 | 0.05 | compact | 2 | 3 |
| JSV-243 | 0.09 | 0.09 | compact | 2 | 1 |
| JSV-244 | 0.18 | 0.05 | compact | 1 | 3 |
| JSV-245 | 0.14 | 0.08 | compact | 2 | 3 |
| JSV-246 | 0.11 | 0.06 | compact | 2 | 3 |
| JSV-247 | 0.1 | 0.04 | compact | 2 | 3 |
| JSV-248 | 0.11 | 0.08 | compact | 2 | 2 |
| JSV-249 | 0.11 | 0.04 | compact | 1 | 3 |
| JSV-250 | 0.2 | 0.11 | compact | 1 | 3 |
| JSV-251 | 0.12 | 0.09 | compact | 0 | 3 |
| JSV-252 | 0.07 | 0.06 | compact | 2 | 3 |
| JSV-253 | 0.09 | 0.04 | tabular | 0 | 2 |
| JSV-254 | 0.12 | 0.07 | tabular | 1 | 3 |
| JSV-255 | 0.12 | 0.07 | compact | 1 | 3 |
| JSV-256 | 0.05 | 0.04 | compact | 2 | 3 |
| JSV-257 | 0.14 | 0.04 | tabular | 2 | 3 |
| JSV-258 | 0.08 | 0.02 | compact | 2 | 2 |
| JSV-259 | 0.09 | 0.07 | compact | 2 | 2 |
| JSV-260 | 0.09 | 0.07 | compact | 1 | 2 |
| JSV-261 | 0.13 | 0.07 | compact | 2 | 0 |
| JSV-262 | 0.11 | 0.06 | compact | 2 | 1 |
| JSV-263 | 0.07 | 0.04 | compact | 3 | 2 |
| JSV-264 | 0.09 | 0.06 | compact | 1 | 3 |
| JSV-265 | 0.15 | 0.06 | compact | 1 | 3 |
| JSV-266 | 0.1 | 0.05 | compact | 3 | 3 |
| JSV-267 | 0.09 | 0.06 | compact | 3 | 3 |
| JSV-268 | 0.08 | 0.08 | compact | 3 | 2 |
| JSV-269 | 0.1 | 0.08 | compact | 2 | 3 |
| JSV-270 | 0.16 | 0.03 | compact | 1 | 2 |
| JSV-271 | 0.1 | 0.02 | compact | 1 | 3 |
| JSV-272 | 0.05 | 0.02 | tabular | 2 | 3 |
| JSV-273 | 0.15 | 0.03 | elongate | 2 | 2 |
| JSV-274 | 0.06 | 0.05 | compact | 2 | 2 |
| JSV-275 | 0.13 | 0.04 | compact | 2 | 2 |
| JSV-276 | 0.14 | 0.07 | tabular | 2 | 3 |
| JSV-277 | 0.13 | 0.07 | compact | 1 | 3 |
| JSV-278 | 0.14 | 0.04 | elongate | 1 | 3 |
| JSV-279 | 0.15 | 0.01 | elongate | 0 | 0 |
| JSV-280 | 0.18 | 0.06 | elongate | 1 | 1 |
| JSV-281 | 0.07 | 0.03 | tabular | 2 | 3 |
| JSV-282 | 0.09 | 0.06 | compact | 3 | 2 |
| JSV-283 | 0.11 | 0.07 | compact | 1 | 0 |
| JSV-284 | 0.12 | 0.07 | compact | 1 | 2 |
| JSV-285 | 0.08 | 0.06 | compact | 3 | 2 |
| JSV-286 | 0.1 | 0.054 | compact | 3 | 3 |
| JSV-287 | 0.08 | 0.06 | compact | 1 | 2 |
| JSV-288 | 0.06 | 0.03 | tabular | 2 | 1 |
| JSV-289 | 0.09 | 0.03 | tabular | 3 | 2 |
| JSV-290 | 0.06 | 0.04 | compact | 1 | 2 |
| JSV-291 | 0.14 | 0.03 | tabular | 1 | 3 |
| JSV-292 | 0.11 | 0.03 | elongate | 1 | 3 |
| JSV-293 | 0.06 | 0.04 | compact | 1 | 3 |
| JSV-294 | 0.09 | 0.03 | elongate | 2 | 3 |
| JSV-295 | 0.12 | 0.07 | compact | 2 | 2 |
| JSV-296 | 0.16 | 0.04 | compact | 2 | 2 |
| JSV-297 | 0.08 | 0.04 | compact | 2 | 1 |
| JSV-298 | 0.08 | 0.05 | compact | 3 | 2 |
| JSV-299 | 0.06 | 0.04 | compact | 3 | 3 |
| JSV-300 | 0.09 | 0.03 | compact | 2 | 3 |
| JSV-301 | 0.13 | 0.03 | elongate | 1 | 1 |
| JSV-302 | 0.1 | 0.03 | tabular | 1 | 2 |
| JSV-303 | 0.09 | 0.03 | compact | 2 | 2 |
| JSV-304 | 0.07 | 0.04 | compact | 2 | 2 |
| JSV-305 | 0.05 | 0.03 | compact | 2 | 2 |
| JSV-306 | 0.07 | 0.04 | compact | 2 | 2 |
| JSV-307 | 0.12 | 0.02 | tabular | 1 | 2 |
| JSV-308 | 0.08 | 0.3 | compact | 1 | 2 |
| JSV-309 | 0.05 | 0.02 | compact | 3 | 3 |
| JSV-310 | 0.07 | 0.04 | compact | 3 | 3 |
| JSV-311 | 0.09 | 0.07 | compact | 1 | 2 |
| JSV-312 | 0.1 | 0.07 | compact | 2 | 0 |
| JSV-313 | 0.11 | 0.06 | compact | 3 | 1 |
| JSV-314 | 0.06 | 0.04 | compact | 2 | 2 |
| JSV-315 | 0.09 | 0.06 | compact | 1 | 3 |
| JSV-316 | 0.11 | 0.05 | compact | 2 | 2 |
| JSV-317 | 0.07 | 0.05 | compact | 1 | 3 |
| JSV-318 | 0.05 | 0.04 | compact | 1 | 3 |
| JSV-319 | 0.09 | 0.05 | compact | 1 | 2 |
| JSV-320 | 0.05 | 0.05 | compact | 3 | 2 |
| JSV-321 | 0.07 | 0.05 | compact | 2 | 2 |
| JSV-322 | 0.1 | 0.06 | compact | 2 | 2 |
| JSV-323 | 0.11 | 0.07 | compact | 3 | 1 |
| JSV-324 | 0.09 | 0.04 | compact | 2 | 1 |
| JSV-325 | 0.1 | 0.06 | tabular | 2 | 3 |
| JSV-326 | 0.04 | 0.03 | compact | 1 | 3 |
| JSV-327 | 0.1 | 0.03 | tabular | 2 | 3 |
| JSV-328 | 0.07 | 0.03 | tabular | 2 | 2 |
| JSV-329 | 0.1 | 0.04 | compact | 3 | 3 |
| JSV-330 | 0.13 | 0.06 | compact | 2 | 3 |
| JSV-331 | 0.06 | 0.05 | compact | 2 | 3 |
| JSV-332 | 0.06 | 0.04 | compact | 1 | 2 |
| JSV-333 | 0.08 | 0.04 | compact | 0 | 1 |
| JSV-334 | 0.07 | 0.05 | compact | 1 | 2 |
| JSV-335 | 0.07 | 0.05 | compact | 2 | 2 |
| JSV-336 | 0.1 | 0.06 | compact | 2 | 2 |
| JSV-337 | 0.14 | 0.06 | compact | 3 | 3 |
| JSV-338 | 0.1 | 0.04 | compact | 1 | 0 |
| JSV-339 | 0.1 | 0.02 | elongate | 0 | 1 |
| JSV-340 | 0.06 | 0.08 | compact | 2 | 1 |
| JSV-341 | 0.17 | 0.05 | compact | 1 | 2 |
| JSV-342 | 0.06 | 0.04 | compact | 3 | 3 |
| JSV-343 | 0.09 | 0.07 | compact | 2 | 3 |
| JSV-344 | 0.09 | 0.01 | tabular | 2 | 2 |
| JSV-345 | 0.1 | 0.07 | compact | 1 | 1 |
| JSV-346 | 0.11 | 0.05 | compact | 3 | 3 |
| JSV-347 | 0.09 | 0.06 | compact | 2 | 3 |
| JSV-348 | 0.11 | 0.05 | compact | 1 | 2 |
| JSV-349 | 0.06 | 0.03 | compact | 2 | 2 |
| JSV-350 | 0.12 | 0.06 | compact | 2 | 3 |
| JSV-351 | 0.09 | 0.05 | compact | 1 | 3 |
| JSV-352 | 0.08 | 0.05 | compact | 1 | 3 |
| JSV-353 | 0.05 | 0.03 | compact | 0 | 1 |
| JSV-354 | 0.06 | 0.03 | compact | 1 | 2 |
| JSV-355 | 0.07 | 0.03 | compact | 2 | 3 |
| JSV-356 | 0.07 | 0.02 | tabular | 1 | 1 |
| JSV-357 | 0.08 | 0.02 | compact | 0 | 3 |
| JSV-358 | 0.08 | 0.03 | compact | 1 | 3 |
| JSV-359 | 0.07 | 0.03 | compact | 3 | 3 |
| JSV-360 | 0.08 | 0.02 | compact | 1 | 2 |
| JSV-361 | 0.11 | 0.03 | tabular | 2 | 2 |
| JSV-362 | 0.05 | 0.04 | compact | 2 | 3 |
| JSV-363 | 0.08 | 0.03 | compact | 2 | 1 |
| JSV-364 | 0.06 | 0.03 | compact | 2 | 3 |
| JSV-365 | 0.05 | 0.03 | compact | 3 | 3 |
| JSV-366 | 0.07 | 0.03 | compact | 2 | 3 |
| JSV-367 | 0.07 | 0.02 | tabular | 3 | 2 |
| JSV-368 | 0.06 | 0.02 | compact | 1 | 2 |
| JSV-369 | 0.08 | 0.03 | tabular | 2 | 2 |
| JSV-370 | 0.08 | 0.04 | compact | 2 | 3 |
| JSV-371 | 0.07 | 0.05 | compact | 2 | 2 |
| JSV-372 | 0.06 | 0.03 | compact | 3 | 2 |
| JSV-373 | 0.12 | 0.07 | elongate | 2 | 2 |
| JSV-374 | 0.11 | 0.05 | elongate | 1 | 3 |
| JSV-375 | 0.07 | 0.04 | compact | 1 | 2 |
| JSV-376 | 0.06 | 0.02 | tabular | 2 | 2 |
| JSV-377 | 0.08 | 0.03 | compact | 2 | 2 |
| JSV-378 | 0.08 | 0.05 | compact | 3 | 3 |
| JSV-379 | 0.08 | 0.03 | compact | 2 | 3 |
| JSV-380 | 0.14 | 0.07 | compact | 3 | 3 |
| JSV-381 | 0.08 | 0.04 | compact | 2 | 2 |
| JSV-382 | 0.09 | 0.02 | tabular | 1 | 2 |
| JSV-383 | 0.1 | 0.04 | compact | 1 | 3 |
| JSV-384 | 0.09 | 0.06 | compact | 2 | 3 |
| JSV-385 | 0.07 | 0.03 | tabular | 2 | 1 |
| JSV-386 | 0.07 | 0.03 | compact | 3 | 3 |
| JSV-387 | 0.14 | 0.05 | elongate | 1 | 2 |
| JSV-388 | 0.08 | 0.05 | compact | 3 | 3 |
| JSV-389 | 0.07 | 0.02 | tabular | 2 | 3 |
| JSV-390 | 0.08 | 0.05 | compact | 2 | 3 |
| JSV-391 | 0.1 | 0.04 | tabular | 3 | 3 |
| JSV-392 | 0.08 | 0.03 | compact | 2 | 3 |
| JSV-393 | 0.07 | 0.03 | compact | 3 | 2 |
| JSV-394 | 0.09 | 0.07 | compact | 1 | 2 |
| JSV-395 | 0.09 | 0.03 | compact | 2 | 2 |
| JSV-396 | 0.06 | 0.03 | compact | 3 | 3 |
| JSV-397 | 0.07 | 0.03 | compact | 3 | 3 |
| JSV-398 | 0.06 | 0.03 | compact | 2 | 2 |
| JSV-399 | 0.11 | 0.05 | compact | 3 | 3 |
| JSV-400 | 0.08 | 0.03 | tabular | 3 | 3 |
| JSV-401 | 0.07 | 0.05 | compact | 3 | 3 |
| JSV-402 | 0.09 | 0.03 | tabular | 3 | 2 |
| JSV-403 | 0.06 | 0.03 | compact | 2 | 1 |
| JSV-404 | 0.12 | 0.04 | tabular | 2 | 2 |
| JSV-405 | 0.06 | 0.02 | compact | 3 | 3 |
| JSV-406 | 0.11 | 0.06 | compact | 2 | 3 |
| JSV-407 | 0.05 | 0.05 | compact | 2 | 3 |
| JSV-408 | 0.09 | 0.03 | tabular | 3 | 3 |
| JSV-409 | 0.09 | 0.03 | compact | 2 | 3 |
| JSV-410 | 0.07 | 0.04 | compact | 2 | 3 |
| JSV-411 | 0.06 | 0.05 | compact | 3 | 3 |
| JSV-412 | 0.07 | 0.02 | tabular | 3 | 3 |
| JSV-413 | 0.08 | 0.04 | compact | 2 | 3 |
| JSV-414 | 0.06 | 0.02 | compact | 2 | 3 |
| JSV-415 | 0.05 | 0.04 | compact | 2 | 2 |
| JSV-416 | 0.06 | 0.05 | compact | 3 | 3 |
| JSV-417 | 0.07 | 0.06 | compact | 2 | 3 |
| JSV-418 | 0.06 | 0.02 | compact | 3 | 3 |
| JSV-419 | 0.08 | 0.03 | compact | 2 | 2 |
| JSV-420 | 0.04 | 0.04 | compact | 2 | 3 |
| JSV-421 | 0.07 | 0.04 | compact | 3 | 3 |
| JSV-422 | 0.03 | 0.03 | compact | 3 | 3 |
| JSV-423 | 0.06 | 0.03 | compact | 2 | 1 |
| JSV-424 | 0.07 | 0.05 | compact | 3 | 2 |
| JSV-425 | 0.05 | 0.03 | compact | 2 | 3 |
| JSV-426 | 0.08 | 0.04 | tabular | 1 | 0 |
| JSV-427 | 0.07 | 0.03 | compact | 2 | 2 |
| JSV-428 | 0.07 | 0.06 | compact | 2 | 2 |
| JSV-429 | 0.03 | 0.02 | compact | 1 | 3 |
| JSV-430 | 0.09 | 0.04 | compact | 1 | 3 |
| JSV-431 | 0.06 | 0.03 | compact | 2 | 2 |
| JSV-432 | 0.08 | 0.02 | tabular | 1 | 3 |
| JSV-433 | 0.06 | 0.03 | compact | 2 | 3 |
| JSV-434 | 0.06 | 0.03 | compact | 3 | 3 |
| JSV-435 | 0.1 | 0.05 | compact | 1 | 3 |
| JSV-436 | 0.08 | 0.04 | compact | 2 | 2 |
| JSV-437 | 0.08 | 0.05 | compact | 3 | 2 |
| JSV-438 | 0.07 | 0.04 | compact | 3 | 3 |
| JSV-439 | 0.08 | 0.04 | compact | 1 | 3 |
| JSV-440 | 0.09 | 0.04 | compact | 2 | 3 |
| JSV-441 | 0.07 | 0.04 | compact | 2 | 3 |
| JSV-442 | 0.09 | 0.02 | tabular | 1 | 3 |
| JSV-443 | 0.09 | 0.05 | compact | 2 | 2 |
| JSV-444 | 0.08 | 0.04 | compact | 1 | 3 |
| JSV-445 | 0.09 | 0.05 | compact | 1 | 2 |
| JSV-446 | 0.06 | 0.02 | compact | 1 | 2 |
| JSV-447 | 0.08 | 0.02 | tabular | 0 | 3 |
| JSV-448 | 0.08 | 0.03 | tabular | 1 | 3 |
| JSV-449 | 0.09 | 0.04 | compact | 1 | 3 |
| JSV-450 | 0.05 | 0.03 | compact | 3 | 2 |
| JSV-451 | 0.09 | 0.02 | compact | 2 | 2 |
| JSV-452 | 0.07 | 0.03 | compact | 2 | 3 |
| JSV-453 | 0.06 | 0.03 | compact | 2 | 3 |
| JSV-454 | 0.08 | 0.04 | compact | 1 | 3 |
| JSV-455 | 0.08 | 0.03 | compact | 0 | 2 |
| JSV-456 | 0.11 | 0.05 | compact | 2 | 3 |
| JSV-457 | 0.1 | 0.05 | compact | 1 | 3 |
| JSV-458 | 0.08 | 0.04 | compact | 2 | 3 |
| JSV-459 | 0.07 | 0.02 | compact | 1 | 3 |
| JSV-460 | 0.07 | 0.03 | compact | 2 | 3 |
| JSV-461 | 0.07 | 0.03 | compact | 3 | 2 |
| JSV-462 | 0.06 | 0.02 | compact | 2 | 2 |
| JSV-463 | 0.06 | 0.05 | compact | 1 | 3 |
| JSV-464 | 0.09 | 0.04 | compact | 2 | 3 |
| JSV-465 | 0.05 | 0.03 | compact | 2 | 3 |
| JSV-466 | 0.06 | 0.01 | compact | 2 | 3 |
| JSV-467 | 0.08 | 0.04 | compact | 2 | 3 |
| JSV-468 | 0.07 | 0.03 | compact | 2 | 2 |
| JSV-469 | 0.08 | 0.05 | compact | 2 | 3 |
| JSV-470 | 0.07 | 0.03 | compact | 3 | 3 |
| JSV-471 | 0.08 | 0.03 | tabular | 1 | 1 |
| JSV-472 | 0.12 | 0.05 | compact | 0 | 3 |
| JSV-473 | 0.05 | 0.02 | compact | 2 | 2 |
| JSV-474 | 0.05 | 0.03 | compact | 2 | 2 |
| JSV-475 | 0.06 | 0.04 | compact | 3 | 3 |
| JSV-476 | 0.06 | 0.04 | compact | 3 | 3 |
| JSV-477 | 0.07 | 0.03 | tabular | 3 | 3 |
| JSV-478 | 0.06 | 0.03 | compact | 3 | 2 |
| JSV-479 | 0.07 | 0.03 | compact | 1 | 1 |
| JSV-480 | 0.07 | 0.04 | compact | 3 | 3 |
| JSV-481 | 0.05 | 0.05 | compact | 3 | 3 |
| JSV-482 | 0.07 | 0.02 | compact | 3 | 3 |
| JSV-483 | 0.05 | 0.03 | compact | 3 | 2 |
| JSV-484 | 0.06 | 0.04 | compact | 3 | 3 |
| JSV-485 | 0.06 | 0.02 | tabular | 2 | 3 |
| JSV-486 | 0.07 | 0.03 | compact | 3 | 3 |
| JSV-487 | 0.1 | 0.03 | elongate | 2 | 3 |
| JSV-488 | 0.06 | 0.02 | compact | 3 | 3 |
| JSV-489 | 0.1 | 0.02 | tabular | 2 | 3 |
| JSV-490 | 0.11 | 0.03 | tabular | 3 | 2 |
| JSV-491 | 0.07 | 0.02 | compact | 3 | 2 |
| JSV-492 | 0.06 | 0.04 | compact | 3 | 1 |
| JSV-493 | 0.08 | 0.06 | compact | 3 | 2 |
| JSV-494 | 0.11 | 0.01 | tabular | 2 | 1 |
| JSV-495 | 0.06 | 0.02 | tabular | 1 | 2 |
| JSV-496 | 0.06 | 0.03 | compact | 2 | 2 |
| JSV-497 | 0.06 | 0.04 | compact | 1 | 2 |
| JSV-498 | 0.08 | 0.05 | compact | 3 | 3 |
| JSV-499 | 0.07 | 0.04 | compact | 3 | 3 |
| JSV-500 | 0.1 | 0.05 | tabular | 2 | 2 |
| JSV-501 | 0.06 | 0.02 | compact | 1 | 3 |
| JSV-502 | 0.09 | 0.04 | compact | 3 | 2 |
| JSV-503 | 0.07 | 0.03 | compact | 2 | 2 |
| JSV-504 | 0.06 | 0.04 | compact | 2 | 2 |
| JSV-505 | 0.06 | 0.06 | compact | 3 | 3 |
| JSV-506 | 0.07 | 0.03 | compact | 1 | 3 |
| JSV-507 | 0.14 | 0.05 | elongate | 0 | 3 |
| JSV-508 | 0.06 | 0.03 | compact | 2 | 3 |
| JSV-509 | 0.06 | 0.03 | compact | 2 | 3 |
| JSV-510 | 0.07 | 0.05 | compact | 3 | 3 |
| JSV-511 | 0.06 | 0.06 | compact | 3 | 3 |
| JSV-512 | 0.07 | 0.05 | compact | 2 | 2 |
| JSV-513 | 0.06 | 0.05 | compact | 2 | 2 |
| JSV-514 | 0.1 | 0.03 | tabular | 1 | 1 |
| JSV-515 | 0.09 | 0.04 | compact | 1 | 2 |
| JSV-516 | 0.04 | 0.03 | compact | 2 | 3 |
| JSV-517 | 0.06 | 0.04 | compact | 2 | 3 |
| JSV-518 | 0.07 | 0.02 | compact | 2 | 3 |
| JSV-519 | 0.05 | 0.03 | compact | 2 | 3 |
| JSV-520 | 0.05 | 0.04 | compact | 2 | 3 |
| JSV-521 | 0.1 | 0.07 | compact | 1 | 3 |
| JSV-522 | 0.08 | 0.05 | compact | 3 | 2 |
| JSV-523 | 0.09 | 0.05 | compact | 0 | 1 |
| JSV-524 | 0.06 | 0.03 | tabular | 1 | 1 |
| JSV-525 | 0.05 | 0.04 | compact | 3 | 3 |
| JSV-526 | 0.06 | 0.05 | compact | 3 | 3 |
| JSV-527 | 0.11 | 0.03 | compact | 2 | 3 |
| JSV-528 | 0.09 | 0.03 | tabular | 2 | 2 |
| JSV-529 | 0.11 | 0.04 | elongate | 1 | 2 |
| JSV-530 | 0.12 | 0.03 | elongate | 2 | 3 |
| JSV-531 | 0.1 | 0.05 | compact | 2 | 3 |
| JSV-532 | 0.08 | 0.03 | compact | 2 | 3 |
| JSV-533 | 0.12 | 0.03 | compact | 2 | 3 |
| JSV-534 | 0.08 | 0.04 | compact | 1 | 1 |
| JSV-535 | 0.1 | 0.05 | compact | 0 | 2 |
| JSV-536 | 0.08 | 0.05 | compact | 1 | 3 |
| JSV-537 | 0.08 | 0.05 | compact | 2 | 2 |
| JSV-538 | 0.06 | 0.04 | compact | 3 | 3 |
| JSV-539 | 0.05 | 0.06 | compact | 3 | 3 |
| JSV-540 | 0.1 | 0.03 | tabular | 2 | 3 |
| JSV-541 | 0.06 | 0.03 | compact | 1 | 2 |
| JSV-542 | 0.06 | 0.02 | compact | 0 | 3 |
| JSV-543 | 0.07 | 0.02 | compact | 3 | 3 |
| JSV-544 | 0.06 | 0.03 | tabular | 1 | 3 |
| JSV-545 | 0.1 | 0.04 | compact | 2 | 2 |
| JSV-546 | 0.07 | 0.01 | compact | 1 | 2 |
| JSV-547 | 0.07 | 0.05 | compact | 3 | 3 |
| JSV-548 | 0.06 | 0.02 | tabular | 2 | 2 |
| JSV-549 | 0.06 | 0.03 | compact | 3 | 2 |
| JSV-550 | 0.05 | 0.03 | compact | 3 | 3 |
| JSV-551 | 0.08 | 0.03 | compact | 1 | 2 |
| JSV-552 | 0.06 | 0.02 | tabular | 2 | 3 |
| JSV-553 | 0.05 | 0.05 | compact | 2 | 2 |
| JSV-554 | 0.05 | 0.04 | compact | 3 | 2 |
| JSV-555 | 0.07 | 0.02 | tabular | 2 | 3 |
| JSV-556 | 0.06 | 0.04 | compact | 1 | 2 |
| JSV-557 | 0.06 | 0.03 | compact | 1 | 3 |
| JSV-558 | 0.07 | 0.03 | tabular | 3 | 3 |
| JSV-559 | 0.05 | 0.02 | tabular | 2 | 1 |
| JSV-560 | 0.05 | 0.04 | compact | 3 | 3 |
| JSV-561 | 0.06 | 0.04 | compact | 1 | 3 |
| JSV-562 | 0.08 | 0.02 | compact | 0 | 3 |
| JSV-563 | 0.04 | 0.05 | compact | 1 | 2 |
| JSV-564 | 0.07 | 0.02 | compact | 1 | 3 |
| JSV-565 | 0.05 | 0.03 | tabular | 2 | 3 |
| JSV-566 | 0.05 | 0.03 | compact | 2 | 3 |
| JSV-567 | 0.06 | 0.04 | compact | 3 | 2 |
| JSV-568 | 0.07 | 0.02 | compact | 3 | 3 |
| JSV-569 | 0.06 | 0.02 | compact | 2 | 2 |
| JSV-570 | 0.03 | 0.01 | compact | 1 | 3 |
| JSV-571 | 0.08 | 0.02 | compact | 1 | 3 |
| JSV-572 | 0.1 | 0.02 | tabular | 1 | 3 |
| JSV-573 | 0.07 | 0.05 | compact | 1 | 3 |
| JSV-574 | 0.08 | 0.03 | tabular | 2 | 3 |
| JSV-575 | 0.06 | 0.03 | tabular | 2 | 2 |
| JSV-576 | 0.06 | 0.06 | compact | 2 | 2 |
| JSV-577 | 0.06 | 0.04 | compact | 3 | 2 |
| JSV-578 | 0.06 | 0.05 | tabular | 2 | 3 |
| JSV-579 | 0.07 | 0.04 | compact | 1 | 3 |
| JSV-580 | 0.07 | 0.05 | compact | 1 | 3 |
| JSV-581 | 0.07 | 0.04 | compact | 3 | 2 |
| JSV-582 | 0.11 | 0.03 | tabular | 1 | 3 |
| JSV-583 | 0.14 | 0.05 | elongate | 0 | 3 |
| JSV-584 | 0.09 | 0.03 | elongate | 0 | 3 |
| JSV-585 | 0.07 | 0.03 | tabular | 2 | 3 |
| JSV-586 | 0.07 | 0.03 | compact | 1 | 3 |
| JSV-587 | 0.03 | 0.02 | compact | 2 | 2 |
| JSV-588 | 0.06 | 0.02 | elongate | 1 | 1 |
| JSV-589 | 0.06 | 0.02 | compact | 1 | 2 |
| JSV-590 | 0.04 | 0.02 | compact | 1 | 3 |
| JSV-591 | 0.06 | 0.02 | compact | 2 | 1 |
| JSV-592 | 0.05 | 0.02 | tabular | 2 | 1 |
| JSV-593 | 0.04 | 0.04 | compact | 1 | 2 |
| JSV-594 | 0.08 | 0.03 | tabular | 1 | 3 |
| JSV-595 | 0.09 | 0.04 | compact | 3 | 3 |
| JSV-596 | 0.06 | 0.05 | compact | 2 | 2 |
| JSV-597 | 0.06 | 0.04 | compact | 2 | 3 |
| JSV-598 | 0.07 | 0.04 | compact | 1 | 3 |
| JSV-599 | 0.06 | 0.04 | compact | 2 | 3 |
| JSV-600 | 0.06 | 0.02 | compact | 2 | 2 |
| JSV-601 | 0.07 | 0.02 | compact | 3 | 3 |
| JSV-602 | 0.1 | 0.02 | tabular | 1 | 3 |
| JSV-603 | 0.05 | 0.02 | compact | 0 | 3 |
| JSV-604 | 0.05 | 0.02 | compact | 1 | 1 |
| JSV-605 | 0.07 | 0.05 | compact | 2 | 1 |
| JSV-606 | 0.07 | 0.03 | compact | 2 | 2 |
| JSV-607 | 0.08 | 0.03 | tabular | 2 | 3 |
| JSV-608 | 0.17 | 0.03 | tabular | 1 | 1 |
| JSV-609 | 0.05 | 0.03 | compact | 2 | 3 |
| JSV-610 | 0.08 | 0.01 | elongate | 0 | 1 |
| JSV-611 | 0.11 | 0.02 | elongate | 1 | 3 |
| JSV-612 | 0.05 | 0.02 | tabular | 2 | 1 |
| JSV-613 | 0.06 | 0.02 | compact | 2 | 3 |
| JSV-614 | 0.09 | 0.04 | compact | 1 | 3 |
| JSV-615 | 0.06 | 0.02 | tabular | 3 | 1 |
| JSV-616 | 0.09 | 0.02 | compact | 2 | 2 |
